# ZBED6, a Novel Transcription Factor Derived from a Domesticated DNA Transposon Regulates IGF2 Expression and Muscle Growth

**DOI:** 10.1371/journal.pbio.1000256

**Published:** 2009-12-15

**Authors:** Ellen Markljung, Lin Jiang, Jacob D. Jaffe, Tarjei S. Mikkelsen, Ola Wallerman, Martin Larhammar, Xiaolan Zhang, Li Wang, Veronica Saenz-Vash, Andreas Gnirke, Anders M. Lindroth, Romain Barrés, Jie Yan, Sara Strömberg, Sachinandan De, Fredrik Pontén, Eric S. Lander, Steven A. Carr, Juleen R. Zierath, Klas Kullander, Claes Wadelius, Kerstin Lindblad-Toh, Göran Andersson, Göran Hjälm, Leif Andersson

**Affiliations:** 1 Department of Medical Biochemistry and Microbiology, Uppsala University, Uppsala, Sweden; 2 Broad Institute of Harvard and Massachusetts Institute of Technology, Cambridge, Massachusetts, United States of America; 3 Department of Genetics and Pathology, The Rudbeck Laboratory, Uppsala University, Uppsala, Sweden; 4 Department of Neuroscience, Uppsala University, Uppsala, Sweden; 5 Department of Animal Breeding and Genetics, Swedish University of Agricultural Sciences, Uppsala, Sweden; 6 Department of Molecular Medicine and Surgery, Section of Integrative Physiology, Karolinska Institutet, Stockholm, Sweden; University of Cambridge, United Kingdom

## Abstract

A single nucleotide substitution in intron 3 of *IGF2* in pigs abrogates a binding site for a repressor and leads to a 3-fold up-regulation of *IGF2* in skeletal muscle. The mutation has major effects on muscle growth, size of the heart, and fat deposition. Here, we have identified the repressor and find that the protein, named ZBED6, is previously unknown, specific for placental mammals, and derived from an exapted DNA transposon. Silencing of *Zbed6* in mouse C2C12 myoblasts affected *Igf2* expression, cell proliferation, wound healing, and myotube formation. Chromatin immunoprecipitation (ChIP) sequencing using C2C12 cells identified about 2,500 ZBED6 binding sites in the genome, and the deduced consensus motif gave a perfect match with the established binding site in *Igf2*. Genes associated with ZBED6 binding sites showed a highly significant enrichment for certain Gene Ontology classifications, including development and transcriptional regulation. The phenotypic effects in mutant pigs and ZBED6-silenced C2C12 myoblasts, the extreme sequence conservation, its nucleolar localization, the broad tissue distribution, and the many target genes with essential biological functions suggest that ZBED6 is an important transcription factor in placental mammals, affecting development, cell proliferation, and growth.

## Abstract 2

This study identifies a previously uncharacterized protein, encoded by a domesticated DNA transposon, called ZBED6 that regulates the expression of the insulin-like growth factor 2 (*IGF2*) gene, and possibly numerous others, in all placental mammals including human.

## Author Summary

The molecular identification of genes and mutations affecting complex traits and disorders has proven to be very challenging in humans as well as in model organisms. These so-called quantitative traits arise from interactions between two or more genes and their environment, and can be mapped to their underlying genes via closely linked stretches of DNA called quantitative trait loci (QTL). Previously, we identified a single nucleotide substitution in a noncoding region of the insulin-like growth factor 2 gene (*IGF2*) in pigs that is underlying a major QTL affecting muscle growth, heart size, and fat deposition. The mutation disrupts interaction with an unknown nuclear protein acting as a repressor of *IGF2* transcription. In the present study, we have isolated a zinc finger protein of unknown function and show that it regulates the expression of *IGF2*. The protein, which we named ZBED6, is encoded by a domesticated DNA transposon that was inserted into the genome prior to the radiation of placental mammals. ZBED6 is exclusive to placental mammals and highly conserved among species. Our functional characterization of ZBED6 shows that it has a broad tissue distribution and may affect the expression of thousands of other genes, besides *IGF2*, that control fundamental biological processes. We postulate that ZBED6 is an important transcription factor affecting development, cell proliferation, and growth in placental mammals.

## Introduction

Strong selection for lean growth in the domestic pigs during the last 60 years has resulted in increased muscle growth and reduced fat deposition. Quantitative trait locus (QTL) mapping using an intercross between the European Wild Boar and Large White domestic pigs identified the most important locus that has responded to this selection pressure as a paternally expressed QTL colocalized with the gene for insulin-like growth factor 2 (*IGF2*) [1]. The allele present in the domestic pig increases muscle growth and heart size, and reduces subcutaneous fat deposition. The causative mutation for this QTL is a single nucleotide substitution in intron 3 of *IGF2* [2]. The mutation is located in a CpG island that is well-conserved among mammals, and 16 bp including the mutated site showed 100% sequence identity among eight placental mammals. This quantitative trait nucleotide (QTN) is one of the rare examples in which a single base substitution underlying a complex trait has been identified and the mechanism of action is partially understood [2].

The *IGF2* mutation, a G to A transition, disrupts the interaction with an unknown nuclear factor, a repressor, and leads to a 3-fold up-regulation of *IGF2* expression in skeletal muscle. Elevated paternal expression from the mutant allele increases skeletal muscle mass and thus meat production by 3%–4%. The favorable allele has undergone a massive selective sweep and is close to fixation in pig populations widely used for meat production. Pigs carrying the favorable allele at the paternal chromosome show higher expression from the *IGF2* P2, P3, and P4 promoters in skeletal and cardiac muscle, but not in liver. Importantly, this up-regulated *IGF2* expression occurs postnatally, but not in fetal muscle. The mutation also up-regulates expression of an *IGF2* antisense noncoding transcript with hitherto unknown function [3]. Thus, the binding of the repressor to its target site represses transcription from at least four promoters spread over a 4-kb region. Furthermore, the repressor binds its target site only when it is unmethylated [2].

Here, we report the identification of the repressor binding the *IGF2* QTN site using mass spectrometry analysis after capturing nuclear proteins using a biotinylated oligonucleotide corresponding to the wild-type sequence. The protein, named ZBED6, is previously unknown and is encoded by an exapted DNA transposon. Elucidation of its functional role is shown by small interfering RNA (siRNA) and transient transfection using *IGF2* P3 reporters.

## Results

### Identification of the *IGF2* Repressor Using Oligonucleotide Capture and Mass Spectrometry

Our previous electrophoretic mobility shift assay (EMSA), as well as transient transfection experiments with luciferase reporters, demonstrated that the unknown repressor is expressed in mouse C2C12 myoblasts [2]. To isolate the *IGF2* repressor, we employed affinity capture using nuclear extracts from C2C12 cells and biotinylated oligonucleotides corresponding to the wild-type (*q*) and mutant (*Q*) sequence where only the former binds the repressor. Two different nuclear extracts were prepared using stable isotope labeling of amino acids in culture (SILAC) technique [4], in which the “heavy” extract proteins contained the stable-isotope–labeled amino acids lysine and arginine, whereas the “light” extract proteins contained the natural versions of these amino acids. The wild-type and mutant oligonucleotides were incubated with heavy and light extracts, respectively. Captured heavy and light proteins were mixed and separated with SDS-PAGE. Gel slices were digested with trypsin, and the resulting peptides were analyzed by liquid chromatography mass spectrometry (LCMS). The acquired spectra were searched against the RefSeq database containing mouse protein sequences to identify proteins present in the sample. Ratios of the amount of each protein enriched by the *q* and *Q* constructs were computed by comparing the mass spectral signals from the heavy and light versions of each identified peptide composing the protein.

**Figure 1:**
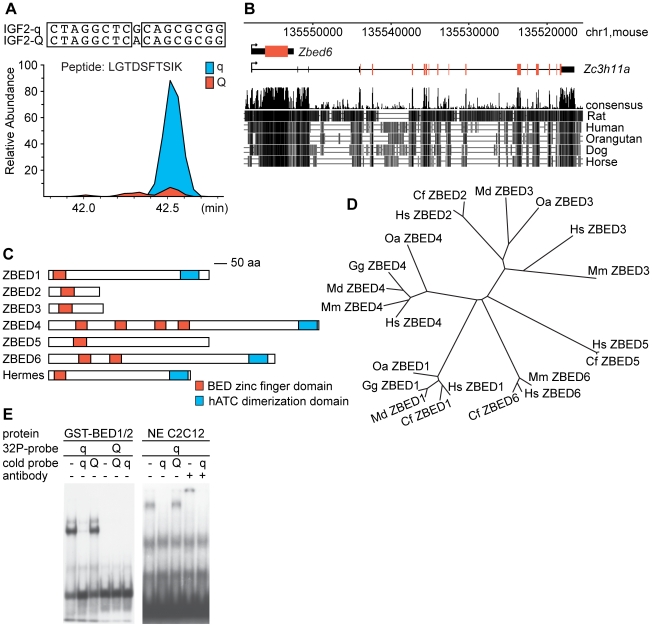
Identification of ZBED6. (A) Mass spectrometric quantification of ZBED6-enrichment using SILAC; the core sequences of the wild-type q and the mutant Q oligonucleotides used for protein capture are shown on top. ZBED6 was identified by six unique peptides in the SILAC-heavy *q* sample. Two peptides were simultaneously observed in the SILAC-light *Q* sample and were used as the basis for SILAC quantification. A representative peptide is shown where the area under the curve corresponds to the amount of ZBED6 enriched by the *q* probe (blue) and the *Q* probe (red). The average overall enrichment of ZBED6 by *q* was 9.0-fold (±1.2-fold, *n* = 2 experiments). (B) Genome organization of *Zbed6* and *Zc3h11a* in mouse, adapted from the UCSC Genome Browser (http://genome-test.cse.ucsc.edu/). Untranslated and translated exons of *Zc3h11a* are indicated by black and red bars, respectively. A mammalian conservation track from the UCSC browser is included at the bottom. (C) Schematic representation of the six ZBED family members present in human and the *Hermes* hAT DNA transposase from the house fly. (D) Phylogenetic analysis of ZBED protein sequences from selected species. Cf (*Canis familiaris*; dog), Gg (*Gallus gallus*; chicken), Hs (*Homo sapiens*; human), Md (*Monodelphis domestica*; opossum), Mm (*Mus musculus*; house mouse), and Oa (*Ornithorhynchus anitinus*; platypus). *ZBED1* and *ZBED2* homologs were not found in the mouse genome, and mouse *Zbed5* contains numerous mutations and stop codons efficiently destroying the reading frame. (E) EMSA showing binding of recombinant ZBED6 BED1/2 domains to wild-type (q), but not to mutant (Q) probe (left panel). Likewise, endogenous ZBED6 in nuclear extracts (NE) from C2C12 cells forms a complex with wild-type probe that is competed by q, but not Q probe (right panel). The complex is supershifted by the anti-ZBED6 antibody, and competition confirmed the specific interaction with ZBED6.

The protein demonstrating the highest fold enrichment by *q* (9.0±1.2-fold; Figure 1A) corresponded to a transcript annotated as an alternative splice form of the poorly characterized *Zc3h11a* gene. ZC3H11A belongs to a large family of zinc finger proteins with 58 known members in mouse [5]. However, a closer examination revealed that the captured peptide is encoded by an intronless gene located in intron 1 of *Zc3h11a* (Figure 1B). The gene contains an open reading frame of more than 900 codons and encodes a protein with no sequence similarity to ZC3H11A. The encoded protein contains two BED domains and an hATC dimerization domain (Figure 1C). The BED domain was originally identified by a bioinformatic analysis using two chromatin-boundary-element-binding proteins from *Drosophila*, BEAF and DREF, as seeds for homology search [6]. We named our protein ZBED6 because it is the sixth mammalian protein with one or more BED domains (Figure 1C and 1D). *ZBED6* is related to the hAT superfamily of DNA transposons, named after *hobo* from *Drosophila*, *Activator* from maize, and *Tam3* from snapdragon [7]. For instance, the active *Hermes* transposase from the housefly contains an amino-terminal BED domain and a carboxyterminal hATC domain (Figure 1C).

The mammalian ZBED proteins showed high sequence divergence outside the BED and hATC domains and represent divergent members of the hAT superfamily (unpublished data). The two BED domains of ZBED6 are more closely related to each other than to any other mammalian BED sequence, implying an internal duplication after the integration in the genome (Figure S1). The ZBED6 protein is highly conserved among placental mammals, and in particular, the DNA-binding BED domains show 100%, or close to 100%, sequence identity among 26 different species, including the pig (Figure S2); the only sequence differences compared with the consensus sequence for placental mammals occured in species with low-coverage genome sequences and may therefore represent sequence errors.

To address whether ZBED6 is the bona fide repressor binding the QTN region in *IGF2* intron 3, we produced a truncated mouse ZBED6 protein including the two DNA-binding BED domains and used this in EMSA with the q and Q oligonucleotides differing only at the QTN position. EMSA revealed a highly specific interaction with the wild-type q oligonucleotide, and a 100-fold excess of mutant Q oligonucleotide could not outcompete the interaction (Figure 1E). A polyclonal anti-ZBED6 antibody was developed by immunizing rabbits with this recombinant protein containing the two BED domains. A supershift was obtained when nuclear extracts from C2C12 cells were incubated with this anti-ZBED6 antibody, providing further biochemical evidence for ZBED6 as the elusive *Igf2* repressor (Figure 1E).

### *Zbed6* Has a Broad Tissue Distribution and Is Coexpressed with *Zc3h11a*

**Figure 2:**
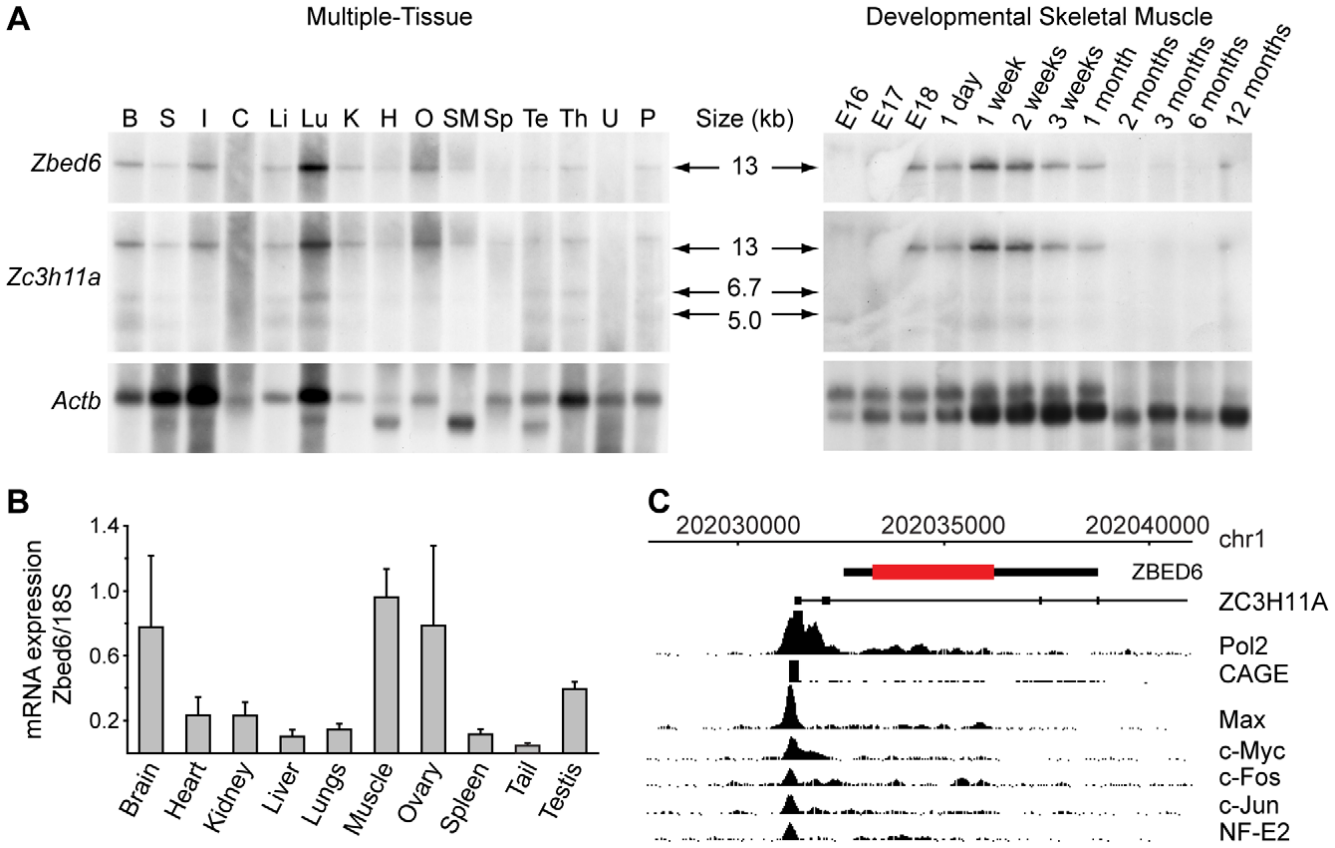
Expression pattern of *Zbed6*. (A) Multiple tissue and developmental skeletal muscle Northern blot analysis of *Zbed6* and *Zc3h11a*; β-actin (Actb) was used as control. The *Zbed6* and *Zc3h11a* probes apparently hybridized to the same transcript, whereas *Zc3h11a* also hybridized to shorter alternative transcripts lacking *Zbed6*. The estimated molecular weights are indicated. B, brain (whole); C, colon; H, heart; I, intestine (whole); K, kidney; Li, liver; Lu, lung; O, ovary; P, placenta (late pregnancy); S, stomach (whole); SM, skeletal muscle; Sp, spleen; Te, testis; Th, thymus; U, uterus (nonpregnant). (B) Real-time-PCR analysis of *Zbed6* in several mouse tissues. *Zbed6* mRNA expression was normalized using *18S-rRNA*. Error bars represent standard error of the mean (s.e.m.). (C) ChIP sequencing data for the *ZC3H11A/ZBED6* upstream region generated by the ENCODE project using the K-562 erythroleukemia cell line; adapted from the UCSC Genome Browser (http://genome-test.cse.ucsc.edu/). The location of RNA polymerase II binding sites (Pol2), a 5′ cap analysis gene expression tag (CAGE), binding sites for the Max, Myc, Fos, Jun, and NF-E2 transcription factors are indicated.

Northern blot analysis (Figure 2A) and real-time PCR (RT-PCR) analysis (Figure 2B) showed that *Zbed6*, like *Igf2*, has a broad tissue distribution in mouse and is expressed in skeletal muscle, consistent with it being the *Igf2* repressor. Northern blot analysis (Figure 2A) and RT-PCR amplification and sequencing (unpublished data) revealed that *Zbed6* is coexpressed with *Zc3h11a* as an ∼13-kb splice variant of *Zc3h11a*, retaining the genomic region from exon 1 to exon 4 (including introns 1, 2, and 3 with *Zbed6* located in intron 1) spliced to the remaining *Zc3h11a* exons (exons 5–18).

An examination of chromatin immunoprecipitation (ChIP) sequencing data for the human genome provided by the ENCODE consortium showed that there is one major RNA polymerase II binding site located just upstream of exon 1 of *ZC3H11A*, which apparently constitutes the common promoter for *ZC3H11A* and *ZBED6* (Figure 2C). This is further supported by the perfect colocalization with a 5′ cap analysis gene expression (CAGE) tag. This promoter region contains binding sites for the Max, Myc, Fos, Jun, and NF-E2 transcription factors, all with a central role in regulating cell proliferation and associated with cancer development.

**Figure 3:**
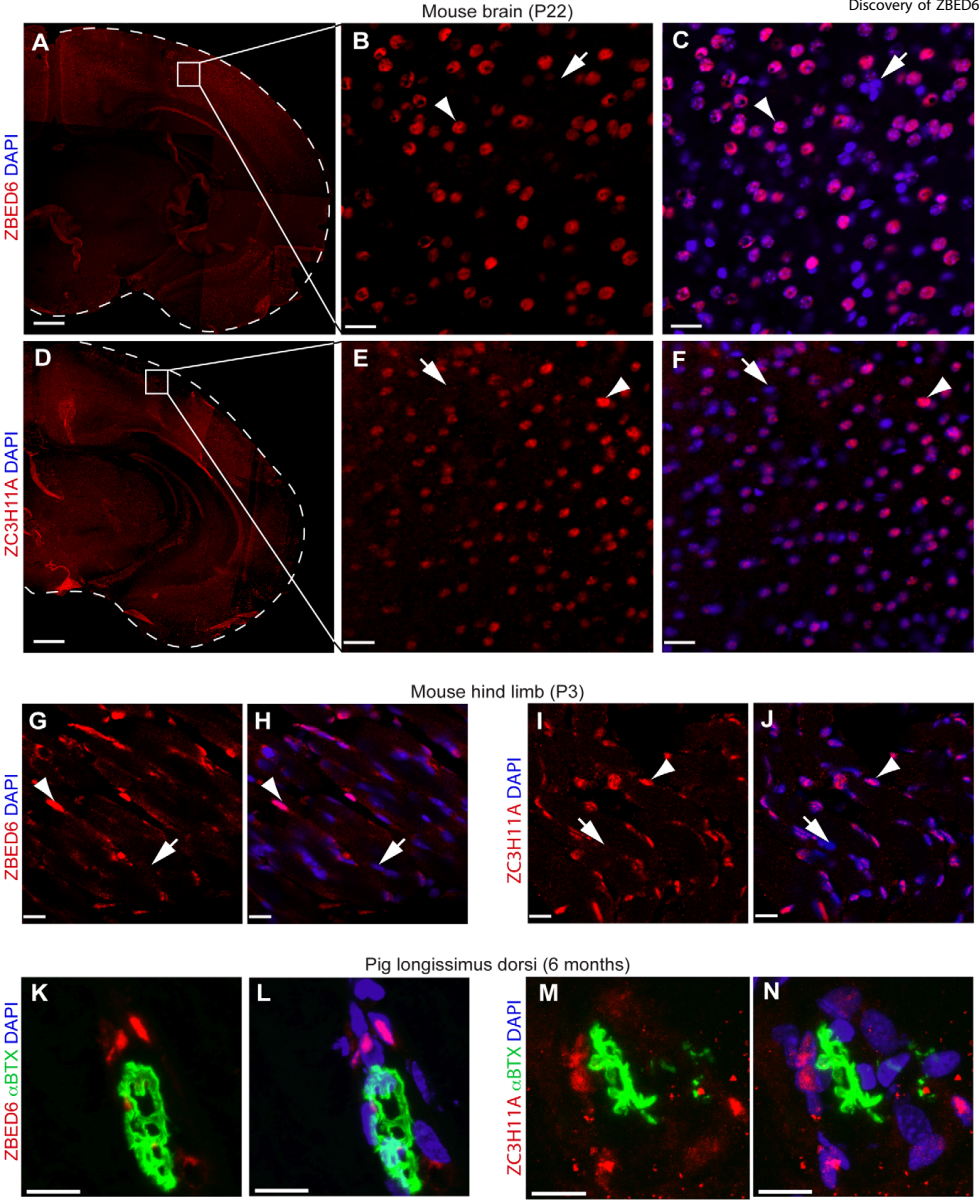
Immunohistochemistry of ZBED6 and ZC3H11A in brain and muscle tissue from mouse and in pig muscle. (A–J) Immunofluorescence analysis of mouse tissues stained with anti-ZBED6 or anti-ZC3H11A antibodies. (A and D) Coronal brain overview images assembled from pictures taken at 4× magnification. (B, C, E, and F) Higher magnification images of boxed areas indicated in (A and D) with anti-ZBED6 (B and C) or anti-ZC3H11A (E, F) immunopositive signals in red. (G–J) Images of anti-ZBED6 or anti-ZC3H11A antibody stainings of section from mouse thigh muscle. Nuclear localization of ZBED6 and ZC3H11A is confirmed by colabeling with DAPI (blue) in both brain and muscle as indicated by arrowheads, whereas nuclei lacking ZBED6- or ZC3H11A-positive signals are indicated by arrows. (K–N) Immunofluorescence analysis of pig muscle tissue (*longissimus dorsi*) stained with anti-ZBED6 (K and L) or anti-ZC3H11A (M and N). α-Bungarotoxin fused to a fluorescent dye (αBTX, green) was used to confirm identification of skeletal muscle tissue since this toxin binds cholinergic receptors in neuromuscular junctions. Scale bars indicate 480 µm in (A and D), 22 µm in (B, C, E, and F), and 14 µm in (G–N).

ZBED6 and ZC3H11A proteins were detected in the nuclei of postnatal muscle and brain tissue from mouse by using immunohistochemistry (Figure 3). ZBED6 expression was also confirmed in skeletal muscle (*longissimus dorsi*) cell nuclei from a 6-mo-old pig (Figure 3K–3N), i.e., at the age at which a highly significant effect of the pig *IGF2* mutation is documented [1],[2].

### The ZBED6 Protein Shows a Nucleolar Localization in Mouse C2C12 Cells

**Figure 4:**
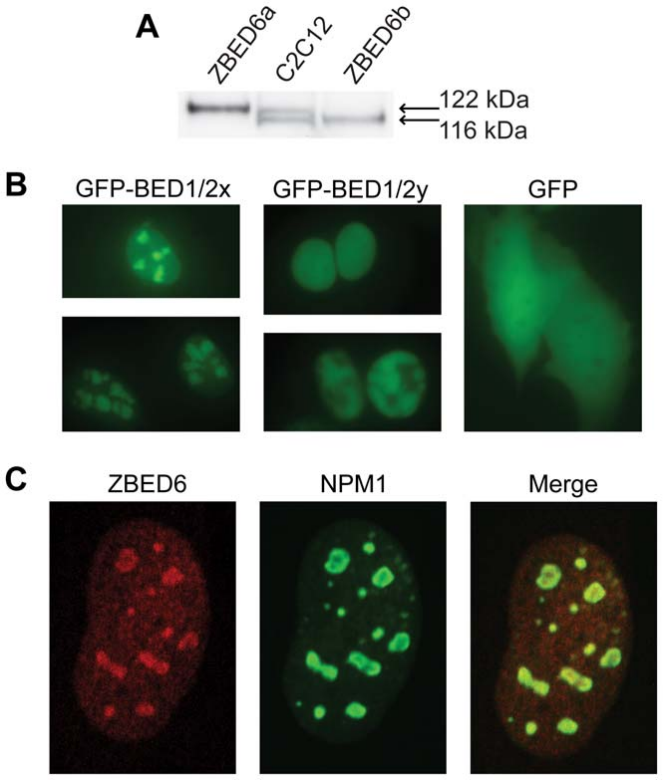
Detection of ZBED6 protein and nucleolar localization. (A) Western blot analysis of recombinant protein (ZBED6a and ZBED6b) and C2C12 protein extracts using the polyclonal antibody raised against the ZBED6 BED domains. Two isoforms, ZBED6a and ZBED6b, with apparent molecular weights of 122 kDa and 116 kDa (calculated molecular weights 109 kDa and 104 kDa, respectively) corresponding to two predicted alternative translation start sites were detected in C2C12 protein extracts. (B) Nuclear localization of ZBED6 in C2C12 cells. Constructs containing ZBED6 BED1/2 domains fused to GFP compared with GFP alone. The constructs GFP-BED1/2x and GFP-BED1/2y contained amino acid residues 47–384 and 90–384 of the ZBED6a isoform, respectively. (C) Immunofluorescence analysis of C2C12 cells stained with anti-ZBED6 (red) and anti-nucleophosmin 1 (Npm1; green) antibodies.

Western blot analysis of proteins from mouse C2C12 cells revealed two different isoforms, denoted ZBED6a and ZBED6b, with apparent molecular weights of 122 and 116 kDa, respectively (Figure 4A). These isoforms most likely correspond to the use of two alternative start codons in the open reading frame of *Zbed6*, as demonstrated by comigration with recombinant proteins representing the two isoforms (Figure 4A).

Bioinformatic analysis revealed bipartite nuclear localization signals in two regions of ZBED6, amino acid residues 61–80 and 231–248. We used two different GFP-fusion constructs to confirm a nuclear localization in C2C12 cells (Figure 4B). The GFP-BED1/2x protein (residues 47–384) containing both regions with nuclear localization signals was primarily associated with granular structures in the nucleus, suggesting a nucleolar localization. This was confirmed by double immunofluorescence staining of C2C12 cells (Figure 4C) showing colocalization of endogenous ZBED6 and nucleophosmin 1, a well-known marker for the nucleolus [8]. However, ZBED6 did not show a complete localization to the nucleolus since some dispersed staining throughout the nucleus was evident (Figure 4C). Interestingly, the GFP-BED1/2y fusion protein (residues 90–384) also showed nuclear localization, but as an exclusion from the nucleolus (Figure 4B). This implies that the nuclear localization signal comprising residues 61–80 constitutes or contains a nucleolar localization signal. This lysine- and arginine-rich sequence (KKKRKKGLRIKGKRRRKKLI) is highly positively charged and resembles a nucleolar localization signal previously identified in the myogenic regulatory factor Myf5 [9]. The nucleolar localization of ZBED6 is of considerable interest in relation to the phenotype of *IGF2*-mutant pigs because the function of the nucleolus is associated with regulation of cell growth and proliferation [10].

### Silencing of *Zbed6* Confirms Specific Interaction with the *Igf2* QTN Site

**Figure 5:**
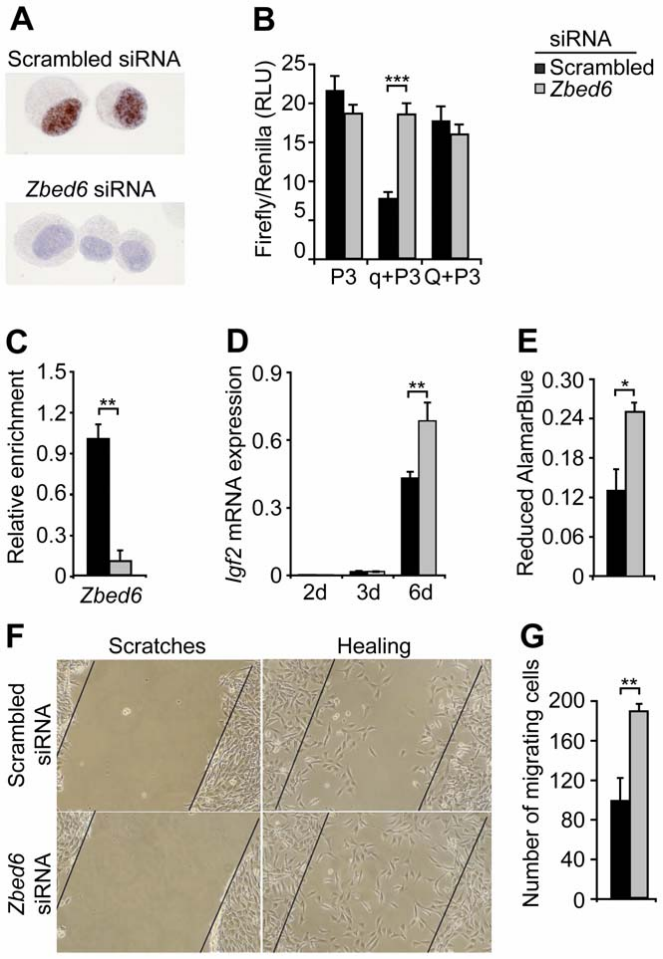
RNAi-mediated *Zbed6* silencing in C2C12 cells. (A) Immunocytochemical staining of cytospins of C2C12 cells with anti-ZBED6 antibody (brown) and nuclear counterstain (blue). (B) Luciferase assays of reporter constructs containing the wild-type q or mutant Q sequence of pig *IGF2* intron 3 and the pig *IGF2* P3 promoter. Firefly reporter luciferase levels in relation to control Renilla luciferase level. (C) RT-PCR analysis of the *Igf2* intron 3 region after chromatin immunoprecipitation (ChIP) with anti-ZBED6. (D) *Igf2* mRNA expression at days 2, 3, and 6 after transfection. (E) Cell proliferation at day 3 after silencing. (F) Monolayer scratch wound healing. (G) Cell number in scratches. Assays were performed 48 h (B and C) or 72 h (E and F) after transfection. At least six transfections were performed for each siRNA except for the ChIP assay that involved three replicates (A–F). Error bars indicate s.e.m. (*p<0.05; **p<0.01; ***p<0.001).

*Zbed6* was silenced in C2C12 cells by using siRNA to obtain further insight into its functional significance. Quantitative PCR revealed a >75% decrease in detected *Zbed6* mRNA, and immunocytochemistry further confirmed a highly efficient silencing at the protein level (Figure 5A). *Zbed6*-silenced and control C2C12 cells were used to repeat our previously described luciferase assay including a reporter construct containing either the wild-type or mutant sequence of the QTN region fused with the *IGF2* P3 promoter [2]. An assay based on C2C12 control cells transfected with scrambled oligonucleotide replicated our previous results since a construct containing the wild-type QTN region repressed luciferase expression in comparison with a construct containing P3 alone, whereas a construct including the mutant QTN region was associated with no or only minor repression (Figure 5B). In contrast, transfection experiments using three different silencing oligonucleotides directed against *Zbed6* mRNA completely abolished repression with the wild-type q construct (Figure 5B). The interaction between ZBED6 and the QTN site in *Igf2* intron 3 in mouse C2C12 cells was also validated by ChIP analysis using our anti-ZBED6 antibody, as a significant reduction in the enrichment of this region was observed after *Zbed6* silencing (Figure 5C).

### Silencing of *Zbed6* Is Associated with Elevated *Igf2* Expression, Increased Cell Proliferation, and a Faster Wound Healing Process

ZBED6 function was further investigated by specific gene silencing in C2C12 cells that were induced to differentiate by changing from growth to differentiation medium. *Igf2* mRNA expression was low in both control and *Zbed6*-silenced cells the first days after differentiation was induced (Figure 5D). However, at day 6, *Igf2* mRNA expression was significantly increased in silenced cells compared with controls (Figure 5D). This result is consistent with the increased *IGF2* expression in skeletal muscle of pigs carrying the mutation at the ZBED6 target site in *IGF2* intron 3 [2].

Silencing of *Zbed6* was also accompanied by increased cell proliferation (Figure 5E), a faster formation of myotubes (Figure S3), and a faster wound healing process after scratching the surface of growing C2C12 cells (Figure 5F and 5G). A faster wound healing may reflect increased cell proliferation and/or increased cell migration. The fact that increased cell proliferation and faster wound healing were observed at day 3 after silencing, when there was not yet any significant effect on *Igf2* expression (Figure 5D), implies that these effects are mediated by other target genes controlled by ZBED6.

### ChIP Sequencing Indicates That ZBED6 Regulates Many Genes of Importance for Development and Transcription

**Figure 6:**
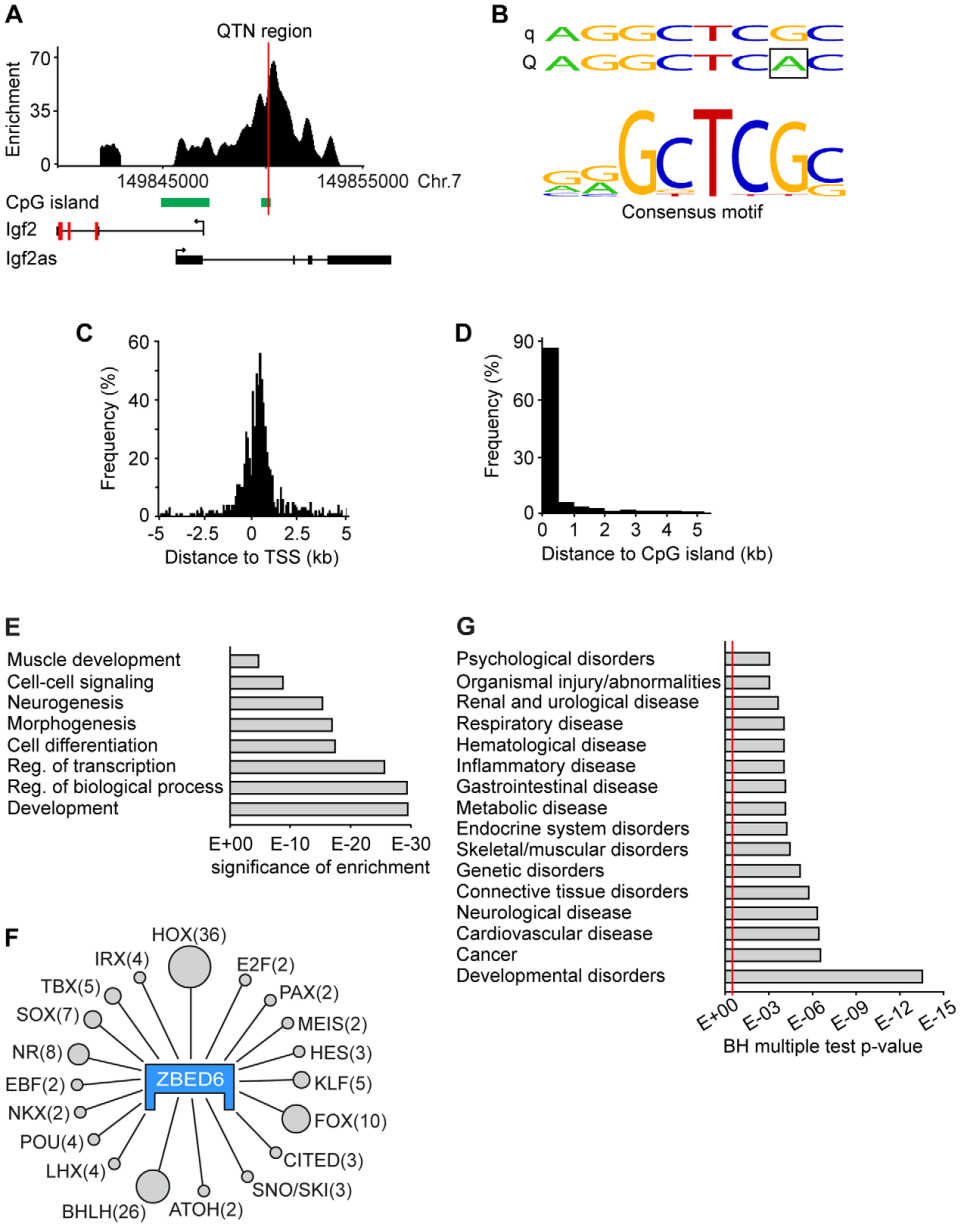
ChIP sequencing using mouse C2C12 cells. (A) ChIP-sequencing peak around the quantitative trait nucleotide (QTN) position in an intron of *Igf2*. (B) ZBED6 binding motif in *Igf2* and consensus ZBED6 motif based on peaks within 5 kb of transcription start sites (TSS) and with at least 25 overlapping fragments but excluding the binding site at *Igf2*. (C and D) ChIP-sequencing peaks are preferentially located in the near vicinity of (C) TSS and (D) CpG islands. (E) Gene Ontology categories highly enriched among the genes bound by ZBED6. Ontology terms are shown on the *y* axis; *p*-values for the significance of enrichment are on the *x* axis. (F) Overrepresented transcription factor families bound by ZBED6, including homeobox proteins (HOX), paired-like (PAX), HOX factors (MEIS), hairy and enhancer of split protein (HES), krueppel C2H2 zinc finger (KLF), Forkhead box (FOX), cbp/p300-interacting transactivator (CITED), SNO and SKI oncogenes (SNO/SKI), atonal homolog (ATOH), basic helix-loop-helix (BHLH), LIM homeobox (LHX), POU domain containing classes 3 and 4 (POU), NK transcription factor related (NKX), early B-cell factor (EBF), nuclear receptor (NR), SRY box (SOX), T-box (TBX), and Iroquois homeobox protein (IRX). The number of transcription factor genes associated with ZBED6 binding sites is indicated beside each family name. (G) Results of Ingenuity Pathways Analysis showing that putative downstream targets of ZBED6 are significantly associated with different diseases in humans. *p*-values for nonrandom association were calculated using the Fisher exact test, followed by a Benjamini-Hochberg correction for multiple testing. The red line indicates the 5% significance threshold.

A ChIP-sequencing experiment using our anti-ZBED6 antibody was performed to search for other downstream targets of ZBED6 besides *Igf2*. Mouse C2C12 cells were used for this experiment as ZBED6 is expressed in this cell line and interacts with the *Igf2* QTN site, providing a positive control. The AB SOLiD technology was used to sequence the ChIP DNA fragments which resulted in the generation of 24 million reads aligned to the mouse genome. An analysis of these data revealed 2,499 peaks with a minimum of 15 overlapping extended reads (Table S1). As expected, the region in *Igf2* corresponding to the porcine QTN site was among the most highly enriched regions (Figure 6A). De novo motif searches on both the full dataset and on subsets divided by enrichment levels gave a perfect match to the QTN site in *Igf2* (Figure 6B). The result implies that the majority of the 2,499 peaks represent authentic target sites interacting with ZBED6 in C2C12 cells. Functional support for this is that the ZBED6 peaks often occurred in the vicinity of known transcription start sites (TSS), with approximately 50% of the peaks located within 5 kb of TSS (Figure 6C). Interestingly, there was a clear bias of binding sites to be located downstream of TSS, and a large proportion was found in intron 1, suggesting a role in transcriptional silencing. The *IGF2* site in pigs is within a CpG island [2], and in this study, we found an enrichment of peaks close to CpG islands (Figure 6D), with as many as 28% of all peak maxima within a CpG island. In comparison only 16% of the locations of the 5′-GCTCGC-3′ consensus sequence occur in CpG islands in the mouse genome, indicating that other sequence elements are required for binding.

About 1,200 annotated genes were associated with one or more putative ZBED6 binding sites located within 5 kb of the gene, and 255 genes were associated to peaks of similar or higher enrichment as was seen for *Igf2*. We used this list of ∼1,200 genes to search for an enrichment of specific Gene Ontology classifications. The analysis showed that genes associated with development, regulation of biological processes, transcriptional regulation, cell differentiation, morphogenesis, neurogenesis, cell–cell signaling, and muscle development were all highly enriched in this list (Figure 6E). The list included 262 genes encoding transcription factors and certain families of transcription factors were particularly abundant among the putative ZBED6 targets (Figure 6F).

### Human Orthologs of Mouse ZBED6 Targets Are Significantly Overrepresented among Disease-Causing Genes

The ChIP-sequencing results indicated that ZBED6 takes part in the regulation of genes associated with basal functions in placental mammals. Therefore, we decided to perform an Ingenuity Pathways Analysis (Ingenuity Systems, http://www.ingenuity.com) to test whether putative ZBED6 targets are overrepresented among disease-associated genes in humans. This was accomplished by first downloading a table with all established mouse–human orthologs (Mouse Genome Informatics, http://www.informatics.jax.org/orthology.shtml) and then this list of human orthologs was used for the analysis. The results showed a highly significant association between our putative ZBED6 target genes and a number of diseases (Figure 6G). The most significant association was observed for developmental disorders, consistent with the Gene Ontology analysis, followed by cancer, cardiovascular disease, and neurological disease.

## Discussion

Our quest for the nuclear factor binding the QTN site in the porcine *IGF2* gene has been driven by the vision that this factor must be important, since disruption of the interaction with one of its target sites alters body composition and promotes cardiac growth in pigs. Our previous experiments revealed a highly specific interaction between the factor and its target site in *IGF2* [2], but it was not until we used the ultrasensitive SILAC technology that we could take advantage of this specificity and isolate ZBED6. The results presented in this study have conclusively demonstrated that ZBED6 is the bona fide repressor binding the QTN site in pig *IGF2*. This conclusion is based on (*i*) EMSA with recombinant ZBED6 protein, (*ii*) supershift of EMSA complex using an anti-ZBED6 antibody, (*iii*) abolishment of the repressor function in a luciferase assay after siRNA silencing, and (*iv*) ChIP data. The biological significance of ZBED6 was underscored in this study as siRNA silencing in C2C12 cells led to faster myotube formation and wound healing, and increased cell proliferation.

The difference between less complex eukaryotes like *Caenorhabditis elegans* and more complex eukaryotes, such as human, is related not to the number of protein-coding genes, but rather to the complexity of the gene regulatory networks. A large proportion of vertebrate genomes is composed of transposable elements, and their integration in the genome has contributed to the evolution of regulatory networks [11]. The majority of these transposable elements are retrotransposons, but 5%–10% are derived from DNA transposons. In the initial analysis of the first human genome assembly, Lander et al. [12] identified 47 human genes derived from transposable elements, as many as 43 of these are derived from DNA transposons, and in fact, one of the genes listed in Table 13 of the human genome paper corresponds to *ZBED6*. However, *ZBED6* has never been appropriately annotated in any mammalian genome, despite the fact that it constitutes an ∼2,900-bp open reading frame and that part of the ZBED6 protein is extremely well conserved among placental mammals. A bioinformatic analysis of other vertebrate genomes did not reveal the presence of a functional *ZBED6* gene outside the placental mammals. We found evidence for a nonfunctional *ZBED6* sequence at the orthologous positions in the Platypus and opossum genomes, but these genomes did not contain an extended open reading frame for *ZBED6* (unpublished data). This implies that the integration of *ZBED6* happened before the divergence of the monotremes from the other mammals, but that the gene has been inactivated or lost in monotremes and marsupials. Thus, *ZBED6* must have evolved its essential function in the time span after the split between marsupial and placental mammals, but before the radiation of different orders of placental mammals. An interesting topic for future research will be to reveal what advantage the development of ZBED6 as a new regulatory protein has provided to the placental mammals.

*ZBED6* is an apparent example of a domesticated transposon that has lost its ability to transpose, because it occurs as a single copy gene at the same location in intron 1 of *ZC3H11A* in all placental mammals for which at least a partial genome sequence is available. ZBED6 has evolved an essential function in this group as implicated by the observation that the two DNA-binding BED domains (about 100 amino acids together) show near 100% amino acid identity across 26 placental mammals (Figure S2). The two BED domains in ZBED6 have apparently evolved by internal duplication because the two copies are more similar to each other than to any other mammalian BED sequence. The mechanism by which ZBED6 acts as a repressor remains to be determined. Chromatin remodeling is an obvious possibility since other members of the ZBED family have this function. For instance, the *Drosophila* Dref protein, a BED domain protein, is found in complex with the NURF chromatin remodeling complex and its human ortholog ZBED1 interacts with MI2, a chromatin remodeling factor, and PC2, a Polycomb group protein involved in heterochromatin formation [13]. The ability of ZBED6 to interact with chromatin and affect transcriptional regulation is most likely a function derived from the ancestral transposase. The nucleolar localization of ZBED6 (Figure 4C) suggests that it may mediate transcriptional silencing by moving the *IGF2* locus and other targets to the nucleolus.

Our ChIP-sequencing experiment using mouse C2C12 myoblasts revealed more than 1,000 genes putatively regulated by ZBED6 in the mouse. We assume that a majority of these binding sites are true positives, because (*i*) we were able to generate a consensus binding motif (Figure 6B) with a perfect match with the established *Igf2* binding site using both peaks with high and low enrichment levels, (*ii*) the majority of the binding sites occurred in the vicinity of TSS (Figure 6C), (*iii*) most of the binding sites occurred within or near CpG islands (Figure 6D), in line with the established binding site in *Igf2*, and (*iv*) the highly significant enrichment of certain Gene Ontology terms (Figure 6E). Thus, although we are certain that ZBED6 interacts with a majority of the genes listed in Table S1, transcriptome analysis will be required to assess the importance of ZBED6 for transcriptional regulation of these putative targets. In this context, it is worth emphasizing that disruption of the interaction between ZBED6 and the *IGF2* QTN in pigs leads to a 3-fold up-regulation of *IGF2* mRNA in skeletal muscle and altered body composition. Interestingly, our data indicated that the *Zbed6* gene itself was bound by ZBED6 (Table S1), implying autoregulation of its expression.

About 1,200 of the ZBED6 binding sites in C2C12 cells occurred within 5 kb of the TSS of an annotated gene. The analysis of Gene Ontology terms associated with these genes revealed a highly significant enrichment for a number of important biological processes such as development, transcriptional regulation, and cell differentiation (Figure 6E). As many as 262 of the putative target genes encode transcription factors, 36 containing the homeobox domain, 26 members of the basic helix-loop-helix (bHLH) family, ten belonging to the FOX family, eight nuclear receptors, and seven members of the SOX family (Figure 6F). Many of these putative ZBED6 targets have a crucial role during development, and the results suggest that ZBED6 is an important regulator of development, cell proliferation, and growth. The binding of ZBED6 to its target sites in *IGF2* leads to repression of *IGF2* expression both in pig skeletal muscle [2] and in mouse C2C12 cells (this study). It may appear surprising that genes associated with neurogenesis were much more overrepresented in our peak list than genes associated with muscle development (Figure 6E), given the fact that we used mouse C2C12 myoblasts in this experiment. However, this pattern is expected if ZBED6 is primarily a repressor that silence genes not being part of the developmental program of a certain cell type. Another intriguing observation was the clear trend that ZBED6 preferentially binds downstream of the transcription start site which appears logical for a repressor (Figure 6C).

*Igf2* is an imprinted gene, but our list of top hits did not indicate any overrepresentation of imprinted genes. In this respect, it is noteworthy that the QTN mutation in pigs does not result in loss of imprinting, but rather exclusively increases the transcription from the paternal *Igf2* allele [2]. Thus, ZBED6 is unlikely to be a regulator of imprinting. However, one of the identified ZBED6 targets is the gene for growth factor receptor-bound protein (*Grb10*), also denoted *Meg1* (*maternally expressed gene 1*), that is maternally expressed and a potent growth inhibitor [14]. GRB10 binds to the insulin receptor (INSR) and the IGF1 receptor (IGF1R), and inhibits the growth-promoting activities of insulin (INS), IGF1, and IGF2.

The list of genes associated with ZBED6 binding sites (Table S1) includes additional members, besides *Igf2*, of the IGF-signaling pathway, namely the genes for the IGF1 receptor (*Igf1r*), IGF2 binding protein 2 (*Igf2bp2*), IGF binding protein 3 (*Igfbp3*), and IGFBP-like protein 1 (*Igfbpl1*), suggesting that ZBED6 is an important regulator of IGF signaling. Furthermore, *Grb10*, as mentioned above, also takes part in the regulation of IGF signaling [14].

Genome Wide Association (GWA) studies have revealed a number of loci in the human genome associated with multifactorial disorders (Office of Population Genomics; http://www.genome.gov/26525384). An examination of this database showed that the region harboring *ZBED6* is not one of the associated regions in any of the studies published so far. This means that the current GWA screens for different multifactorial disorders have not revealed any common *ZBED6* variants associated with disease. This does not exclude the possibility of rare sequence polymorphism in *ZBED6* affecting disease susceptibility in certain families. However, the ChIP-sequencing data indicated that ZBED6 has a fundamental role in regulating several biological processes. Mutations altering ZBED6 function or expression may therefore have severe pleiotropic effects through the many downstream targets. This notion is consistent with the near 100% conservation of the BED domains among placental mammals.

**Figure 7:**
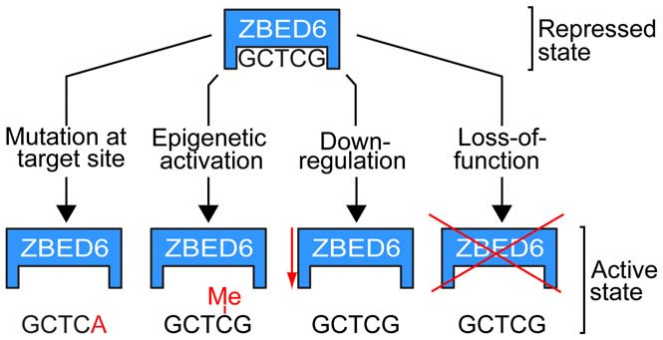
Proposed model for ZBED6 action. ZBED6 repression may be released by (*i*) germline or somatic mutations at target sites, (*ii*) methylation (Me) of the CpG site in the binding motif, (*iii*) down-regulation of ZBED6 expression, or (*iv*) loss-of-function mutations in *ZBED6*.

Our current model for ZBED6 function is summarized in Figure 7. Our data on the *IGF2* locus indicate that ZBED6 acts primarily as a repressor, likely with a modulating effect, although it is fully possible that it acts as a transcriptional activator under some circumstances.

First, germline or somatic mutations at target sites may lead to transcriptional up-regulation as demonstrated for the *IGF2* locus in pigs [2]. Our findings that the mammalian genome contains thousands of putative ZBED6 targets and that these are enriched among genes associated with disease suggest that sequence polymorphism at ZBED6 target sites may contribute significantly to variation in disease susceptibility in humans. Furthermore, the ZBED6 binding motif contains a CpG dinucleotide so we expect to find genetic polymorphisms as CpG sites are associated with a high rate of C→T and G→A transitions [15], as exemplified by the pig *IGF2* QTN. Gain or loss of ZBED6 binding sites may also have contributed to phenotypic evolution in placental mammals.

Second, our data suggest that ZBED6 targets can be released from repression by epigenetic activation. This is implied by the finding that EMSA using an oligonucleotide with a methylated CpG site was not bound by ZBED6 [2]. Interestingly, the pig QTN had no effect on *IGF2* transcription in liver, and the QTN region was shown to be methylated in this tissue, whereas it was undermethylated in skeletal muscle where the QTN had a drastic effect on *IGF2* expression [2]. Thus, epigenetic regulation of the access of ZBED6 to its target sites may play an important role during development and cell differentiation.

Third, ZBED6 targets can be released from repression by down-regulation of ZBED6 expression, as demonstrated by siRNA experiments in the present study. Finally, loss-of-function mutations in *ZBED6* are expected to up-regulate many target genes. Our finding that *Zbed6* silencing in C2C12 cells leads to faster cell proliferation and wound healing combined with the identification of a large number of cancer-associated downstream targets by ChIP sequencing implies that further studies of ZBED6 function is of considerable interest for tumor biology.

Data reported here suggest that ZBED6 has an essential role in a number of crucial gene regulatory networks. Thus, the discovery of ZBED6 opens up many avenues for research that may have profound implications for human medicine.

## Materials and Methods

### Proteomics

Two sets of murine C2C12 myoblast cells (ATCC CRL-1772) were used. Cultures were grown according to standard cell-culture procedures in SILAC-light and SILAC-heavy labeled Dulbecco's Modified Eagle Medium (DMEM, ThermoFisher) containing 10% dialyzed fetal bovine serum (FBS) and ^13^C_6_-l-Arg and ^13^C_6_,^15^N_2_-l-Lys in the “heavy” formulation, see [16] for more details. “Light” and “heavy” nuclear extracts were prepared using a commercially available kit (ActiveMotif). The (+)-strand sequences of the wild-type q and mutant Q oligonucleotides were as follows (QTN underscored):

IGF2-q: 5′-Biotin-GATCCTTCGCCTAGGCTCGCAGCGCGGGAGCGA-3′

IGF2-Q: 5′-Biotin-GATCCTTCGCCTAGGCTCACAGCGCGGGAGCGA-3′

The complementary (−)-strands were also synthesized, and the pairs were annealed prior to use. One picomole of ds-q oligonucleotide was mixed with 1.4 mg of heavy nuclear extract and 1 pmol of ds-Q oligonucleotide was mixed with 1.4 mg of light nuclear extract in binding buffer (50 mM Tris [pH 8], 150 mM NaCl, 0.25 mM EDTA, 0.5 mM DTT, 0.1% Tween-20, 0.5 mg/ml BSA, 200 µg/ml poly-dI/dC) in a total volume of 700 µl. Mixtures were incubated for 45 min at room temperature (RT) on a rotator. Ten microliters of streptavidin-coated magnetic beads (Dynal) was added to each tube, and the mixture was further incubated for 30 min at RT on a rotator. Beads were spun for 5 s at 1,000*g* and then captured using a magnetic pull-down system. Beads were washed 3×700 µl in binding buffer without poly-dI/dC and then 4×700 µl in binding buffer without poly-dI/dC or BSA. The supernatant was discarded, and proteins were eluted by boiling in Laemmli buffer (+10 mM DTT). Protein eluates were mixed 1∶1 by volume.

Proteins were separated on an SDS-PAGE gel (4%–12% NuPage), stained with Coomassie Blue, and the entire lane was cut into ∼20 bands. Each band was reduced, alkylated, digested with trypsin according to standard proteomics practices [17], and the resulting peptides were analyzed by LCMS on an Orbitrap (ThermoFisher) mass spectrometer as described [18]. Database searching was performed by Mascot against the REFSEQ database of mouse proteins as of June 2006. SILAC quantification was performed using msQuant [19].

### Expression of Recombinant Protein and Antibody Production

RT-PCR, using poly-A–enriched RNA extracted from C2C12 cells, was used to obtain nucleotides 1–2,943 and 139–2,943 of mouse *Zbed6* transcripts (named *Zbed6a* and *Zbed6b*, respectively; EMBL Bank accession number FM882123); the two constructs begin at the two alternative start codons. These constructs, containing a Kozak sequence for efficient initiation of translation, were cloned into pcDNA3 (Invitrogen) and verified by DNA sequencing.

PCR was used to subclone a fragment encoding the two BED domains, amino acid residues 90–384 of ZBED6a, into pGEX-5X-3 (GE Healthcare). GST and GST-BED1/2 fusion protein was purified from BL21(DE3)pLysS bacteria using gluthathione Sepharose 4B or GSTrap FF columns (GE Healthcare), according to the manufacturer's instructions.

Polyclonal antibody production was performed by Agrisera AB. Shortly, GST-BED1/2 was used to immunize one rabbit. Polyclonal anti-ZBED6 antibodies were affinity purified by first passing serum over a HiTrap NHS-activated HP column (GE Healthcare) coupled with GST, whereafter the flow-through was applied to a column coupled with GST-BED1/2. Anti-ZBED6 antibodies were eluted with 0.2 M glycine (pH 2.5) and dialyzed against 20 mM HEPES (pH 7.4) and 150 mM NaCl.

### EMSA

Nuclear extracts from C2C12 cells were prepared using the NucBuster Protein Extraction kit (Novagen). EMSAs were performed as previously described with minor modifications [2]. The following oligonucleotides were annealed in 1× NEB2 buffer (NEB) q/Q-fwd AGATCCTTCGCCTAGGCTC(G/A)CAGCGCGGGAGCGA and q/Q-rev AGATCTCGCTCCCGCGCTG(C/T)GAGCCTAGGCGAAG. A total of 20 ng of purified GST-BED1/2 protein or 10 µg of C2C12 nuclear extracts were preincubated on ice for 20 min in binding buffer (15 mM Hepes-KOH [pH 7.65] at RT, 30.1 mM KCl, 2 mM MgCl_2_, 0.1 mM EDTA, 0.063% NP-40, 7.5% Glycerol, 1.3 mM dithiothreitol, 2 mM spermidine, 0.1 µg/µl Poly(dI-dC)•Poly(dI-dC)). Competition reactions were supplemented with 4 pmol (100-fold molar excess) unlabelled ds-oligonucleotide. After the addition of 40 fmol end-labeled ^32^P-dCTP ds-oligonucleotide, reactions were incubated at RT for 30 min. In EMSAs including supershift reaction, incubation for 20 min at RT preceded the addition of 2 µl of purified polyclonal anti-ZBED6 antibody (0.13 µg/µl) and incubation then continued for an additional 20 min at RT. Complexes were separated on a 1.5-mm 5% native 29∶1 polyacrylamide gel in 0.5× TBE at 70V for 3–4 h.

### Northern Blot

A mouse multiple-tissue Northern blot panel 4 (MN-MT-1) and a mouse developmental tissue skeletal muscle panel (MN-102-D from Zyagen) was used. Partial *Zbed6* and *Zc3h11a* coding sequences were cloned into a vector and probe template amplified by PCR including either SP6 or T7 sequence from the vector. A purified probe template (200 ng) was used for ^32^P-labeled RNA probe synthesis using the MAXIscript Kit (Ambion). Mouse *ß-actin* (*Actb*) was amplified by PCR from C2C12 cDNA, sequenced, and used as template for ^32^P-labeled DNA probe synthesis using the Amersham Megaprime DNA labeling system (GE Healthcare). Hybridizations were done at 68°C (RNA probe) or 42°C (DNA probe) using the ULTRAhyb buffer (Ambion) followed by washes in 2× SSC+0.1% SDS and 0.1× SSC+0.1% SDS at 68°C (RNA probes) or 60°C (DNA probe). Autoradiographs were exposed for a few hours to several days.

### Immunohistochemistry

Mouse and pig tissue were fixed in 4% paraformaldehyde (PFA) in phosphate buffered saline (PBS) for 2–4 h on ice, cryoprotected in 30% sucrose, followed by embedding in Tissue-Tek O.C.T. compound (A/S Chemi-Teknikk) and cryostat sectioning. Fluorescent immunohistochemistry was performed on cryosections from mouse hind limb muscle postnatal day 3 (p3), mouse brain (p22), and pig muscle (*longissimus dorsi*) (6 mo). Rabbit antibodies against ZC3H11A (A301-525A, Bethyl Laboratories) and ZBED6 were diluted to 0.2 µg/ml in PBS containing 1× blocking reagent (Roche), 0.3% Triton X-100 (Sigma), and incubated at 4°C overnight. In the case of the anti-ZC3H11A antibody, a commercially available blocking peptide (Bethyl Laboratories) was used at 2.0 µg/ml and incubated with the anti-ZC3H11A antibody for 2 h at RT prior to application on tissue. The following day, slides were washed in PBS and incubated with a secondary anti-rabbit IgG antibody conjugated to Alexa 594 (Invitrogen) and DAPI (Sigma) in PBS for 1 h at RT. Slides were washed in PBS and mounted with 2.5% DABCO (Sigma) in glycerol containing 0.1M Tris (pH 8.6). For pig muscle tissue, sections were also incubated with Alexa 488–conjugated α-bungarotoxin (1∶1,000, Invitrogen). Staining was analyzed with a fluorescence microscope (Olympus BX61WI) or a confocal microscope (Zeiss LSM 510 META). Images were adjusted for contrast and brightness in Adobe Photoshop (Adobe).

### Cell Culture

The C2C12 mouse myoblast cell line (ATCC-CLR-1772) was cultured at 37°C in a humidified atmosphere of 5% CO_2_ using DMEM (ATCC-30-2002) supplemented with 10% FBS (Invitrogen) and 1× Antibiotic-Antimycotic solution (Invitrogen). The cultures were split every 2 to 3 d. C2C12 cells were differentiated by growing the cells in differentiation media, DMEM with 2% horse serum.

### GFP-BED1/2 Transfection

BED1/2x and BED1/2y (corresponding to amino acid residues 47–384 and 90–384 of ZBED6, respectively) were cloned into pcDNA3 containing an N-terminal enhanced green fluorescent protein (eGFP) coding sequence. A total of 5×10^4^ cells were plated the day before transfection in 12-well plates. Two µg of DNA of either GFP, GFP-BED1/2x, or GFP-BED1/2y was transfected using 6 µl of Lipofectamine 2000 reagent (Invitrogen) in Opti-MEM (Gibco). Medium was changed to growth medium without antibiotics 4 h posttransfection, and cells were analyzed the following day. Photographs were captured using an epifluorescence-equipped Nikon Eclipse TS100 microscope, a superplan fluor 60× objective, and a Nikon D300 body.

### Immunofluorescence and Confocal Microscopy of C2C12 Cells

Cells were seeded on coverslips, in six-well plates, overnight to be around 50%–70% confluent the following day. The coverslips were fixed by 4% formaldehyde for 10 min at RT in PBS (pH 7.4), followed by permeabilization of the cellular membrane with 0.2% Triton X-100 on ice for 10 min, blocked with 5% FBS for 30 min at RT in PBS, and treated with mixture (2 µg/ml each) of rabbit anti-ZBED6 and mouse anti-nucleophosmin 1 (SIGMA, catalog no. B0556) for 1 h at RT. The cells were then washed four times with PBS to remove unbound antibodies and then treated with mixture (20 µg/ml each) of Alexa Flour 488–labeled goat anti-mouse and Texas Red-labeled goat anti-rabbit secondary antibody for 1h at RT. Cells were washed four times with PBS and mounted with Fluoromount G on an objective slide. The fluorescence was analyzed using LSM 510 confocal microscopy (Zeiss).

### Gene Silencing Using siRNA

A total of 5×10^4^ C2C12 cells or 30%–50% confluent cells were transfected with 50 pmol of negative control siRNAs (Ambion) or ZBED6 siRNAs (Ambion) with 6 µl of Lipofectamine 2000 Reagent (Invitrogen) in 1.5 ml of Opti-MEM I (Invitrogen) per well in 6-well plates. The following pooled Silencer Select siRNA sequences (Ambion) were used to silence *Zbed6* expression in C2C12 cells: duplex 1 sense, 5′-CUUCAACACUUCAACGACAtt-3′; duplex 2 sense, 5′-UGUGGUACAUGCAAUCAAAtt-3′; duplex 3 sense, 5′-GGGCUGUUGCCAACAAAGAtt-3′; the dinucleotide tt (indicated in lower case letters) was added to all oligonucleotides to improve the stability of siRNA after transfection. After 24 h, medium was changed to fresh DMEM with 10% FBS. Biological triplicates were used for each siRNA treatment.

### Luciferase Assay

Silencing was performed 2 d prior to transient transfection with luciferase reporters. Previously described [2] constructs containing the porcine *IGF2* QTN region, the porcine *IGF2* P3 promoter and the firefly luciferase reporter gene (P3, q+P3 and Q+P3) were used. *Zbed6*-silenced C2C12 cells or negative control siRNA treated cells grown in 12-well plates were transfected with a total of 2 µg of DNA and 6 µl of Lipofectamine 2000 Reagent (Invitrogen). One microgram of firefly luciferase construct and 20 ng of Renilla luciferase vector as control (ph-RG, Promega) and empty pcDNA3 vector (Invitrogen) up to 2 µg of DNA were used. Transfections were performed in opti-MEM (Gibco), and medium changed to growth medium (DMEM supplemented with 10% FBS) after 4 h. Cells were harvested 24 h posttransfection, and firefly and Renilla luciferase activities were measured using the Dual-Luciferase Reporter Assay System (Promega) and an Infinite M200 luminometer (Tecan).

### Cell Proliferation Assay

At 48 h post-siRNA transfection, the cells were incubated 3–4 h with medium containing 10% Alamar blue (Invitrogen). The reduction of Alamar blue was measured on a Tecan Sunrise Absorbance Plate Reader (Oxidized/Reduced: 600/570 nm).

### Scratch Wound Assay

At 72 h post-siRNA transfection, cells reached almost confluence, and medium was replaced with fresh DMEM supplemented with 0.1% FBS. A surface wound was created by scraping a pipette tip across the confluent cell monolayer. Twenty-four hours after scraping, the number of cells in the scratch was counted and cells treated with negative control siRNAs and *Zbed6* siRNAs were compared. Statistical analysis was performed using a Student *t*-test.

### RNA Isolation and Real-Time (RT) PCR Analysis

RNA was isolated from tissue samples from six C57BL/6 mice (three males and three females) either by RNeasy mini kit (Qiagen) or acidic phenol extraction as described [20]. RNA from C2C12 cell samples was isolated using the RNeasy Mini Kit (Qiagen), then all samples were subjected to reverse transcription using cDNA high capacity kit (Applied Biosystems). mRNA transcripts were measured by quantitative PCR analysis using TaqMan Gene Expression master mix (Applied Biosystems) on a 7900HT Fast RT-PCR System (Applied Biosystems); probes and primers used are given in Table S2. Data were analyzed with a threshold set in the linear range of amplification, based on a standard curve of serial 10-fold dilutions for each primer set. The *Zbed6* data was normalized to the level of cDNA from two endogenous housekeeping genes (*GAPDH* and *18S rRNA*) and plotted as mean fold change (±s.e.m.). Statistical analysis was performed using a Student *t*-test.

### Immunocytochemical Staining of Cytospins of C2C12 Cells

Cells were trypsinized by 0.25% trypsin EDTA (Invitrogen). After blocking with 10% FBS and washing twice in PBS (Invitrogen), 2 to 3 million cells were resuspended in 1 ml of PBS with 20% FBS and centrifuged as cytospins for 3 min at 800 rpm. The cytospins were dried at RT overnight and then fixed with acetone for 10 min and hybridized with 250 µl of anti-ZBED6 antibody (0.2 µg/ml) for 20 min, followed by staining protocol described by Human Protein Atlas (http://www.proteinatlas.org).

### Chromatin Immunoprecipitation (ChIP) Analysis and Sequencing

Normal ChIP was performed as previously described [21], whereas ChIP sequencing was performed as follows. Chromatin was immunoprecipitated from approximately 10^7^ subconfluent C2C12 cells. Protein-DNA cross-links were made in RT for 10 min with 0.37% formaldehyde, and cells were lysed in RIPA buffer (25 mM Tris-HCl [pH 7.6], 150 mM NaCl, 1% NP-40, 1% sodium deoxycholate, 0.1% SDS). Sonication was done to generate DNA fragments mainly in the 100–300-bp range using a BioRuptor (Diagenode) at highest settings for 30 cycles of 30 s. Incubation with 5 µg of antibody was done overnight, and protein-G agarose beads (Roche) were used to pull down the protein-DNA complexes. After washing and elution, proteins were degraded using proteinase K, and DNA was extracted with phenol-chloroform and precipitated using EtOH with the addition of glycogen. DNA from six replicates was pooled and purified using Qiagen MinElute columns. In order to recover as much as possible of the enriched DNA for sequencing, additional sonication of the ChIP DNA was performed using a Covaris instrument to get optimal fragment sizes. Library construction was done according to the manufacturer's protocol (AB SOLiD v3.0 fragment library) with eight rounds of amplification. Sequencing was done on a quarter of a slide and gave 58 million 50-bp reads. Alignment was done in two steps, first the AB pipeline (mapreads) was used to align full-length reads with up to four mismatches, and subsequently, the remaining reads was truncated to 35 bases and realigned with four mismatches using ZOOM! [22]. This gave 18+6.5 million uniquely placed alignments at 23 million unique positions. Each read was extended to 200 bases and overlap profiles were calculated to identify regions of enrichment. FindPeaks 3.1.92 [23] was used to estimate the false discovery rate (FDR), giving a probability of <0.001 at 15 overlapping fragments. Peaks falling within 100 kb of centromeric gaps or overlapping with Satellite and rRNA repeats were removed to reduce nonrandom false-positive peak calls. This gave 2,499 peaks, which were then associated with the nearest TSS and CpG island (Table S1). We noted that the additional sonication done before sequencing gave larger regions with enrichment compared to what is normally seen in size-selected ChIP-seq dataset, therefore, we additionally filtered the peaks to contain only the highest scoring peak within 5 kb before performing de novo motif finding using BioProspector [24]. We searched for motifs of length 8 bp both in the full list of peaks and in subsets of peaks divided by enrichment and location, and used mouse sequences 1 kb upstream of transcription start sites (TSS) as background. We used the DAVID software for Gene Ontology analysis [25],[26].

### URL

Information on genomes is available at http://www.genome.ucsc.edu.

## Supporting Information

Figure S1 **Alignment of BED domains from the human ZBED family of proteins.** Multiple copies of BED domains in an individual protein are indicated by a hyphen followed by a numeral. Identical amino acid residues (>50%) in a specific position are boxed. Amino acid residues belonging to the same group in a specific position (>50%; hydrophobic, hydrophilic, acid, or basic) are shaded in gray. The four zinc-chelating residues are shown in red. Accessions numbers are NM_004729 (ZBED1), NM_024508 (ZBED2), NM_032367 (ZBED3), NM_014338 (ZBED4), BC047754 (ZBED5), and XM_001726819 (ZBED6).
(0.27 MB TIF)

Figure S2 **Alignment of ZBED6 zinc finger domains from 26 mammalian species.** (A) BED domain 1 and (B) BED domain 2 (amino acid residue numbering as in mouse ZBED6 (FM882123). Accession numbers: pig (*Sus scrofa*; CU655999), human (*Homo sapiens*; XM_001726819), chimpanzee (*Pan troglodytes*; AACZ02012362), orangutan (*Pongo abelii*; ABGA01056271), rhesus monkey (*Macaca mulatta*; AANU01242087), dog (*Canis familiaris*; AAEX02008140), cat (*Felis catus*; AANG01029813), rat (*Rattus norvegicus*; AAHX01075149), cattle (*Bos taurus*; AAFC03020996), northern tree shrew (*Tupaia belangeri*; AAPY01699446), thirteen-lined ground squirrel (Spermophilus tridecemlineatus; AAQQ01279623), common shrew (*Sorex araneus*; AALT01236730), philippine tarsier (*Tarsis syrichta*; ABRT010393906), Ord's kangaroo rat (*Dipodomys ordii*; ABRO01075989), gray mouse lemur (*Microcebus murinus*; ABDC01073199), large flying fox (*Pteropus vampyrus*; ABRP01175854), alpaca (*Lama pacos*; ABRR01303601), horse (*Equus caballus*; AAWR02036736), nine-banded armadillo (*Dasypus novemcinctus*; AAGV020501210), little brown bat (*Myotis lucifugus*; AAPE01620944), bottle-nosed dolphin (*Turiops truncatus*; ABRN01226209), rock hyrax (*Procavia capensis*; ABRQ01352452), guinea pig (*Cavia porcellus*; AAKN02017923), European hedgehog (*Erinaceus europaeus*; AANN01531372), and American pika (*Ochotona princeps*; AAYZ01471625). Initial alignment of the corresponding DNA sequences revealed single nucleotide gaps/insertions in some sequences originating from the Whole-Genome-Shotgun Sequence database at http://www.ncbi.nlm.nih.gov/ (posted date October 24, 2008, 2:23 AM). These positions have been treated as sequence artifacts and have therefore been corrected to give a continuous reading frame before translating to protein sequence. A single nucleotide insertion has been removed from American pika and thirteen-lined ground squirrel. A missing base (N) has been introduced at codon 173 in alpaca and at codon 306 in northern tree shrew. The Gln-residue (Q) at position 131 in rock hyrax is possibly caused by two sequence errors (an insertion of a C followed by a deletion of an A), but remains unedited. Dashes indicate sequence identity to the master sequence (pig). Blanks indicate missing data.
(0.28 MB TIF)

Figure S3 **Differentiation of scrambled and** ***Zbed6*** **-knockdown myoblasts into myotubes on day 6.** C2C12 myoblasts treated with scrambled and *Zbed6* siRNA were submitted to a differentiation medium with 0.1% horse serum in the culture and were followed during 6 d in culture. Three replicates are shown.
(7.02 MB TIF)

Table S1 **ZBED6 binding sites in mouse C2C12 cells identified using ChIP sequencing.** All peaks with at least 15 overlapping reads are listed and sorted according to the number of reads. Dist_CpG, distance to closest CpG island in base pairs; Dist_TSS, distance to transcription start site in base pairs; GeneID, gene name; overlaps, number of overlapping extended reads.
(0.17 MB PDF)

Table S2 **Primers and probes for real-time PCR analysis of** ***Zbed6*** **,** ***Igf2*** **, and** ***Zc3h11a*** **.**
(0.03 MB DOC)

## Abbreviations

ChIP: chromatin immunoprecipitation
EMSA: electrophoretic mobility shift assay
QTL: quantitative trait locus
QTN: quantitative trait nucleotide
RT-PCR: real-time PCR
SILAC: stable isotope labeling of amino acids in culture
siRNA: small interfering RNA
TSS: transcription start site

## References

[1] Jeon J-T, Carlborg Ö, Törnsten A, Giuffra E, Amarger V (1999). A paternally expressed QTL affecting skeletal and cardiac muscle mass in pigs maps to the IGF2 locus.. Nat Genet.

[2] Van Laere A. S, Nguyen M, Braunschweig M, Nezer C, Collette C (2003). A regulatory mutation in IGF2 causes a major QTL effect on muscle growth in the pig.. Nature.

[3] Braunschweig M. H, Van Laere A-S, Buys N, Andersson L, Andersson G (2004). IGF2 antisense transcript expression in porcine postnatal muscle is affected by a quantitative trait nucleotide in intron 3.. Genomics.

[4] Ong S. E, Blagoev B, Kratchmarova I, Kristensen D. B, Steen H (2002). Stable isotope labeling by amino acids in cell culture, SILAC, as a simple and accurate approach to expression proteomics.. Mol Cell Proteomics.

[5] Liang J, Song W, Tromp G, Kolattukudy P. E, Fu M (2008). Genome-wide survey and expression profiling of CCCH-zinc finger family reveals a functional module in macrophage activation.. PLoS One.

[6] Aravind L (2000). The BED finger, a novel DNA-binding domain in chromatin-boundary-element-binding proteins and transposases.. Trends Biochem Sci.

[7] Calvi B. R, Hong T. J, Findley S. D, Gelbart W. M (1991). Evidence for a common evolutionary origin of inverted repeat transposons in Drosophila and plants: hobo, Activator, and Tam3.. Cell.

[8] Grisendi S, Mecucci C, Falini B, Pandolfi P. P (2006). Nucleophosmin and cancer.. Nat Rev Cancer.

[9] Wang Y. H, Chen Y. H, Lu J. H, Tsai H. J (2005). A 23-amino acid motif spanning the basic domain targets zebrafish myogenic regulatory factor myf5 into nucleolus.. DNA Cell Biol.

[10] Mayer C, Grummt I (2005). Cellular stress and nucleolar function.. Cell Cycle.

[11] Feschotte C (2008). Transposable elements and the evolution of regulatory networks.. Nat Rev Genet.

[12] Lander E. S, Linton L. M, Birren B (2001). Initial sequencing and analysis of the human genome.. Nature.

[13] Matsukage A, Hirose F, Yoo M. A, Yamaguchi M (2008). The DRE/DREF transcriptional regulatory system: a master key for cell proliferation.. Biochim Biophys Acta.

[14] Charalambous M, Smith F. M, Bennett W. R, Crew T. E, Mackenzie F (2003). Disruption of the imprinted Grb10 gene leads to disproportionate overgrowth by an Igf2-independent mechanism.. Proc Nat Acad Sci U S A.

[15] Bird A. P (1980). DNA methylation and the frequency of CpG in animal DNA.. Nucl Acids Res.

[16] Ong S. E, Mann M (2006). A practical recipe for stable isotope labeling by amino acids in cell culture (SILAC).. Nat Protoc.

[17] Kinter M, Sherman N. E (2000). Protein sequencing and identification using tandem mass spectrometry.

[18] Jaffe J. D, Keshishian H, Chang B, Addona T. A, Gillette M. A (2008). Accurate inclusion mass screening: a bridge from unbiased discovery to targeted assay development for biomarker verification.. Mol Cell Proteomics.

[19] Andersen J. S, Lam Y. W, Leung A. K, Ong S. E, Lyon C. E (2005). Nucleolar proteome dynamics.. Nature.

[20] (2006). Purification of RNA from cells and tissues by acid phenol-guanidinium thiocyanate-chloroform extraction.. Nat Methods.

[21] Lindroth A. M, Park Y. J, McLean C. M, Dokshin G. A, Persson J. M (2008). Antagonism between DNA and H3K27 methylation at the imprinted Rasgrf1 locus.. PLoS Genet.

[22] Lin H, Zhang Z, Zhang M. Q, Ma B, Li M (2008). LinksZOOM! Zillions of oligos mapped.. Bioinformatics.

[23] Fejes A. P, Robertson G, Bilenky M, Varhol R, Bainbridge M (2008). FindPeaks 3.1: a tool for identifying areas of enrichment from massively parallel short-read sequencing technology.. Bioinformatics.

[24] Liu X. S, Brutlag D. L, Liu J. S (2002). An algorithm for finding protein-DNA binding sites with applications to chromatin-immunoprecipitation microarray experiments.. Nat Biotechnol.

[25] Huang D. W, Sherman B. T, Lempicki R. A (2009). Systematic and integrative analysis of large gene lists using DAVID Bioinformatics Resources.. Nat Protoc.

[26] Dennis G, Sherman B. T, Hosack D. A, Yang J, Gao W (2003). DAVID: Database for Annotation, Visualization, and Integrated Discovery.. Genome Biol.

