# Supplementary figures and images for: ZBED6, a Novel Transcription Factor Derived from a Domesticated DNA Transposon Regulates IGF2 Expression and Muscle Growth
Source: PLoS Biol. 2009 Dec 15;7(12):e1000256. doi: 10.1371/journal.pbio.1000256 (PMC2780926; doi:10.1371/journal.pbio.1000256)

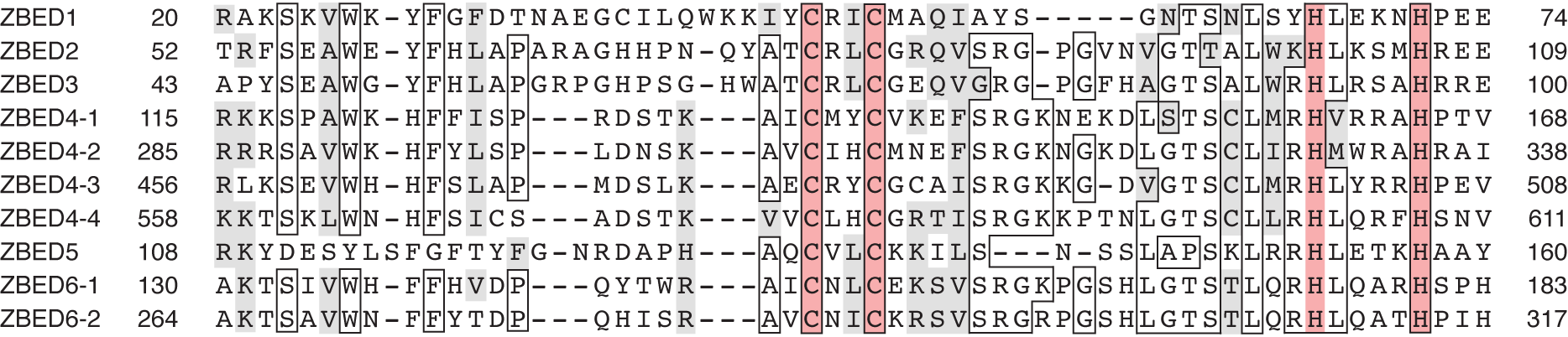

Supplement: Figure S1 — Alignment of BED domains from the human ZBED family of proteins. Multiple copies of BED domains in an individual protein are indicated by a hyphen followed by a numeral. Identical amino acid residues (>50%) in a specific position are boxed. Amino acid residues belonging to the same group in a specific position (>50%; hydrophobic, hydrophilic, acid, or basic) are shaded in gray. The four zinc-chelating residues are shown in red. Accessions numbers are NM_004729 (ZBED1), NM_024508 (ZBED2), NM_032367 (ZBED3), NM_014338 (ZBED4), BC047754 (ZBED5), and XM_001726819 (ZBED6). (0.27 MB TIF) [file pbio.1000256.s001.tif]

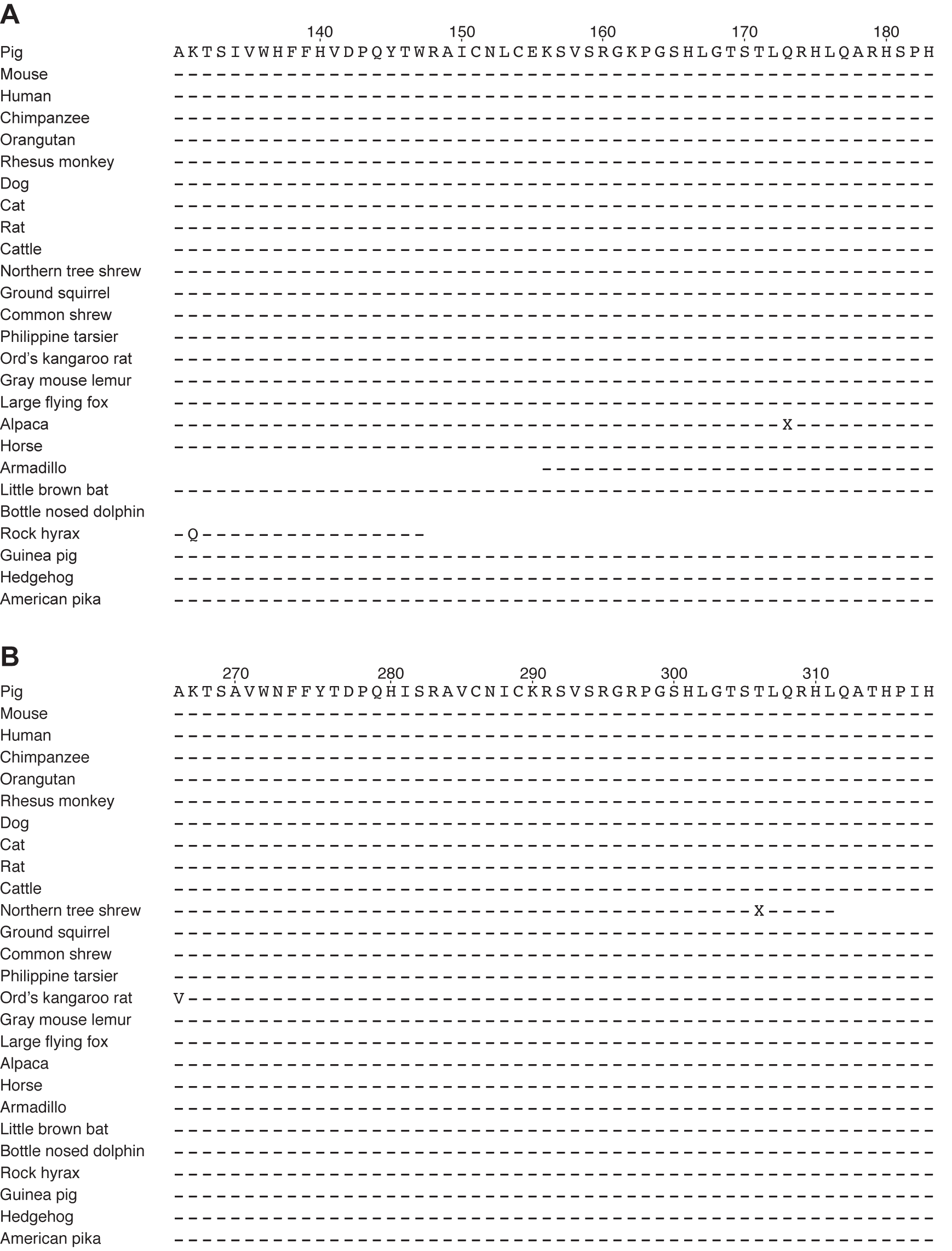

Supplement: Figure S2 — Alignment of ZBED6 zinc finger domains from 26 mammalian species. (A) BED domain 1 and (B) BED domain 2 (amino acid residue numbering as in mouse ZBED6 (FM882123). Accession numbers: pig (Sus scrofa; CU655999), human (Homo sapiens; XM_001726819), chimpanzee (Pan troglodytes; AACZ02012362), orangutan (Pongo abelii; ABGA01056271), rhesus monkey (Macaca mulatta; AANU01242087), dog (Canis familiaris; AAEX02008140), cat (Felis catus; AANG01029813), rat (Rattus norvegicus; AAHX01075149), cattle (Bos taurus; AAFC03020996), northern tree shrew (Tupaia belangeri; AAPY01699446), thirteen-lined ground squirrel (Spermophilus tridecemlineatus; AAQQ01279623), common shrew (Sorex araneus; AALT01236730), philippine tarsier (Tarsis syrichta; ABRT010393906), Ord's kangaroo rat (Dipodomys ordii; ABRO01075989), gray mouse lemur (Microcebus murinus; ABDC01073199), large flying fox (Pteropus vampyrus; ABRP01175854), alpaca (Lama pacos; ABRR01303601), horse (Equus caballus; AAWR02036736), nine-banded armadillo (Dasypus novemcinctus; AAGV020501210), little brown bat (Myotis lucifugus; AAPE01620944), bottle-nosed dolphin (Turiops truncatus; ABRN01226209), rock hyrax (Procavia capensis; ABRQ01352452), guinea pig (Cavia porcellus; AAKN02017923), European hedgehog (Erinaceus europaeus; AANN01531372), and American pika (Ochotona princeps; AAYZ01471625). Initial alignment of the corresponding DNA sequences revealed single nucleotide gaps/insertions in some sequences originating from the Whole-Genome-Shotgun Sequence database at http://www.ncbi.nlm.nih.gov/ (posted date October 24, 2008, 2:23 AM). These positions have been treated as sequence artifacts and have therefore been corrected to give a continuous reading frame before translating to protein sequence. A single nucleotide insertion has been removed from American pika and thirteen-lined ground squirrel. A missing base (N) has been introduced at codon 173 in alpaca and at codon 306 in northern tree shrew. The Gln-residue (Q) at position 131 [file pbio.1000256.s002.tif]

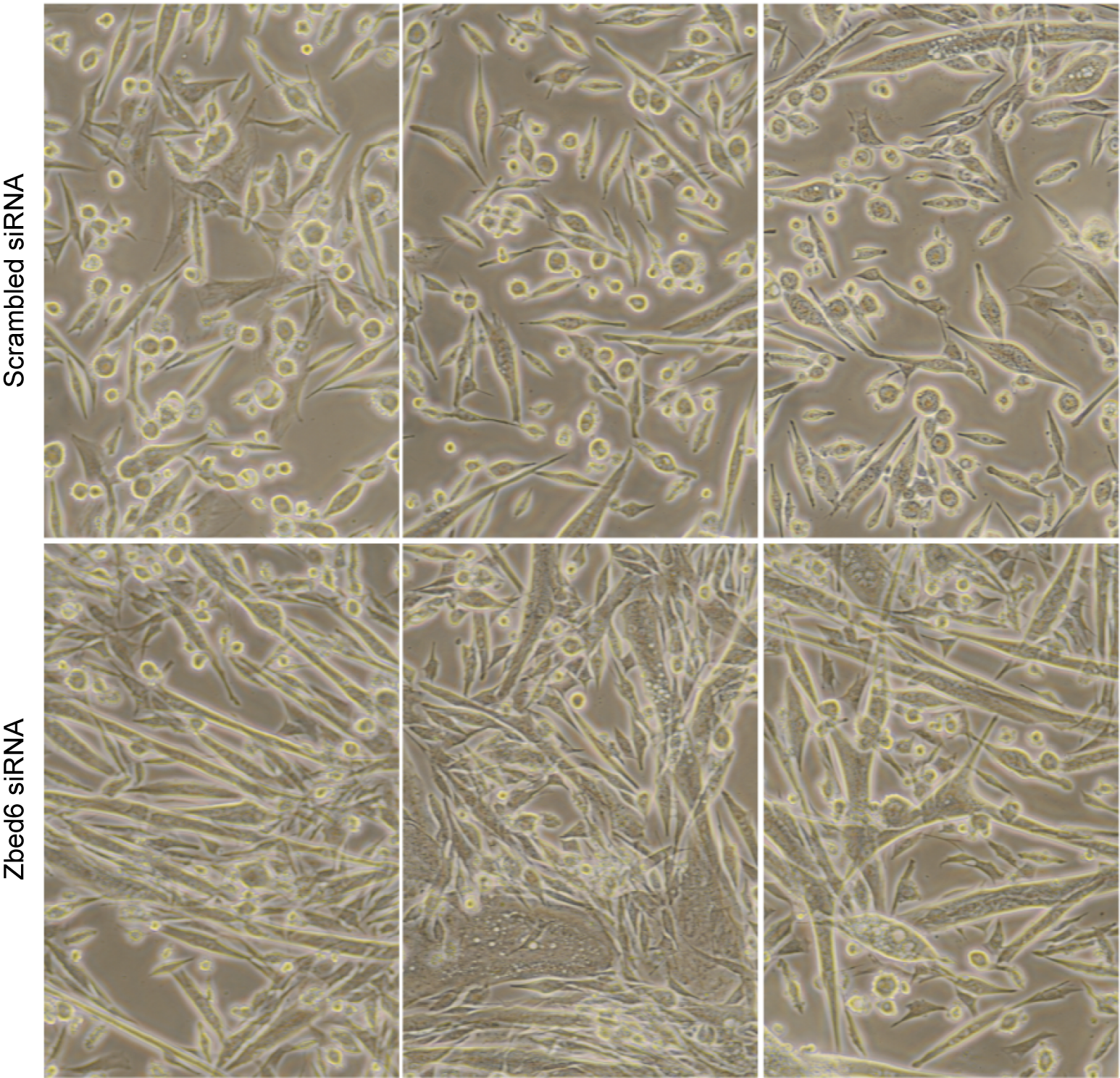

Supplement: Figure S3 — Differentiation of scrambled and Zbed6 -knockdown myoblasts into myotubes on day 6. C2C12 myoblasts treated with scrambled and Zbed6 siRNA were submitted to a differentiation medium with 0.1% horse serum in the culture and were followed during 6 d in culture. Three replicates are shown. (7.02 MB TIF) [file pbio.1000256.s003.tif]
