# Supplementary material for: ZBED6, a Novel Transcription Factor Derived from a Domesticated DNA Transposon Regulates IGF2 Expression and Muscle Growth
Source: PLoS Biol. 2009 Dec 15;7(12):e1000256. doi: 10.1371/journal.pbio.1000256 (PMC2780926; doi:10.1371/journal.pbio.1000256)
Supplement: Table S1 — ZBED6 binding sites in mouse C2C12 cells identified using ChIP sequencing. All peaks with at least 15 overlapping reads are listed and sorted according to the number of reads. Dist_CpG, distance to closest CpG island in base pairs; Dist_TSS, distance to transcription start site in base pairs; GeneID, gene name; overlaps, number of overlapping extended reads. (0.17 MB PDF) [file pbio.1000256.s004.pdf]

Table S1. Zbed6 binding sites in mouse C2C12 cells identifed using ChIP-sequencing. All peaks with at least 15 overlapping reads are listed and sorted according to the number of reads. Overlaps=Number of overlapping extended reads. Dist_CpG=Distance to closest CpG island in base pair. Dist_TSS=Distance to transcription start site in base pair. GeneID= Gene name.

Peak region (+/- 250 BP)	Overlaps	Dist_CpG	Dist_TSS	GeneID
chr17:39982690-39983190	190	0	500000	
chr6:143115180-143115680	166	0	-319	AK164928
chr10:77084530-77085030	151	44	-285	Ube2g2
chr11:102978410-102978910	150	0	1022	Hexim1
chr6:124946270-124946770	140	309	-217	C530028O21Rik
chr11:114944830-114945330	140	7684	-7664	Rab37
chr7:107177800-107178300	138	12637	23102	Chrdl2
chr5:31190040-31190540	137	0	-934	Agbl5
chr2:130123880-130124380	134	2167	2456	Ebf4
chr12:36883680-36884180	134	430	154	Ankmy2
chr7:142927910-142928410	134	19762	-20119	Ki-67
chr17:5621830-5622330	129	500000	500000	
chr18:40467630-40468130	123	500000	49840	Kctd16
chr15:79927990-79928490	123	5927	6477	Syngr1
chrX:96132560-96133060	120	483	-309	Yipf6
chr4:155609440-155609940	120	591	-746	Klhl17
chr15:79301360-79301860	120	14144	-29174	Csnk1e
chr12:88112740-88113240	117	45482	-45590	Angel1
chr18:25213460-25213960	115	500000	-11007	Fhod3
chr15:74917030-74917530	111	500000	25098	Ly6c2
chr5:36009910-36010410	110	11461	12270	Htra3
chr8:112222205-112222705	107	4816	-5292	2400003C14Rik
chr3:89156995-89157495	106	2836	-3314	Adam15
chr12:112667580-112668080	105	0	12175	1200009I06Rik
chr11:3059580-3060080	105	33170	-1892	Sfi1
chr11:3095860-3096360	105	2408	-2645	Sfi1
chr17:26250500-26251000	103	0	489	Tmem8
chr10:59413870-59414370	103	0	397	Ddit4
chr4:41587050-41587550	102	28	34	2310040A07Rik
chr5:31481740-31482240	101	0	526	Gtf3c2
chr9:122930070-122930570	99	0	-123	Tmem42
chr10:61948720-61949220	98	66	375	Vps26a
chr11:3079380-3079880	96	13370	13835	Sfi1
chr14:76186230-76186730	96	0	363	Slc25a30
chr6:147212725-147213225	96	148	-568	Pthlh
chr12:75008170-75008670	95	42	-596	Hif1a
chr5:77787610-77788110	94	47980	48335	Polr2b
chr7:120350400-120350900	94	0	-328	Arntl
chr2:112665580-112666080	92	500000	500000	
chr11:3087170-3087670	91	5580	6045	Sfi1

chr8:13104630-13105130	91	0	462	Pcid2
chr17:36974240-36974740	91	99	405	Trim26
chr7:144506355-144506855	89	0	-478	Ebf3
chr17:8845660-8846160	88	500000	500000	
chr11:3051150-3051650	88	27630	-860	BC025885
chr9:103014360-103014860	87	0	207	Rab6b
chr4:109339520-109340020	87	0	-509	Cdkn2c
chr11:3074245-3074745	87	18505	-16557	Sfi1
chr11:3082350-3082850	86	10400	10865	Sfi1
chr2:181652090-181652590	85	500000	500000	
chr11:102179530-102180030	85	0	629	Ubtf
chr11:18920240-18920740	85	5	-1808	Meis1
chr3:83567820-83568320	85	45	-2172	Sfrp2
chr18:3005580-3006080	83	500000	500000	
chr11:60202360-60202860	81	27632	27896	Atpaf2
chr17:48233980-48234480	81	150	-6903	AK043085
chr8:113782490-113782990	79	0	361	Glg1
chr15:58720040-58720540	79	0	-562	Rnf139
chr11:3098800-3099300	79	3235	-3344	Eif4enif1
chr1:197067670-197068170	77	0	500000	
chr3:69120720-69121220	77	0	131	Ppm1l
chr2:31526060-31526560	75	0	54	Exosc2
chr3:55465600-55466100	74	500000	500000	
chr19:10599770-10600270	74	0	265	5730453I16Rik
chr17:80689350-80689850	74	0	49	Mopt
chr16:62786320-62786820	74	0	-29	Nsun3
chr3:95118830-95119330	74	0	-42	AK144859
chrX:166427080-166427580	74	100	-2460	AK019053
chr9:24955630-24956130	73	0	194	Herpud2
chr13:96145042-96145542	73	0	-2	Aggf1
chr10:127895650-127896150	73	0	-449	Smarcc2
chrX:166432690-166433190	73	773	-8070	AK019053
chr3:8246160-8246660	72	500000	500000	
chr4:102621540-102622040	72	500000	-15329	SGIP1alpha
chr11:18968340-18968840	72	40976	-49908	Meis1
chr2:32612950-32613450	71	175	410	Tor2a
chr9:21961440-21961940	71	10	401	Pigyl
chr5:108797030-108797530	71	315	26	AK165889
chr7:106529730-106530230	71	0	-78	Gdpd5
chr7:149135220-149135720	71	0	-185	Brsk2
chr11:60512865-60513365	71	81	-226	Llgl1
chr14:21894820-21895320	71	0	-303	Adk
chr16:32430450-32430950	71	3	-381	Pcyt1a
chr8:73027440-73027940	70	336	487	CRLM3
chr9:94437810-94438310	70	0	439	1190002N15Rik
chr11:53953810-53954310	69	39187	12866	P4ha2

chr11:74185050-74185550	69	500000	7128	Olfr412
chr10:67739140-67739640	69	357	2083	Arid5b
chr5:149982710-149983210	68	12729	12678	AK082086
chr9:64989480-64989980	68	0	735	Punc
chr19:12576080-12576580	68	41	304	Dtx4
chr8:85689245-85689745	68	0	245	Tbc1d9
chr9:106105340-106105840	68	0	152	Twf2
chr1:183672710-183673210	66	500000	1794	A230079K17Rik
chr6:38586130-38586630	66	0	858	Klrg2
chr17:47896630-47897130	66	21380	588	AK195221
chr13:63674970-63675470	66	180	-8393	Ptch1
chr15:36645520-36646020	65	500000	500000	
chr4:151726315-151726815	65	10774	13806	Chd5
chr15:97826530-97827030	65	5541	8295	Col2a1
chr6:95068205-95068705	65	0	556	Kbtbd8
chr15:85219610-85220110	64	500000	500000	
chr19:45724400-45724900	64	9512	10032	Fbxw4
chr5:67009625-67010125	64	0	34	Apbb2
chr15:88693685-88694185	63	0	1312	Pim3
chr6:39369310-39369810	63	18	214	Mkrn1
chr11:20531880-20532380	62	0	151	Sertad2
chr6:124813950-124814450	62	223	-266	Gpr162
chr7:120513260-120513760	62	10	-658	Btbd10
chr7:140315855-140316355	62	0	-940	Ctbp2
chr14:55615015-55615515	62	18394	-1880	Myh7
chr4:132567525-132568025	61	0	355	Ahdc1
chr17:27194460-27194960	61	185	230	Itpr3
chr11:87172040-87172540	61	0	205	Ppm1e
chr5:119010580-119011080	61	0	103	Med13l
chr16:4419265-4419765	61	121	72	Adcy9
chr16:26105890-26106390	61	126	-271	Leprel1
chr11:49699940-49700440	61	14894	-15146	Rasgef1c
chr9:122565150-122565650	60	500000	500000	
chr11:19958860-19959360	60	37846	500000	
chr17:29237545-29238045	60	2188	2644	Cdkn1a
chr12:76835790-76836290	60	188	606	Sgpp1
chr10:86193560-86194060	59	25100	18301	BC030307
chr11:5873910-5874410	59	1753	4354	Camk2b
chr1:24620400-24620900	59	26634	1000	Atpase6
chr10:11063260-11063760	59	0	268	Epm2a
chr9:66793795-66794295	59	0	121	Rps27l
chr3:55464700-55465200	58	500000	500000	
chr18:3008505-3009005	58	500000	500000	
chr11:114530070-114530570	58	0	464	Rpl38
chr11:105946210-105946710	58	0	245	Map3k3
chr5:45841105-45841605	58	0	40	Qdpr

chr12:85516570-85517070	57	16798	18159	C130039O16Rik
chr18:16966380-16966880	57	0	1124	Cdh2
chr6:86347190-86347690	57	215	-327	Pcyox1
chr13:3372110-3372610	56	11772	500000	
chr9:62566130-62566630	56	40634	40713	Itga11
chr19:61275435-61275935	56	25311	28576	Csf2ra
chr12:85533410-85533910	55	0	1319	C130039O16Rik
chr8:113823480-113823980	55	0	391	Rfwd3
chr9:56775895-56776395	55	0	32	Sh3px3
chr2:29107530-29108030	55	0	-221	mKIAA1857
chr1:69903600-69904100	54	30075	30289	Spag16
chr1:57030355-57030855	54	260	1430	AK046737
chr15:97833620-97834120	54	1155	1205	Col2a1
chr7:29162920-29163420	54	220	1076	Zfp36
chr15:96118050-96118550	54	0	348	1700124K17Rik
chr4:148511115-148511615	54	27	293	Apitd1
chr2:122124615-122125115	54	21	230	Duoxa2
chr17:57129115-57129615	54	141	-366	Clpp
chr7:37474380-37474880	54	5701	-8506	Tshz3
chr5:149983900-149984400	53	11539	11488	AK082086
chr17:46159500-46160000	53	7584	5597	Vegfa
chr4:151552740-151553240	53	71	748	Acot7
chr3:87774980-87775480	53	0	216	Nes
chr15:99055325-99055825	53	281	169	Kcnh3
chr15:99860540-99861040	53	8178	-8372	AI317237
chr19:4333930-4334430	53	28128	-28226	Adrbk1
chr1:183674040-183674540	52	500000	3124	A230079K17Rik
chr15:102752550-102753050	52	178	1239	Hoxc13
chr18:62071165-62071665	52	0	36	Ablim3
chr19:47089975-47090475	52	0	-583	Nt5c2
chr8:87306530-87307030	52	1340	-8513	Nfix
chr2:22444250-22444750	52	33197	-33346	Gad2
chr18:82390320-82390820	51	500000	500000	
chr10:90319330-90319830	51	26934	26993	Anks1b
chr15:85496660-85497160	51	3852	20398	AK041762
chr11:104411150-104411650	51	0	533	Cdc27
chr8:79423955-79424455	51	0	314	AK162930
chr11:78074600-78075100	51	69	-407	2610507B11Rik
chr18:13132070-13132570	50	16	1560	Impact
chrX:154035210-154035710	50	630	1186	Mbtps2
chr2:125331340-125331840	50	0	583	Fbn1
chr3:120965940-120966440	50	33	43	Tmem56
chr3:8245020-8245520	49	500000	500000	
chr3:12640260-12640760	49	500000	500000	
chr1:167166605-167167105	49	0	218	Tiprl
chr1:180459110-180459610	49	0	105	D230039L06Rik

chr16:70314090-70314590	49	0	9	Gbe1
chr5:120120150-120120650	49	0	-277	Tbx3
chr2:113791960-113792460	49	46889	-47092	AK045885
chr2:19192000-19192500	48	500000	500000	
chr2:122507430-122507930	48	20259	20718	Slc30a4
chr12:81360265-81360765	48	0	787	Actn1
chr16:38902520-38903020	48	0	313	Igsf11
chr16:32247180-32247680	48	40	59	Wdr53
chr1:193933140-193933640	48	16758	-16946	Traf5
chr4:130299890-130300390	48	48126	-48311	Sdc3
chr9:60037290-60037790	48	500000	-48599	Thsd4
chr5:148176005-148176505	47	35227	35809	Flt3
chr2:166731930-166732430	47	51	585	Cse1l
chr15:102162405-102162905	47	197	487	Myg1
chr15:79376440-79376940	47	0	480	Ddx17
chr9:71618710-71619210	47	80	448	Cgnl1
chr6:114232760-114233260	47	0	243	Slc6a1
chr2:44965390-44965890	47	0	123	Zeb2
chr11:9018315-9018815	47	31838	-64	Upp1
chr10:67000270-67000770	47	33	-96	Egr2
chr4:154623650-154624150	47	2	-188	2610002J02Rik
chr10:98569305-98569805	47	0	-249	Wdr51b
chr3:87805890-87806390	47	1749	-1989	Bcan
chr17:36368000-36368500	47	0	-17515	AK138323
chr17:5618800-5619300	46	500000	500000	
chr1:188529220-188529720	46	0	397	Tgfb2
chr8:58773340-58773840	46	0	242	Hpgd
chr17:6106330-6106830	46	0	144	AK163710
chr13:76521950-76522450	46	0	-208	Mctp1
chr1:177422700-177423200	46	103	-430	Rgs7
chr15:99500130-99500630	46	309	-768	Accn2
chr3:102906040-102906540	46	28	-25188	Dennd2c
chr11:5081720-5082220	46	29409	-29746	Emid1
chr4:146893080-146893580	46	500000	-34085	OTTMUSG00000010673
chr9:3258615-3259115	45	500000	43552	DQ722852
chrX:109484270-109484770	45	500000	34910	Satl1
chr3:60807275-60807775	45	204	809	P2ry1
chr5:9101155-9101655	45	0	669	4930420K17Rik
chr6:113991110-113991610	45	298	659	Atp2b2
chr10:78995770-78996270	45	97	466	Ppap2c
chr8:122386190-122386690	45	52	236	BC025816
chr1:169279945-169280445	45	67	74	Aldh9a1
chr8:118230870-118231370	45	0	-327	Maf
chr13:59833200-59833700	45	37224	-338	4932411G14Rik
chr13:58230570-58231070	45	0	-2072	AK019388
chr7:138683810-138684310	45	1376	-2416	Hmx3

chrX:166430525-166431025	45	1162	-5905	AK019053
chr10:82816490-82816990	45	6013	-6257	D10Wsu102e
chr3:154086640-154087140	44	500000	500000	
chr16:21771050-21771550	44	22889	16568	Ehhadh
chr1:39633090-39633590	44	30	909	Rnf149
chr1:107887610-107888110	44	0	878	Zcchc2
chr15:99803800-99804300	44	0	840	Larp4
chr6:121134170-121134670	44	0	736	Pex26
chr3:104764775-104765275	44	0	-399	Wnt2b
chr7:119822180-119822680	44	0	-676	Tead1
chr10:61592685-61593185	44	2591	-2902	Neurog3
chr15:12496530-12497030	44	500000	-8669	AK195788
chr15:83443750-83444250	44	18303	-18461	Ttll12
chr10:17690690-17691190	44	22854	-23068	Heca
chr1:167667320-167667820	44	26266	-26340	Creg1
chr1:192365770-192366270	43	500000	500000	
chr6:47601070-47601570	43	0	500000	
chr4:145147600-145148100	43	19143	39015	BC086318
chr19:7248100-7248600	43	7630	32423	Otub1
chr10:21862050-21862550	43	6038	6328	AK053193
chr1:26743675-26744175	43	500000	379	4931408C20Rik
chr10:79379800-79380300	43	122	335	Kiss1r
chr5:135027845-135028345	43	0	208	Clip2
chr8:97508360-97508860	43	500000	108	Gpr56
chr4:102791370-102791870	43	182	-374	Mier1
chr15:88953745-88954245	43	345	-479	Tubgcp6
chr15:74393170-74393670	43	917	-582	Bai1
chr19:42459440-42459940	43	46191	-20970	AK077353
chr15:32075560-32076060	43	500000	-31233	Tas2r119
chr4:152327660-152328160	42	500000	500000	
chr14:108530030-108530530	42	500000	500000	
chr6:100513220-100513720	42	0	36077	AK006738
chr17:25385040-25385540	42	7557	7920	Baiap3
chr9:62190510-62191010	42	0	1080	AK028224
chr10:57206140-57206640	42	0	99	Hsf2
chr7:138553970-138554470	42	0	6	Acadsb
chr4:34011340-34011840	42	0	-16	Cnr1
chr9:61220670-61221170	42	0	-421	Tle3
chr8:47380470-47380970	42	469	-696	Helt
chr11:74642910-74643410	42	241	-1267	Mnt
chr15:28111580-28112080	42	500000	-21690	Dnahc5
chr3:151574710-151575210	41	500000	500000	
chr7:89920070-89920570	41	500000	500000	
chr9:21216230-21216730	41	156	134	Qtrt1
chr19:56471340-56471840	41	0	-28	Casp7
chr6:128493215-128493715	41	500000	-52	BC048546

chr9:107134200-107134700	41	0	-211	Dock3
chr19:6427315-6427815	41	262	-239	Nrxn2
chr15:77137360-77137860	41	0	-253	Rbm9
chr6:32538320-32538820	41	348	-379	Plxna4
chr5:112823170-112823670	41	177	-2188	Asphd2
chr11:55427690-55428190	41	500000	-6649	Glra1
chr15:99861085-99861585	41	7633	-7827	AI317237
chr3:8245605-8246105	40	500000	500000	
chr12:81480960-81481460	40	40143	500000	
chr9:49810550-49811050	40	500000	47818	AK044673
chr12:73475390-73475890	40	33612	34285	Rtn1
chr2:93797435-93797935	40	276	27260	Alkbh3
chr10:126953900-126954400	40	3899	2156	Ndufa4l2
chr5:148536615-148537115	40	0	698	Flt1
chr9:95307050-95307550	40	0	388	Chst2
chr11:75300450-75300950	40	0	379	Prpf8
chr6:52663780-52664280	40	0	289	Tax1bp1
chr3:88306840-88307340	40	0	138	Lmna
chr5:114581360-114581860	40	0	-391	Ung
chr11:76811440-76811940	40	214	-408	Slc6a4
chr14:57504705-57505205	40	0	-534	Zmym2
chr2:48668785-48669285	40	181	-641	Acvr2a
chr15:102528435-102528935	39	5030	23814	Calcoco1
chr4:134506340-134506840	39	19206	19696	Syf2
chr15:98409075-98409575	39	10662	7891	4930415O20Rik
chr7:149850100-149850600	39	58	4753	NR_002855
chr8:124255100-124255600	39	454	888	Jph3
chr11:105927880-105928380	39	404	710	Ccdc44
chr15:75921180-75921680	39	341	625	Nrbp2
chr11:120494180-120494680	39	0	430	Mafg
chr6:115626390-115626890	39	0	12	Raf1
chr2:155299640-155300140	39	40481	-261	Ncoa6
chr11:95685060-95685560	39	1	-503	Phospho1
chr3:88005160-88005660	39	201	-5324	AK141565
chr4:145029650-145030150	39	23544	-24212	NR_002888
chr5:103531760-103532260	39	500000	-44600	Mapk10
chr2:181663680-181664180	38	500000	500000	
chr11:34495155-34495655	38	500000	500000	
chr12:84434535-84435035	38	500000	500000	
chr18:3006085-3006585	38	500000	500000	
chr15:59481350-59481850	38	435	1392	Trib1
chr4:9772060-9772560	38	391	792	Gdf6
chr17:67702905-67703405	38	0	571	Ptprm
chr3:108713850-108714350	38	0	521	Prpf38b
chr12:101203680-101204180	38	0	517	Kcnk13
chr9:119311720-119312220	38	0	352	Acvr2b

chr11:95198400-95198900	38	0	319	5730593F17Rik
chr9:47338470-47338970	38	0	286	Cadm1
chr9:102256690-102257190	38	0	22	Ephb1
chr11:116351990-116352490	38	441	-590	Prpsap1
chr4:118220600-118221100	38	6098	-4518	Tmem125
chr10:127878740-127879240	38	16630	-17359	Smarcc2
chr18:79306080-79306580	38	0	-22653	Setbp1
chr8:126099255-126099755	37	29652	500000	
chr18:10503350-10503850	37	500000	500000	
chr17:88162380-88162880	37	16499	34703	Kcnk12
chr8:86145960-86146460	37	40	13759	Dnajb1
chr17:29246300-29246800	37	10943	11399	Cdkn1a
chr11:98188890-98189390	37	0	1818	Neurod2
chr11:117829180-117829680	37	0	1045	Socs3
chr9:108550540-108551040	37	231	920	Arih2
chr11:6964090-6964590	37	88	849	Adcy1
chr9:74919460-74919960	37	0	698	Myo5a
chr15:99501450-99501950	37	165	552	Accn2
chr2:109731160-109731660	37	0	376	Lin7c
chr15:81958455-81958955	37	0	354	Ccdc134
chr2:26358685-26359185	37	0	221	Notch1
chr8:64238430-64238930	37	0	205	Palld
chr11:76759190-76759690	37	0	181	Blmh
chr3:27610035-27610535	37	202	-57	Fndc3b
chrX:166444750-166445250	37	8734	-20130	AK019053
chr2:112667625-112668125	36	500000	500000	
chr17:48753350-48753850	36	500000	500000	
chr1:62753010-62753510	36	0	3370	Nrp2
chr14:101860170-101860670	36	500000	2019	Tbc1d4
chr15:37721320-37721820	36	0	591	Ncald
chr1:39534750-39535250	36	0	579	Tbc1d8
chr16:90934340-90934840	36	0	467	1110004E09Rik
chr10:41994870-41995370	36	0	283	Foxo3a
chr4:131830300-131830800	36	203	223	Taf12
chr12:114378700-114379200	36	0	193	Crip2
chr15:102033430-102033930	36	191	-87	Csad
chr5:141180630-141181130	36	0	-96	AA881470
chr6:122770090-122770590	36	8	-188	Foxj2
chr9:60709495-60709995	36	500000	-475	Uaca
chr16:9992890-9993390	36	571	-515	Grin2a
chr2:179839610-179840110	36	375	-934	Hrh3
chr2:19373680-19374180	36	5659	-5630	AK053418
chr9:123782010-123782510	36	20980	-11015	Xcr1
chr3:154084885-154085385	35	500000	500000	
chr4:8974365-8974865	35	500000	500000	
chr6:144393020-144393520	35	500000	500000	

chr7:132072320-132072820	35	500000	500000	
chr14:61253435-61253935	35	0	500000	
chr17:13498725-13499225	35	500000	39692	AK036897
chr12:25419555-25420055	35	26238	26658	Rrm2
chr15:100557210-100557710	35	1959	2346	Galnt6
chr14:56382045-56382545	35	580	2031	Ltb4r2
chr19:56798125-56798625	35	0	1514	Adrb1
chr14:31529570-31530070	35	0	936	Sfmbt1
chr19:3685655-3686155	35	0	648	Lrp5
chr5:124991180-124991680	35	0	631	Tmed2
chr5:3802170-3802670	35	0	504	Ankib1
chr3:85775410-85775910	35	243	332	SH3d19
chr8:109207020-109207520	35	0	303	Tmco7
chr16:11202860-11203360	35	0	274	Rsl1d1
chr2:128524750-128525250	35	11766	268	Mertk
chr15:64753420-64753920	35	0	187	Adcy8
chr15:74502570-74503070	35	0	179	Arc
chr11:61575200-61575700	35	0	113	Prpsap2
chr9:68501370-68501870	35	0	12	Rora
chr9:105033875-105034375	35	0	9	Nudt16
chr11:60835290-60835790	35	167	-525	Kcnj12
chr2:127952520-127953020	35	0	-540	Bcl2l11
chr16:8738790-8739290	35	0	-606	Hausp
chr1:39249820-39250320	35	193	-986	Npas2
chr4:146024285-146024785	35	9950	-10389	OTTMUSG00000010105
chr1:190766400-190766900	35	500000	-14496	Ush2a
chr8:49102025-49102525	35	25598	-26371	Wwc2
chr8:126124125-126124625	35	500000	-30739	AK086660
chr9:22843550-22844050	34	500000	500000	
chr12:84435095-84435595	34	500000	500000	
chr16:3350880-3351380	34	500000	500000	
chr3:84237970-84238470	34	45014	45192	6330505N24Rik
chr8:90597500-90598000	34	44854	44755	AK077166
chr5:148034950-148035450	34	33355	34929	Gsx1
chr10:114894580-114895080	34	500000	30470	Ccdc131
chr8:97877010-97877510	34	0	1024	Mmp15
chr5:24031460-24031960	34	103	955	Gbx1
chr10:53098690-53099190	34	118	716	AK039734
chr4:63156225-63156725	34	0	509	Whrn
chr8:24085410-24085910	34	0	345	Ank1
chr8:87823770-87824270	34	9	331	Orc6
chr4:154596065-154596565	34	0	328	Ski
chr5:42234980-42235480	34	0	323	A230054D04Rik
chr17:48060970-48061470	34	0	171	Foxp4
chr6:137117825-137118325	34	0	144	Rerg
chr10:90633970-90634470	34	0	106	Tmpo

chr8:89109480-89109980	34	38253	-5146	Abcc12
chr2:90235060-90235560	34	500000	-5509	Olfr1274
chr2:127664300-127664800	34	6824	-7018	Bub1
chr8:19924570-19925070	34	31640	-7950	AK166824
chr17:23836125-23836625	34	374	-15391	Cldn9
chr2:16604490-16604990	33	500000	500000	
chr5:126278460-126278960	33	500000	500000	
chr15:98886215-98886715	33	233	828	Prph
chr15:87375290-87375790	33	0	812	AW049604
chr11:3190740-3191240	33	0	531	Patz1
chr11:20101860-20102360	33	187	506	Rab1
chrX:96331665-96332165	33	0	447	Efnb1
chr6:7642495-7642995	33	237	436	Asns
chr18:10706040-10706540	33	0	403	Abhd3
chr10:83185790-83186290	33	67	398	AK005183
chr9:98856160-98856660	33	0	331	Foxl2
chr11:53520315-53520815	33	0	255	Rad50
chr1:121733150-121733650	33	0	247	Ptpn4
chr19:47089295-47089795	33	0	97	Nt5c2
chr2:120803120-120803620	33	0	4	Tmem62
chr11:115375440-115375940	33	0	-7	Hn1
chr2:130109935-130110435	33	0	-23	Idh3b
chr10:8238440-8238940	33	107	-68	Ust
chr4:45543140-45543640	33	0	-189	Shb
chr6:91633500-91634000	33	375	-338	Slc6a6
chr8:24084010-24084510	33	644	-1055	Ank1
chr14:35631920-35632420	33	81	-1151	Grid1
chr11:94545370-94545870	33	6726	-6847	Xylt2
chr18:13107580-13108080	33	7365	-7602	Osbpl1a
chr15:12802830-12803330	33	47978	-11662	Rn3
chr19:55239710-55240210	33	500000	-15414	Tectb
chr11:67446605-67447105	33	500000	-19223	Gas7
chr2:181665705-181666205	32	500000	500000	
chr19:58461120-58461620	32	500000	500000	
chrX:99073740-99074240	32	28150	29267	AK142977
chr6:29698655-29699155	32	12365	13403	Smo
chr10:57686520-57687020	32	31322	8652	Dux
chr7:4422900-4423400	32	29526	6700	Eps8l1
chr2:125973405-125973905	32	4003	4064	AK015893
chr9:30750770-30751270	32	91	873	Adamts8
chr10:62800710-62801210	32	0	804	Sirt1
chr17:43153830-43154330	32	0	577	Tnfrsf21
chr3:127539610-127540110	32	128	549	BC002199
chr10:17443480-17443980	32	0	504	Cited2
chr1:129101790-129102290	32	0	478	Mgat5
chr9:77602770-77603270	32	0	404	Gclc

chr4:117587175-117587675	32	0	178	Ipo13
chr19:6334980-6335480	32	0	177	Men1
chr17:69733210-69733710	32	0	143	Zfp161
chr5:92512440-92512940	32	0	70	G3bp2
chr11:4034705-4035205	32	271	-207	Rnf215
chr8:59029430-59029930	32	0	-250	Fbxo8
chr11:59119790-59120290	32	0	-391	Wnt9a
chr15:102805940-102806440	32	5891	-1272	Hoxc9
chr1:173470450-173470950	32	36369	-5277	Itln1
chrX:166438760-166439260	32	2744	-14140	AK019053
chr8:126124725-126125225	32	500000	-30139	AK086660
chr17:30861050-30861550	31	500000	500000	
chr17:30883575-30884075	31	500000	500000	
chr17:48872680-48873180	31	500000	500000	
chr4:146973270-146973770	31	500000	16017	2610305D13Rik
chr19:6428900-6429400	31	159	1346	Nrxn2
chr1:88424290-88424790	31	0	1230	Ptma
chr9:95306260-95306760	31	0	1178	Chst2
chr9:87624765-87625265	31	0	1079	Tbx18
chr15:27954275-27954775	31	0	1077	Trio
chr10:42893210-42893710	31	0	875	Sobp
chr17:71350860-71351360	31	0	762	2900073G15Rik
chr11:60836505-60837005	31	0	690	Kcnj12
chr10:93104300-93104800	31	279	605	Ntn4
chr4:148961005-148961505	31	0	509	Clstn1
chr5:110536160-110536660	31	0	475	Gtpbp6
chr8:120807860-120808360	31	0	456	Cdh13
chr10:110602020-110602520	31	0	413	Osbpl8
chr4:144836165-144836665	31	0	357	Tnfrsf1b
chr15:81416450-81416950	31	0	57	Ep300
chr3:146515121-146515621	31	39042	0	4921517B04Rik
chr8:37520950-37521450	31	0	-11	mKIAA1456
chr11:98656470-98656970	31	0	-109	4121402D02Rik
chr1:134087570-134088070	31	88	-187	Lemd1
chr3:130814960-130815460	31	0	-203	BC051212
chr1:181890330-181890830	31	0	-310	Cdc42bpa
chr15:87374005-87374505	31	189	-473	AW049604
chr9:119488685-119489185	31	469	-802	Scn5a
chr8:126148290-126148790	31	29019	-6574	AK086660
chr13:38095780-38096280	31	9840	-10005	Ssr1
chr3:28137560-28138060	31	23651	-24325	AK039113
chr10:89335220-89335720	30	293	500000	
chr15:85494820-85495320	30	3545	18558	AK041762
chr5:72680485-72680985	30	500000	9453	Atp10d
chr1:61692510-61693010	30	6780	6944	Pard3b
chr2:122457215-122457715	30	0	1104	Spata5l1

chr10:84219625-84220125	30	1861	1083	Rfx4
chr15:74395005-74395505	30	658	1034	Bai1
chr8:123261050-123261550	30	916	1025	Irf8
chr7:117103010-117103510	30	154	675	Rab6ip1
chr4:137418410-137418910	30	131	449	Ece1
chr10:120802345-120802845	30	0	445	AK081796
chr4:132195120-132195620	30	108	410	Eya3
chr5:31357310-31357810	30	0	377	Cad
chr4:129012120-129012620	30	0	243	Rbbp4
chr17:3082110-3082610	30	1965	211	AK185737
chr9:34296190-34296690	30	14	152	Kirrel3
chr10:10277635-10278135	30	0	119	Rab32
chr1:9737485-9737985	30	0	7	AK006607
chr3:143865340-143865840	30	184	-295	Lmo4
chr17:56145050-56145550	30	3193	-310	Stap2
chr11:7114175-7114675	30	312	-500	Igfbp3
chr5:24033120-24033620	30	283	-705	Gbx1
chr5:120955060-120955560	30	1582	-1684	1300012G16Rik
chrX:96328075-96328575	30	3125	-3143	Efnb1
chr11:114942410-114942910	30	10104	-10084	Rab37
chr2:118317260-118317760	30	11934	-12079	Srp14
chr8:126142770-126143270	30	34539	-12094	AK086660
chr11:6636970-6637470	30	500000	-17181	AK076664
chrX:166442220-166442720	30	6204	-17600	AK019053
chr12:106674140-106674640	30	500000	-17675	AK081193
chr4:146902240-146902740	30	500000	-24925	OTTMUSG00000010673
chr15:79298065-79298565	30	17439	-25879	Csnk1e
chr9:96559060-96559560	30	27317	-27356	gap1m
chr14:26619700-26620200	30	31090	-41705	Anxa11
chr3:78707805-78708305	29	500000	500000	
chr11:8461910-8462410	29	500000	500000	
chr5:33071400-33071900	29	500000	13231	Pisd
chr15:85488410-85488910	29	2531	12148	AK041762
chr5:114626030-114626530	29	44633	10754	Acacb
chr17:56641870-56642370	29	8601	9785	Znrf4
chr16:91270790-91271290	29	0	1027	Olig1
chr14:117324890-117325390	29	0	604	Gpc6
chr3:120965030-120965530	29	145	526	Tmem56
chr7:36119610-36120110	29	0	518	Rhpn2
chr5:53133060-53133560	29	76	498	Pi4k2b
chr5:20560870-20561370	29	0	494	Ptpn12
chr2:181405730-181406230	29	0	364	Sox18
chr6:85402070-85402570	29	42	254	Cct7
chr13:114408270-114408770	29	0	245	Snag1
chr15:101097260-101097760	29	4	218	Nr4a1
chr5:64359680-64360180	29	0	205	Rell1

chr6:134742200-134742700	29	0	195	Dusp16
chr2:130985380-130985880	29	34	125	1700037H04Rik
chr9:58336155-58336655	29	0	-4	6030419C18Rik
chr6:38304460-38304960	29	0	-108	Zc3hav1
chr11:60033970-60034470	29	79	-115	Srebf1
chr8:12395755-12396255	29	0	-347	Sox1
chr6:113992135-113992635	29	461	-366	PMCA2
chr5:116182505-116183005	29	0	-1188	Ccdc64
chr17:15055600-15056100	29	24088	-9327	Wdr27
chr9:75279045-75279545	29	9826	-10035	Leo1
chr4:3010650-3011150	28	500000	500000	
chr15:86490950-86491450	28	500000	500000	
chr14:63028010-63028510	28	500000	46865	Gucy1b2
chr7:51871460-51871960	28	24532	25455	Kcnc3
chr1:107700860-107701360	28	500000	23811	Tnfrsf11a
chr8:9957220-9957720	28	18650	18852	Lig4
chr3:129016580-129017080	28	500000	18836	Enpep
chr15:83726200-83726700	28	500000	5319	mKIAA1672
chr15:78675690-78676190	28	1981	2864	Cdc42ep1
chrX:7499480-7499980	28	0	2783	Pcsk1n
chr7:51037520-51038020	28	14962	1347	Klk10
chr4:148179420-148179920	28	0	1170	Casz1
chr1:179373410-179373910	28	754	1123	Zfp238
chr13:31651065-31651565	28	0	1010	Foxq1
chr8:94324015-94324515	28	0	1007	Irx3
chr8:23970680-23971180	28	0	920	Myst3
chr5:21849465-21849965	28	156	807	Reln
chr11:115127600-115128100	28	71	706	Grin2c
chr7:4452485-4452985	28	0	546	Ppp1r12c
chr8:86534595-86535095	28	187	495	AK008705
chr9:44127630-44128130	28	0	484	Tmem24
chr3:32716700-32717200	28	69	403	Usp13
chrX:8469660-8470160	28	0	403	B630019K06Rik
chr17:71947440-71947940	28	0	395	AK045842
chr17:3115150-3115650	28	0	295	Rbm16
chr17:45692635-45693135	28	0	221	Nfkbie
chr4:59594380-59594880	28	0	168	Hsdl2
chr14:55304780-55305280	28	36	-22	1700123O20Rik
chr3:85691440-85691940	28	0	-376	Glt28d2
chr5:109122630-109123130	28	83	-640	Fgfrl1
chr3:31106690-31107190	27	1046	500000	
chr8:35287720-35288220	27	500000	500000	
chr8:94598190-94598690	27	500000	500000	
chr16:45352975-45353475	27	500000	500000	
chrX:14316910-14317410	27	500000	500000	
chr6:30124235-30124735	27	58	37915	DQ708924

chr18:10846780-10847280	27	500000	33051	AK079855
chr8:81749510-81750010	27	500000	22708	1700011L22Rik
chr4:8640000-8640500	27	20866	21739	Chd7
chr8:126060110-126060610	27	9241	17626	Gas8
chr8:128285335-128285835	27	27172	12442	Pcnxl2
chr3:36109360-36109860	27	500000	11586	Gpr103
chr12:81857890-81858390	27	261	3533	Gm1568
chr10:79958680-79959180	27	4	1280	Onecut3
chr5:128096495-128096995	27	301	1016	Slc15a4
chr1:168932360-168932860	27	616	1005	C030014K22Rik
chr11:59103140-59103640	27	0	863	Wnt3a
chr9:62995810-62996310	27	416	707	Lbxcor1
chr4:136833945-136834445	27	154	646	Wnt4
chr3:35653415-35653915	27	22	606	Atp11b
chr3:7503650-7504150	27	155	475	3110050N22Rik
chr10:84647080-84647580	27	0	468	Cry1
chr1:93147280-93147780	27	100	388	Ube2f
chr5:116180940-116181440	27	0	377	Ccdc64
chr15:84685925-84686425	27	0	303	Phf21b
chr10:43299020-43299520	27	0	296	Cd24a
chr1:167693930-167694430	27	0	270	Creg1
chr5:35620980-35621480	27	248	16	Adra2c
chr3:145421410-145421910	27	0	-30	Ddah1
chr7:146374210-146374710	27	0	-89	Stk32c
chr15:72376550-72377050	27	0	-92	Kcnk9
chr17:88373830-88374330	27	0	-343	Msh6
chr10:128241995-128242495	27	0	-390	Dnajc14
chr19:4626140-4626640	27	266	-774	Face-2
chr10:41196990-41197490	27	12611	-973	Mical1
chrX:148603060-148603560	27	23640	-10241	Iqsec2
chr13:38097120-38097620	27	11180	-11345	Ssr1
chr17:43742370-43742870	27	24048	-13240	AK006933
chr12:85082845-85083345	27	29597	-21515	Papln
chr4:126675350-126675850	27	22955	-22978	Sfpq
chr11:61221330-61221830	27	0	-30015	RP23-451A6
chr2:22445560-22446060	27	31887	-32036	Gad2
chr10:9907100-9907600	27	500000	-47441	AK016412
chr1:77714540-77715040	26	500000	500000	
chr4:79650410-79650910	26	500000	500000	
chr4:80901350-80901850	26	500000	500000	
chr4:150818610-150819110	26	41083	500000	
chr5:117250785-117251285	26	500000	500000	
chr5:151643020-151643520	26	500000	500000	
chr9:100296070-100296570	26	500000	500000	
chr11:8462855-8463355	26	500000	500000	
chr12:12595550-12596050	26	500000	500000	

chr17:5625260-5625760	26	500000	500000	
chrX:166584175-166584675	26	500000	500000	
chr10:24842900-24843400	26	500000	47889	AK076839
chr7:75142380-75142880	26	44363	45488	Igf1r
chr10:61867895-61868395	26	44031	17059	Hkdc1
chr10:60148710-60149210	26	0	10277	Cdh23
chr16:17832835-17833335	26	457	5193	Car15
chr1:173494200-173494700	26	500000	5127	Cd244
chr6:56322580-56323080	26	500000	3132	AK076685
chr11:107774580-107775080	26	500000	1538	Cacng5
chr6:17644630-17645130	26	0	1475	St7
chr11:72019710-72020210	26	254	972	mKIAA0753
chr5:123349245-123349745	26	8	863	Fbxl10
chr1:181732710-181733210	26	97	830	Ahctf1
chr6:86145975-86146475	26	298	816	Tgfa
chr9:60710565-60711065	26	500000	595	Uaca
chr15:85407660-85408160	26	0	589	Wnt7b
chr11:115024990-115025490	26	0	586	Slc9a3r1
chr4:107107640-107108140	26	0	567	Glis1
chr9:83698940-83699440	26	185	516	Elovl4
chr11:101031850-101032350	26	0	515	Plekhh3
chr8:108693070-108693570	26	253	500	Slc7a6
chr1:16096210-16096710	26	88	498	Rdh10
chr2:131970095-131970595	26	0	498	Slc23a2
chr12:73336975-73337475	26	229	485	Rtn1
chr10:127814390-127814890	26	44	461	Ankrd52
chr4:132324440-132324940	26	0	416	Rpa2
chr13:14047085-14047585	26	0	395	B3galnt2
chr13:55546940-55547440	26	55	369	Grk6
chr10:61595795-61596295	26	251	208	Neurog3
chr5:123794395-123794895	26	0	103	Bcl7a
chr1:145586170-145586670	26	97	-58	Glrx2
chr15:81688580-81689080	26	0	-75	Tob2
chr8:74405600-74406100	26	47	-116	Zfp709
chr19:30104530-30105030	26	0	-222	Uhrf2
chr8:19984445-19984945	26	35094	-321	AK081879
chr1:13364255-13364755	26	0	-342	Ncoa2
chr15:39775435-39775935	26	0	-383	Lrp12
chr8:70586430-70586930	26	84	-496	Psd3
chr7:130513695-130514195	26	0	-548	mNadrin1
chr16:17069465-17069965	26	527	-688	Ypel1
chr18:23198125-23198625	26	0	-1219	Nol4
chr2:179798330-179798830	26	6646	-6717	Gtpbp5
chr2:77128210-77128710	26	9490	-9812	Sestd1
chrX:166434890-166435390	26	0	-10270	AK019053
chrX:11667400-11667900	26	0	-10471	Bcor

chr4:145038610-145039110	26	14584	-15252	NR_002888
chr4:146797770-146798270	26	16364	-24233	AK019745
chr2:168632820-168633320	26	40439	-40370	Sall4
chr1:91147525-91148025	25	500000	500000	
chr6:47596920-47597420	25	4010	500000	
chr6:119057770-119058270	25	0	500000	
chr15:66948270-66948770	25	500000	500000	
chr16:34440660-34441160	25	500000	500000	
chr16:93469670-93470170	25	19904	500000	
chr17:11105150-11105650	25	500000	500000	
chr18:72913780-72914280	25	500000	500000	
chr19:58000825-58001325	25	500000	500000	
chr17:89155570-89156070	25	500000	35495	Lhcgr
chr16:98244050-98244550	25	500000	25812	BC056174
chr4:134505610-134506110	25	18476	18966	Syf2
chr10:80446050-80446550	25	2156	17809	Gng7
chr2:6287420-6287920	25	43080	13889	Usp6nl
chr15:82043310-82043810	25	171	11205	Tnfrsf13c
chr16:18137810-18138310	25	9697	10262	Rtn4r
chr17:72945395-72945895	25	7135	8001	Alk
chr7:107860795-107861295	25	5474	5830	B930006L02Rik
chr5:35622700-35623200	25	0	1736	Adra2c
chr17:13745600-13746100	25	500000	1107	BC068229
chr2:180510360-180510860	25	142	994	Bhlhb4
chr15:43001260-43001760	25	274	853	Rspo2
chr11:50417310-50417810	25	521	822	Adamts2
chr2:32007050-32007550	25	136	624	mKIAA0515
chr6:113574340-113574840	25	51	576	Vhlh
chr15:85167090-85167590	25	12	530	Atxn10
chr3:152009680-152010180	25	0	486	Usp33
chr16:4559040-4559540	25	0	429	Tcfap4
chr9:56842950-56843450	25	0	426	Ptpn9
chr9:48643430-48643930	25	0	369	Zbtb16
chr9:69988760-69989260	25	0	353	6430514L14Rik
chr5:31350430-31350930	25	0	254	Slc5a6
chr2:164312230-164312730	25	0	104	Dbndd2
chr17:63230330-63230830	25	0	85	Efna5
chr17:35804210-35804710	25	0	76	Vars2
chr15:100299320-100299820	25	0	46	Letmd1
chrX:133654760-133655260	25	1190	-146	Esx1
chr14:76814360-76814860	25	0	-157	mKIAA1994
chr7:52409180-52409680	25	0	-242	Pih1d1
chr10:7675950-7676450	25	0	-280	Map3k7ip2
chr3:127202080-127202580	25	139	-298	Ank2
chr1:59820690-59821190	25	0	-540	Bmpr2
chr5:15185580-15186080	25	500000	-697	NR_001585

chr13:46061070-46061570	25	0	-976	Atxn1
chr5:112823950-112824450	25	149	-2968	Asphd2
chr11:114949475-114949975	25	3039	-3019	Rab37
chr4:148909770-148910270	25	17327	-9205	Ctnnbip1
chr16:91534665-91535165	25	12237	-12423	Ifngr2
chr3:40405700-40406200	25	24481	-24257	Pdzd6
chr18:33411970-33412470	25	38455	-38751	Stard4
chr2:28848985-28849485	24	500000	500000	
chr2:160076425-160076925	24	500000	500000	
chr4:53335640-53336140	24	500000	500000	
chr7:140387280-140387780	24	500000	500000	
chr12:98299705-98300205	24	500000	500000	
chr13:111251080-111251580	24	500000	500000	
chr14:48959020-48959520	24	500000	500000	
chr15:7295450-7295950	24	500000	500000	
chr16:96785655-96786155	24	500000	500000	
chrX:11583705-11584205	24	0	500000	
chr14:34063370-34063870	24	500000	33612	Arhgap22
chr10:90321120-90321620	24	28724	28783	Anks1b
chr9:70331280-70331780	24	19343	19630	Rnf111
chr17:29248130-29248630	24	12773	13229	Cdkn1a
chr12:50501760-50502260	24	270	10365	AK043991
chr8:12662875-12663375	24	8647	8974	Tubgcp3
chr4:145743250-145743750	24	20213	5508	LOC433791
chr5:122968760-122969260	24	16253	4982	AK006664
chr1:90137620-90138120	24	35496	3188	AK133186
chr5:140386040-140386540	24	1068	2394	A930017N06Rik
chr11:63734300-63734800	24	0	1235	Hs3st3b1
chr5:14515690-14516190	24	388	1023	Pclo
chr4:154734340-154734840	24	496	909	Prkcz
chr14:48741195-48741695	24	46	902	Peli2
chr19:6117500-6118000	24	108	835	Sac3d1
chr8:11728740-11729240	24	155	822	Arhgef7
chr8:109460150-109460650	24	0	751	Sntb2
chr9:51975680-51976180	24	59	746	mKIAA1726
chr1:36995430-36995930	24	6	691	Tmem131
chr16:26104950-26105450	24	74	669	Leprel1
chr10:25080075-25080575	24	0	664	Epb4
chr8:89408245-89408745	24	3	643	BC004022
chr16:75593500-75594000	24	306	615	Rbm11
chr2:158436830-158437330	24	0	587	Slc32a1
chr13:74147260-74147760	24	0	585	Tppp
chr8:8659970-8660470	24	0	552	Efnb2
chr11:32121435-32121935	24	0	552	Rhbdf1
chr15:76779215-76779715	24	38	492	1110038F14Rik
chr6:94233175-94233675	24	0	472	Magi1

chr7:140829560-140830060	24	0	422	2700050L05Rik
chr10:77875120-77875620	24	4	417	Rrp1
chr11:4087000-4087500	24	45	415	Tbc1d10a
chr5:110714525-110715025	24	182	411	Pxmp2
chr4:137951810-137952310	24	0	382	LOC625638
chr12:87087610-87088110	24	160	372	Flvcr2
chr11:100258440-100258940	24	0	362	Jup
chrX:33868455-33868955	24	0	302	Lonrf3
chr7:28459670-28460170	24	261	241	C030039L03Rik
chr12:117316225-117316725	24	22	210	Vipr2
chr17:35119030-35119530	24	0	177	SmX5
chr2:71205200-71205700	24	125	160	Slc25a12
chr3:156224340-156224840	24	0	119	AK016265
chr16:56717120-56717620	24	0	96	Tfg
chr8:63111510-63112010	24	0	85	Mfap3l
chr3:108829870-108830370	24	0	70	Fam102b
chr2:144993665-144994165	24	0	67	Slc24a3
chr12:29334320-29334820	24	0	58	Rnh1
chr5:104183390-104183890	24	39	49	Aff1
chr7:87439180-87439680	24	0	28	Prc1
chr7:105508445-105508945	24	616	-107	Tsku
chr1:36614260-36614760	24	0	-109	Sema4c
chr3:27081560-27082060	24	0	-115	Aadacl1
chr17:51972430-51972930	24	111	-156	Satb1
chr16:33056790-33057290	24	0	-181	Rpl35a
chr2:170321400-170321900	24	0	-277	Pfdn4
chr13:60278780-60279280	24	78	-305	Gas1
chr11:88017300-88017800	24	112	-369	Mrps23
chr19:53404300-53404800	24	27	-385	Mxi1
chr9:72379370-72379870	24	0	-426	Rfxdc2
chr13:112280310-112280810	24	0	-514	Gpbp1
chr9:58335550-58336050	24	0	-609	6030419C18Rik
chr17:26087530-26088030	24	726	-636	D630044L22Rik
chr3:89154490-89154990	24	331	-809	Adam15
chr8:124253375-124253875	24	101	-837	Jph3
chr4:34010415-34010915	24	0	-941	Cnr1
chr16:22162120-22162620	24	0	-963	Igf2bp2
chr15:39028495-39028995	24	0	-1132	Rims2
chr15:80936360-80936860	24	500000	-1162	Mkl1
chr14:57504040-57504540	24	4	-1199	Zmym2
chr18:24625510-24626010	24	0	-3611	2700062C07Rik
chr8:98051580-98052080	24	500000	-6169	Ccdc113
chr6:81980020-81980520	24	11450	-11396	Tmem166
chr10:88259525-88260025	24	29451	-22060	DRIM
chr4:146900235-146900735	24	500000	-26930	OTTMUSG00000010673
chr4:127637930-127638430	24	10603	-27107	AK046385

chr18:32136790-32137290	24	38011	-27751	Gpr17
chr11:55162650-55163150	24	500000	-37142	Fat2
chr1:149616700-149617200	23	500000	500000	
chr2:28631630-28632130	23	500000	500000	
chr3:130062400-130062900	23	500000	500000	
chr5:7645545-7646045	23	500000	500000	
chr9:118163830-118164330	23	500000	500000	
chr11:111471320-111471820	23	500000	500000	
chr12:26104460-26104960	23	500000	500000	
chr13:3372645-3373145	23	12307	500000	
chr17:86186260-86186760	23	12902	500000	
chr18:77495475-77495975	23	2181	500000	
chr10:24843790-24844290	23	500000	46999	AK076839
chr4:99811530-99812030	23	42750	43385	Ror1
chr14:70283285-70283785	23	500000	43309	1700081D17Rik
chr13:99160540-99161040	23	290	36591	Foxd1
chr10:86723840-86724340	23	500000	30586	AK016129
chr8:73956760-73957260	23	26690	27096	Ankrd41
chr7:25964230-25964730	23	0	27005	AK142847
chr8:34821065-34821565	23	20936	20970	AK018937
chr4:135737345-135737845	23	8463	9287	E2f2
chr8:91150600-91151100	23	147	9176	9130017C17Rik
chr2:33789225-33789725	23	32505	8194	AK162388
chr13:60879560-60880060	23	500000	7899	BC051665
chr11:5870580-5871080	23	1335	7684	Camk2b
chr13:95652360-95652860	23	125	7029	Otp
chr15:99414460-99414960	23	41	5208	Aqp2
chr19:44836555-44837055	23	2995	4922	Pax2
chr17:13744560-13745060	23	500000	2147	BC068229
chr1:121316770-121317270	23	0	1804	Inhbb
chr15:60654575-60655075	23	0	1689	BC020326
chr17:25391335-25391835	23	1262	1625	Baiap3
chr10:80641205-80641705	23	1656	1055	Eef2
chr2:120556180-120556680	23	124	1048	Cdan1
chr17:35371580-35372080	23	80	929	Nfkbil1
chr15:101631600-101632100	23	500000	912	Krt73
chr10:79180010-79180510	23	0	882	Hcn2
chr2:130866410-130866910	23	120	833	Gfra4
chr15:58912470-58912970	23	112	823	Mtss1
chr15:26239345-26239845	23	158	793	March11
chr6:120413760-120414260	23	159	759	Il17ra
chr9:20836950-20837450	23	472	667	Icam5
chr5:100947840-100948340	23	145	613	Cops4
chr11:120534700-120535200	23	100	599	Aspscr1
chr4:84191560-84192060	23	182	498	Bnc2
chr11:94981680-94982180	23	0	482	Dlx3

chr2:5872740-5873240	23	0	476	Upf2
chr2:163575315-163575815	23	0	347	Ada
chr16:94085530-94086030	23	0	276	Sim2
chr1:167123925-167124425	23	0	275	Sft2d2
chr7:80932795-80933295	23	47148	144	DQ718309
chr6:87680735-87681235	23	0	123	AB041550
chr18:24812005-24812505	23	0	64	Mocos
chr11:97560405-97560905	23	0	-4	Pcgf2
chr1:168309170-168309670	23	135	-10	Tada1l
chr8:67171650-67172150	23	0	-64	Cpe
chr11:84632950-84633450	23	63	-184	Mrm1
chr13:120276350-120276850	23	179	-245	EG633640
chr9:75473000-75473500	23	13	-288	Lysmd2
chr10:128181150-128181650	23	189	-289	Dgka
chr16:32644390-32644890	23	0	-306	Tnk2
chr1:60100090-60100590	23	0	-313	Ica1l
chr5:23930570-23931070	23	80	-316	Slc4a2
chr11:5965900-5966400	23	63	-400	Camk2b
chr13:110050330-110050830	23	500000	-425	Pde4d
chr5:120881190-120881690	23	380	-469	Lhx5
chr15:102125925-102126425	23	395	-580	Espl1
chr6:28782250-28782750	23	0	-754	Lrrc4
chr5:21851050-21851550	23	105	-778	Reln
chr9:58670230-58670730	23	0	-838	Hcn4
chr5:112821875-112822375	23	1472	-893	Asphd2
chr15:72377490-72377990	23	0	-1032	Kcnk9
chr10:58275530-58276030	23	0	-1070	Sh3md4
chr17:35356365-35356865	23	15295	-1163	AK080258
chr2:172934880-172935380	23	500000	-1205	AK041254
chr3:107263390-107263890	23	364	-1825	Kcnc4
chr14:70758820-70759320	23	7684	-7840	fad123
chr1:173478250-173478750	23	44169	-10823	Cd244
chrX:166440230-166440730	23	4214	-15610	AK019053
chr5:38234150-38234650	23	15608	-18577	Msx1
chr19:42091370-42091870	23	34658	-18907	Ankrd2
chr4:145018520-145019020	23	34674	-35342	NR_002888
chr4:145158100-145158600	23	8643	-48670	OTTMUSG00000010173
chr4:71686720-71687220	22	500000	500000	
chr6:4112440-4112940	22	500000	500000	
chr11:81036110-81036610	22	500000	500000	
chr11:118431715-118432215	22	242	500000	
chr14:10100070-10100570	22	500000	500000	
chr17:86301610-86302110	22	500000	500000	
chr7:85849110-85849610	22	500000	33537	AK042016
chr4:45713590-45714090	22	500000	17380	Gm829
chr4:146972415-146972915	22	500000	16872	2610305D13Rik

chr1:173448340-173448840	22	14259	14982	Refbp2
chr8:19976060-19976560	22	43479	8064	AK081879
chr5:135817720-135818220	22	7751	7774	Fkbp6
chr1:90114610-90115110	22	12486	6327	Ugt1a1
chr18:56597050-56597550	22	5253	5528	Gramd3
chr10:4526740-4527240	22	3936	4393	Mtrf1l
chr1:173492970-173493470	22	500000	3897	Cd244
chr18:69755920-69756420	22	500000	3091	Tcf4
chr5:35623770-35624270	22	324	2806	Adra2c
chr14:67644960-67645460	22	17212	2656	Ppp2r2a
chr15:7763230-7763730	22	0	2470	Gdnf
chr15:74348570-74349070	22	0	2195	Bai1
chr12:111519135-111519635	22	1257	1946	Dio3
chr17:26976315-26976815	22	0	1944	Nkx2-5
chr6:31166655-31167155	22	0	1527	BC145649
chr12:15821925-15822425	22	0	1415	Trib2
chr1:90109650-90110150	22	7526	1367	Ugt1a1
chr19:42246035-42246535	22	884	1175	Zfyve27
chr15:85862840-85863340	22	0	1116	Celsr1
chr11:53165190-53165690	22	644	1106	Aff4
chr18:25911380-25911880	22	0	1027	Brunol4
chr10:60293125-60293625	22	0	953	Unc5b
chr14:35633990-35634490	22	425	919	Grid1
chr11:49839490-49839990	22	484	908	Rnf130
chr10:77364160-77364660	22	422	844	Lrrc3
chr1:193006160-193006660	22	0	801	Atf3
chr15:78259565-78260065	22	446	758	Kctd17
chr8:26629620-26630120	22	0	627	Fgfr1
chr1:38683225-38683725	22	163	613	Aff3
chr4:45838840-45839340	22	139	608	Igfbpl1
chr11:4493960-4494460	22	5	607	Mtmr3
chr9:57788265-57788765	22	0	574	Sema7a
chr15:102797525-102798025	22	0	480	Hoxc10
chr3:67386910-67387410	22	157	471	Mfsd1
chr8:9977885-9978385	22	0	419	Abhd13
chr5:122043170-122043670	22	0	412	Aldh2
chr1:193398785-193399285	22	0	377	Dtl
chr8:70585570-70586070	22	152	364	Psd3
chr9:119487530-119488030	22	0	353	Scn5a
chr11:31269505-31270005	22	0	305	Stc2
chr6:121160840-121161340	22	26569	303	Tuba8
chr14:104866680-104867180	22	5366	285	Pou4f1
chr4:128561370-128561870	22	0	237	Trim62
chr9:40965090-40965590	22	0	236	2810457I06Rik
chr4:116269860-116270360	22	0	124	AK086654
chr13:89881750-89882250	22	0	116	Vcan

chr13:36209130-36209630	22	0	101	Fars2
chr11:74738880-74739380	22	0	74	Srr
chr1:192886720-192887220	22	0	61	Nsl1
chr7:25292000-25292500	22	0	46	Zfp428
chr10:105278330-105278830	22	0	46	Ccdc59
chr2:30247730-30248230	22	0	45	Dolpp1
chr14:32149675-32150175	22	0	16	Capn7
chr19:6057960-6058460	22	21	-12	Fau
chr1:173489060-173489560	22	500000	-13	Cd244
chr12:84828420-84828920	22	0	-13	Dpf3
chr4:149461340-149461840	22	0	-21	Gpr157
chr15:89185700-89186200	22	0	-36	Tmem112b
chr6:124705865-124706365	22	9853	-77	Atn1
chr2:157030125-157030625	22	0	-121	Rbl1
chr17:88197235-88197735	22	0	-152	Kcnk12
chr4:116828650-116829150	22	62	-164	LOC100042180
chr3:122432710-122433210	22	0	-179	Pde5a
chr4:124377460-124377960	22	13	-243	Fhl3
chr17:46168820-46169320	22	0	-372	Vegf
chr11:98210620-98211120	22	357	-439	Ppp1r1b
chr2:25146030-25146530	22	0	-459	AK076389
chr2:180511815-180512315	22	299	-461	Bhlhb4
chr14:68551860-68552360	22	77	-482	Dock5
chr9:110020180-110020680	22	106	-513	HELG
chr13:55313750-55314250	22	0	-564	Nsd1
chr1:133964880-133965380	22	306	-621	Mfsd4
chr2:4638190-4638690	22	68	-750	E130319B15Rik
chr1:38685900-38686400	22	1222	-2062	Aff3
chr2:154611505-154612005	22	4661	-5137	Raly
chr6:22230290-22230790	22	500000	-7689	Wnt16
chr13:63677125-63677625	22	2335	-10548	Ptch1
chr3:152364425-152364925	22	12345	-12388	Pigk
chr6:48071120-48071620	22	34525	-34779	Znf746
chr5:125444220-125444720	22	47211	-39374	3110032G18Rik
chr17:30064495-30064995	22	37861	-39919	Mdga1
chr8:28087730-28088230	22	0	-46350	Erlin2
chr3:149376270-149376770	21	500000	500000	
chr4:31281090-31281590	21	500000	500000	
chr4:150816910-150817410	21	42783	500000	
chr7:70901970-70902470	21	147	500000	
chr8:14306695-14307195	21	500000	500000	
chr9:56108120-56108620	21	500000	500000	
chr11:34444590-34445090	21	500000	500000	
chr11:91221660-91222160	21	500000	500000	
chr12:33280090-33280590	21	500000	500000	
chr15:87798285-87798785	21	500000	500000	

chr17:70697120-70697620	21	500000	500000	
chr18:43135390-43135890	21	500000	500000	
chr13:43929645-43930145	21	29944	49324	CD83
chr6:136951150-136951650	21	500000	48357	Pde6h
chr16:92780395-92780895	21	500000	45665	Runx1
chr4:145152390-145152890	21	14353	43805	BC086318
chr13:96711035-96711535	21	19907	43519	mKIAA1054
chr12:17736360-17736860	21	38268	38991	Hpcal1
chr17:35755720-35756220	21	48350	24669	Dpcr1
chr9:56865310-56865810	21	21914	22786	Ptpn9
chr17:30703090-30703590	21	9226	9773	Btbd9
chrX:55286825-55287325	21	1990	2900	Zic3
chr11:5962870-5963370	21	1879	2630	Camk2b
chr5:28791370-28791870	21	0	2007	Shh
chr1:173463390-173463890	21	29309	1783	Itln1
chr4:137950450-137950950	21	102	1742	LOC625638
chr11:59919525-59920025	21	0	1261	Rai1
chr4:147503420-147503920	21	1001	1136	2610109H07Rik
chr10:40604015-40604515	21	0	887	Wasf1
chr13:96901410-96901910	21	303	871	Sv2c
chr1:121317765-121318265	21	0	809	Inhbb
chr9:107611320-107611820	21	3	779	Sema3f
chr19:5456475-5456975	21	341	778	Ccdc85b
chr14:104866205-104866705	21	5841	760	Pou4f1
chr12:60319430-60319930	21	342	758	Fbxo33
chr5:15441095-15441595	21	191	740	Cacna2d1
chr6:146991765-146992265	21	251	724	Mrps35
chr3:137528005-137528505	21	0	693	H2afz
chr19:43750100-43750600	21	0	683	AK076978
chr16:91226215-91226715	21	0	671	Olig2
chr15:102087010-102087510	21	313	653	Rarg
chr11:20640620-20641120	21	0	616	Aftph
chr19:19184820-19185320	21	0	615	Rorb
chr9:122026810-122027310	21	49	604	Snrk
chr14:60244480-60244980	21	0	586	Tmem46
chr2:53051420-53051920	21	121	526	Arl6ip6
chr13:55200910-55201410	21	19	495	Uimc1
chr11:30785600-30786100	21	243	493	Gpr75
chr14:55050695-55051195	21	0	490	Mmp14
chrX:39264340-39264840	21	0	487	Thoc2
chr11:11925610-11926110	21	0	466	Grb10
chr11:98656970-98657470	21	0	391	4121402D02Rik
chr10:62485945-62486445	21	0	390	AK156477
chr11:88578915-88579415	21	0	377	Msi2
chr2:70401040-70401540	21	0	335	Gad1
chr8:126038695-126039195	21	0	306	Dbndd1

chr8:69384920-69385420	21	90	301	BC053440
chr3:53960340-53960840	21	374	296	Trpc4
chr19:4712440-4712940	21	53	283	Spnb3
chr15:90509325-90509825	21	0	243	Cpne8
chr2:65077040-65077540	21	1	169	Cobll1
chr18:7868740-7869240	21	0	143	Wac
chr3:35652950-35653450	21	0	141	Atp11b
chr13:99124050-99124550	21	0	101	Foxd1
chr15:98701280-98701780	21	174	85	BC012717
chr18:44821695-44822195	21	39	-26	Mcc
chr11:116514560-116515060	21	156	-37	Prcd
chr13:34129190-34129690	21	0	-69	Bphl
chr12:102956570-102957070	21	3	-109	Tc2n
chr10:107599095-107599595	21	0	-110	Ppp1r12a
chr4:124663260-124663760	21	0	-163	Rspo1
chr11:116689290-116689790	21	70	-181	Mxra7
chr6:99676445-99676945	21	0	-310	Prok2
chr15:97076970-97077470	21	160	-317	BC038822
chr2:119152025-119152525	21	0	-343	AK039958
chr9:44306510-44307010	21	0	-458	Bcl9l
chr11:69809660-69810160	21	0	-582	Phf23
chr11:72303790-72304290	21	329	-619	BC011467
chr17:24624855-24625355	21	178	-622	Caskin1
chr1:93697135-93697635	21	78	-668	Twist2
chr10:83903670-83904170	21	0	-705	Nuak1
chr1:184272215-184272715	21	750	-729	Fbxo28
chr14:58690540-58691040	21	0	-732	Fgf9
chr17:27650430-27650930	21	0	-904	Grm4
chr10:107767950-107768450	21	999	-1044	Pawr
chr4:130124090-130124590	21	1156	-1434	2610200G18Rik
chr1:90196945-90197445	21	22917	-2662	Trpm8
chr10:61444440-61444940	21	2641	-2835	Col13a1
chr1:90130370-90130870	21	28246	-4062	AK133186
chr1:90194660-90195160	21	20632	-4947	Trpm8
chr10:82815335-82815835	21	7168	-7412	D10Wsu102e
chr1:173481460-173481960	21	47379	-7613	Cd244
chr17:72012000-72012500	21	45912	-10350	4632412N22Rik
chr1:173475875-173476375	21	41794	-10702	Itln1
chr1:193932170-193932670	21	15788	-15976	Traf5
chr4:145037170-145037670	21	16024	-16692	NR_002888
chr4:131722460-131722960	21	22068	-18219	DQ713846
chrX:166446065-166446565	21	10049	-21445	AK019053
chr6:38613465-38613965	21	334	-26477	Klrg2
chr2:27698540-27699040	21	42893	-43414	Col5a1
chr4:146883530-146884030	21	500000	-43635	OTTMUSG00000010673
chr11:76436425-76436925	21	0	-45447	Abr

chr1:46657115-46657615	20	500000	500000	
chr1:48938160-48938660	20	500000	500000	
chr1:51428545-51429045	20	500000	500000	
chr1:181250480-181250980	20	500000	500000	
chr3:21597000-21597500	20	500000	500000	
chr3:143954395-143954895	20	500000	500000	
chr4:29829295-29829795	20	500000	500000	
chr4:146873310-146873810	20	500000	500000	
chr6:119058840-119059340	20	24	500000	
chr8:82025810-82026310	20	500000	500000	
chr10:89335735-89336235	20	0	500000	
chr10:95540035-95540535	20	500000	500000	
chr11:7265350-7265850	20	500000	500000	
chr11:71734480-71734980	20	500000	500000	
chr11:74335615-74336115	20	0	500000	
chr12:33279155-33279655	20	500000	500000	
chr16:3271560-3272060	20	500000	500000	
chr17:3004990-3005490	20	500000	500000	
chr17:67148530-67149030	20	500000	500000	
chr19:42745210-42745710	20	500000	500000	
chr4:145144950-145145450	20	21793	36365	BC086318
chr5:148035840-148036340	20	34245	35819	Gsx1
chr5:148177590-148178090	20	33642	34224	Flt3
chr7:25965000-25965500	20	247	27775	AK142847
chr6:100681930-100682430	20	27005	27453	Glt8d4
chr8:95698240-95698740	20	500000	23212	Ces3
chr5:122979340-122979840	20	26833	15562	AK006664
chr8:126057210-126057710	20	12141	14726	Gas8
chr8:87311550-87312050	20	2860	12438	Nfix
chr5:33072515-33073015	20	500000	12116	Pisd
chrX:33747190-33747690	20	500000	11458	Zcchc12
chr11:85657180-85657680	20	164	11314	Tbx2
chr1:173455100-173455600	20	21019	10073	Itln1
chr13:45625915-45626415	20	23028	9945	Gmpr
chr1:173498655-173499155	20	500000	9582	Cd244
chr15:82045110-82045610	20	1971	9405	Tnfrsf13c
chr7:4424230-4424730	20	28196	8030	Eps8l1
chr10:57687900-57688400	20	29942	7272	Dux
chr17:72946690-72947190	20	5840	6706	Alk
chr7:149851850-149852350	20	1808	6503	NR_002855
chr9:108733850-108734350	20	80	5450	Celsr3
chr4:147178680-147179180	20	500000	5018	AK163132
chr1:183675730-183676230	20	500000	4814	A230079K17Rik
chr11:102980920-102981420	20	1870	3532	Hexim1
chr3:89763790-89764290	20	211	3389	Atp8b2
chrX:130884300-130884800	20	41795	3189	Cenpi

chr9:58046210-58046710	20	0	3061	Islr2
chr11:98187945-98188445	20	163	2763	Neurod2
chr11:105802590-105803090	20	2189	2620	Cyb561
chr5:102091420-102091920	20	658	2059	Nkx6-1
chr2:31527750-31528250	20	1438	1744	Exosc2
chr5:35733165-35733665	20	1299	1650	Hmx1
chr5:28493590-28494090	20	62	1605	En2
chr8:87427025-87427525	20	0	1449	Klf1
chr10:120414750-120415250	20	0	1385	Lemd3
chr10:86954800-86955300	20	140	1354	Ascl1
chr7:4728055-4728555	20	187	1321	Il11
chr3:157230770-157231270	20	217	1165	Ptger3
chr11:52579085-52579585	20	561	1128	Fstl4
chr15:6659225-6659725	20	0	1093	4921505C17Rik
chr11:72223885-72224385	20	498	1079	Smtnl2
chr2:132705310-132705810	20	67	1037	B430119L13Rik
chr4:120339945-120340445	20	133	1028	Cited4
chr6:118146510-118147010	20	0	1001	Ret
chr3:52073045-52073545	20	0	1000	Foxo1
chr4:106295785-106296285	20	331	982	Ttc22
chr14:46276670-46277170	20	0	897	Ddhd1
chr5:34630045-34630545	20	0	677	Zfyve28
chr10:80756145-80756645	20	137	671	Pip5k1c
chr8:109619550-109620050	20	51	644	Terf2
chr13:59870260-59870760	20	164	639	Isca1
chr11:103511190-103511690	20	0	618	Rprml
chr13:58374685-58375185	20	0	613	Gkap1
chr14:76245495-76245995	20	0	608	Tpt1
chr9:123439250-123439750	20	186	501	Sacm1l
chr9:96097005-96097505	20	87	489	Tfdp2
chr4:147505255-147505755	20	102	477	Mad2l2
chr19:6047360-6047860	20	128	466	Syvn1
chr10:126337700-126338200	20	57	460	Xrcc6bp1
chr1:134287160-134287660	20	557	447	Tmcc2
chr7:87376820-87377320	20	227	431	Cib1
chr12:32035650-32036150	20	0	362	Dld
chr3:10330770-10331270	20	0	351	Impa1
chr11:86298270-86298770	20	0	349	0610013E23Rik
chr1:60099345-60099845	20	0	335	Ica1l
chr2:167486465-167486965	20	81	328	Tmem189
chr6:146582555-146583055	20	0	308	Tm7sf3
chr7:108469820-108470320	20	0	307	Stard10
chr4:108292575-108293075	20	0	265	Cc2d1b
chr18:24867930-24868430	20	0	235	Fhod3
chr17:33102760-33103260	20	0	224	Zfp472
chr15:85411550-85412050	20	0	211	Wnt7b

chr5:24092515-24093015	20	0	204	2010209O12Rik
chr10:58684150-58684650	20	0	194	Sept10
chr13:43399140-43399640	20	0	150	Gfod1
chr12:78339000-78339500	20	0	139	Fut8
chr7:74904275-74904775	20	0	102	Dmn
chr3:84063380-84063880	20	0	69	Trim2
chr19:28754250-28754750	20	0	66	Glis3
chr10:82800300-82800800	20	0	11	Slc41a2
chr4:107474395-107474895	20	449	-218	Lrp8
chr11:101286630-101287130	20	0	-234	1700113I22Rik
chr4:115500130-115500630	20	161	-316	Mobkl2c
chr15:54903090-54903590	20	131	-339	Taf2
chr15:75812540-75813040	20	136	-378	2410075B13Rik
chr7:123587105-123587605	20	0	-384	Pik3c2a
chr11:119409270-119409770	20	0	-387	Nptx1
chr17:3084190-3084690	20	0	-399	AK132606
chr11:71855340-71855840	20	110	-413	6720460F02Rik
chr19:24248650-24249150	20	0	-423	Tjp2
chr5:65827295-65827795	20	239	-465	Ugdh
chr2:19366550-19367050	20	163	-489	Ptf1a
chr12:113368355-113368855	20	0	-532	CS444510
chr16:60605765-60606265	20	0	-659	Epha6
chrX:166425290-166425790	20	0	-670	AK019053
chr2:153270840-153271340	20	153	-877	8430427H17Rik
chr9:31721650-31722150	20	0	-1031	Barx2
chr15:102794120-102794620	20	2562	-2925	Hoxc10
chr2:38359905-38360405	20	7063	-7064	Nek6
chr9:67679360-67679860	20	59	-8592	Vps13c
chr15:68850650-68851150	20	500000	-9244	etoile
chr15:89387400-89387900	20	553	-11106	Acr
chr11:114939960-114940460	20	12554	-12534	Rab37
chr2:31787955-31788455	20	17424	-17617	2810003C17Rik
chr5:15052115-15052615	20	500000	-19359	Speer4c
chr10:10841190-10841690	20	27563	-27787	Shprh
chr4:146896345-146896845	20	500000	-30820	OTTMUSG00000010673
chr8:91013695-91014195	20	31314	-31323	Nkd1
chr19:61340080-61340580	20	35716	-36069	Csf2ra
chr17:23655850-23656350	20	27521	-39537	EG619788
chr3:21575350-21575850	19	500000	500000	
chr3:82930710-82931210	19	203	500000	
chr4:144998515-144999015	19	500000	500000	
chr5:33775285-33775785	19	0	500000	
chr5:37898635-37899135	19	500000	500000	
chr5:118825390-118825890	19	5171	500000	
chr7:70903470-70903970	19	175	500000	
chr8:11181640-11182140	19	5904	500000	

chr9:11743120-11743620	19	500000	500000	
chr9:31840335-31840835	19	0	500000	
chr9:62281130-62281630	19	500000	500000	
chr9:70653760-70654260	19	500000	500000	
chr9:100221950-100222450	19	500000	500000	
chr10:6246450-6246950	19	500000	500000	
chr10:29725885-29726385	19	500000	500000	
chr10:49955850-49956350	19	500000	500000	
chr11:63113195-63113695	19	500000	500000	
chr11:71732835-71733335	19	500000	500000	
chr13:45932685-45933185	19	500000	500000	
chr13:95472870-95473370	19	148	500000	
chr14:18602190-18602690	19	500000	500000	
chr17:63086680-63087180	19	500000	500000	
chr19:54343055-54343555	19	500000	500000	
chr3:84233610-84234110	19	500000	49552	6330505N24Rik
chr13:24647430-24647930	19	500000	48427	AK139092
chr4:44678540-44679040	19	1245	44521	Pax5
chr5:52625465-52625965	19	43731	43796	AK079034
chr17:88154310-88154810	19	8429	42773	Kcnk12
chr17:67942540-67943040	19	500000	39272	Lrrc30
chr13:91096915-91097415	19	33926	34385	Rps23
chrX:11711355-11711855	19	667	25875	Bcor
chr12:36751150-36751650	19	17217	17664	Tspan13
chr5:33068830-33069330	19	500000	15801	Pisd
chrX:98509555-98510055	19	40364	15257	Nlgn3
chr2:36558430-36558930	19	500000	15157	Olfr346
chr4:139373535-139374035	19	3279	15097	Pax7
chr6:48468590-48469090	19	35873	14505	AK129160
chr1:90148840-90149340	19	24698	14408	AK133186
chr13:96234810-96235310	19	500000	13549	S100z
chr17:56640550-56641050	19	9921	11105	Znrf4
chr15:31376665-31377165	19	6202	6528	Ropn1l
chr7:127323350-127323850	19	500000	6211	Anks4b
chr7:148348160-148348660	19	6014	6177	Rnh1
chr9:62990400-62990900	19	66	4136	Lbxcor1
chr12:70754865-70755365	19	27197	3931	Sos2
chr16:17834490-17834990	19	318	3538	Car15
chr3:120962045-120962545	19	3130	3511	Tmem56
chr4:151499795-151500295	19	0	2988	Espn
chr17:13743730-13744230	19	500000	2977	BC068229
chr18:7001750-7002250	19	294	2753	Mkx
chr15:84682770-84683270	19	873	2508	Phf21b
chr14:104864475-104864975	19	7571	2490	Pou4f1
chrX:166422400-166422900	19	206	2220	AK019053
chr5:125340300-125340800	19	1453	2040	Ccdc92

chr7:85720930-85721430	19	1629	1680	Ntrk3
chr17:3077690-3078190	19	6385	1611	AK206103
chr2:144994980-144995480	19	1167	1382	Slc24a3
chr15:36722560-36723060	19	0	1188	Ywhaz
chr6:91359940-91360440	19	236	1172	Wnt7a
chr19:3768920-3769420	19	68	1149	Suv420h1
chr15:75898900-75899400	19	383	1009	Scrib
chr16:8737210-8737710	19	341	974	Usp7
chr4:139387725-139388225	19	0	907	Pax7
chr17:80294350-80294850	19	0	862	Arl6ip2
chr14:57431415-57431915	19	23	840	2410022M11Rik
chr4:47221450-47221950	19	10	817	Col15a1
chr19:53675485-53675985	19	195	799	Smc3
chr9:96263710-96264210	19	73	757	Atp1b3
chr1:77510670-77511170	19	4	742	Epha4
chr10:57694445-57694945	19	23397	727	Dux
chr1:90200325-90200825	19	26297	718	Trpm8
chr1:193641740-193642240	19	3138	716	AK017306
chr3:109144020-109144520	19	199	670	Vav3
chr8:106864910-106865410	19	38	667	Cmtm3
chr12:32634190-32634690	19	0	647	Hbp1
chr8:85388100-85388600	19	0	638	AK170194
chr2:38207200-38207700	19	0	623	Lhx2
chr10:126616785-126617285	19	260	602	C78409
chr2:163679830-163680330	19	243	595	Kcnk15
chr4:73898170-73898670	19	79	569	Jmjd2c
chrX:166416040-166416540	19	6566	558	AK149987
chr15:82978980-82979480	19	50	535	Poldip3
chr5:28785085-28785585	19	0	522	AK053224
chr3:62142935-62143435	19	0	487	4631416L12Rik
chr3:105253925-105254425	19	755	469	AK018929
chr6:100620420-100620920	19	0	455	Shq1
chr11:103814665-103815165	19	12873	454	Nsf
chr5:141124280-141124780	19	91	447	Ttyh3
chr8:123606235-123606735	19	99	446	AK087264
chr17:46765700-46766200	19	0	434	Ptk7
chr1:74818640-74819140	19	108	425	Wnt6
chr2:30329075-30329575	19	0	393	Ier5l
chr6:63206975-63207475	19	0	375	Grid2
chr14:68702055-68702555	19	0	365	Nefl
chr18:73974430-73974930	19	95	365	Me2
chr9:96378400-96378900	19	0	363	Rnf7
chr6:18464220-18464720	19	46	354	Cttnbp2
chr6:72848685-72849185	19	0	350	Kcmf1
chr11:11014835-11015335	19	500	339	Vwc2
chr2:73748795-73749295	19	0	305	Atp5g3

chr11:70246170-70246670	19	28	266	Arrb2
chr16:4212910-4213410	19	0	243	Crebbp
chr1:133724540-133725040	19	0	237	Slc41a1
chr17:8627270-8627770	19	0	233	T
chr13:63341315-63341815	19	0	215	apo
chr9:64880540-64881040	19	0	206	Dpp8
chr16:36828030-36828530	19	0	64	Eaf2
chr5:136611565-136612065	19	0	55	1200011O22Rik
chr3:40754500-40755000	19	0	37	1700108L22Rik
chr11:98543640-98544140	19	0	23	Psmd3
chr4:154010720-154011220	19	0	11	Prdm16
chr5:122841690-122842190	19	0	4	Arpc3
chr10:80532640-80533140	19	0	-4	Thop1
chr7:52391580-52392080	19	7071	-29	Flt3l
chr4:47486985-47487485	19	0	-39	Alg2
chr8:36439270-36439770	19	0	-212	Ppp1r3b
chr4:119095010-119095510	19	0	-217	Zmynd12
chr15:100700620-100701120	19	479	-243	Scn8a
chr10:79818680-79819180	19	205	-273	6330514A18Rik
chr4:6117725-6118225	19	96	-276	3110003A22Rik
chr3:148652470-148652970	19	0	-277	Lphn2
chr3:97816905-97817405	19	76	-305	Notch2
chr2:154395730-154396230	19	47	-393	E2f1
chr10:84849920-84850420	19	0	-393	Btbd11
chr10:90633360-90633860	19	0	-404	Tmpo
chr10:80306950-80307450	19	153	-426	BC072620
chr15:99420745-99421245	19	0	-463	Aqp5
chr17:24591080-24591580	19	270	-479	BC026600
chr15:91022510-91023010	19	7103	-523	Abcd2
chr7:73006320-73006820	19	102	-528	Pcsk6
chr8:97604280-97604780	19	247	-570	Katnb1
chr14:73110130-73110630	19	0	-571	Fndc3a
chr10:42303740-42304240	19	0	-597	Nr2e1
chr17:26252670-26253170	19	2154	-655	Tmem8
chr1:183142050-183142550	19	0	-800	Wdr26
chr17:5940310-5940810	19	333	-817	Synj2
chr9:32349640-32350140	19	917	-938	Fli1
chr18:81184210-81184710	19	0	-1144	Sall3
chr5:31396970-31397470	19	355	-1350	Slc30a3
chr11:120091490-120091990	19	496	-2520	Bahcc1
chr2:179802390-179802890	19	2586	-2657	Gtpbp5
chr15:58917010-58917510	19	3168	-3717	mKIAA0429
chr13:15091695-15092195	19	500000	-4033	AK085253
chr7:144510775-144511275	19	3008	-4898	Ebf3
chr3:113338255-113338755	19	5313	-5439	mKIAA1839
chr1:173471270-173471770	19	37189	-6097	Itln1

chr3:113339115-113339615	19	6173	-6299	mKIAA1839
chr4:147194960-147195460	19	47266	-11262	AK163132
chr2:101866100-101866600	19	32364	-13435	AK079670
chr17:74008090-74008590	19	500000	-18781	AK085353
chr10:83161970-83162470	19	23199	-23422	AK153590
chr8:99009790-99010290	19	500000	-26199	AK006709
chr8:22136615-22137115	19	500000	-28358	Defcr21
chr11:103965460-103965960	19	28128	-28485	Crhr1
chr16:92730815-92731315	19	31930	-33493	Runx1
chr10:57165120-57165620	19	40625	-40921	Hsf2
chr4:146885705-146886205	19	500000	-41460	OTTMUSG00000010673
chr1:12634600-12635100	19	500000	-47759	Sulf1
chr1:93751995-93752495	18	500000	500000	
chr1:128926360-128926860	18	500000	500000	
chr1:169032225-169032725	18	500000	500000	
chr1:197105190-197105690	18	37385	500000	
chr3:143230230-143230730	18	500000	500000	
chr4:71687895-71688395	18	500000	500000	
chr7:136967245-136967745	18	500000	500000	
chr7:150364440-150364940	18	500000	500000	
chr10:109892820-109893320	18	0	500000	
chr10:123113130-123113630	18	500000	500000	
chr11:91222245-91222745	18	500000	500000	
chr12:81445540-81446040	18	4723	500000	
chr13:16570310-16570810	18	500000	500000	
chr16:3231585-3232085	18	500000	500000	
chr18:81512375-81512875	18	500000	500000	
chrX:4658460-4658960	18	500000	500000	
chrX:166611620-166612120	18	500000	500000	
chr11:85787960-85788460	18	500000	49641	AK135737
chr9:113888630-113889130	18	8923	46988	Fbxl2
chr4:99812905-99813405	18	44125	44760	Ror1
chr1:135060050-135060550	18	53	32261	Ppp1r15b
chr11:60200070-60200570	18	29922	30186	Atpaf2
chr4:107326095-107326595	18	29804	29836	Dmrtb1
chr15:79312140-79312640	18	3364	23280	Kcnj4
chr1:90151615-90152115	18	21923	17183	AK133186
chr1:90155840-90156340	18	17698	16669	A730008H23Rik
chr7:73394975-73395475	18	500000	15231	Lrrk1
chr1:173445010-173445510	18	10929	11652	Refbp2
chr5:135815250-135815750	18	10221	10244	Fkbp6
chr15:98410245-98410745	18	11832	9061	4930415O20Rik
chr18:36274335-36274835	18	46100	8729	4930471G03Rik
chr1:90116975-90117475	18	14851	8692	Ugt1a1
chr1:173457010-173457510	18	22929	8163	Itln1
chr8:126050200-126050700	18	11183	7716	Gas8

chr15:83725000-83725500	18	500000	6519	mKIAA1672
chr8:27272880-27273380	18	3745	4446	Thap1
chr11:102304760-102305260	18	3431	3966	BC025575
chr19:61300095-61300595	18	651	3916	Csf2ra
chr10:61946780-61947280	18	2006	2315	Vps26a
chr2:174111520-174112020	18	48	1950	Gnas
chr5:139232690-139233190	18	889	1887	BC004044
chr19:3769630-3770130	18	778	1859	Suv420h1
chr1:157587265-157587765	18	732	1641	Lhx4
chr5:31480635-31481135	18	1062	1631	Gtf3c2
chr12:117317480-117317980	18	1277	1465	Vipr2
chr11:72301780-72302280	18	317	1391	BC011467
chr4:150481050-150481550	18	432	1262	mKIAA0833
chr14:104865720-104866220	18	6326	1245	Pou4f1
chr11:120098990-120099490	18	0	1228	Bahcc1
chr11:102806315-102806815	18	651	1209	C1ql1
chr9:58672235-58672735	18	512	1167	Hcn4
chr3:104443465-104443965	18	353	1125	Slc16a1
chr12:81536175-81536675	18	0	1100	Wdr22
chr7:28192860-28193360	18	138	1084	Spnb4
chr11:5343540-5344040	18	173	1059	Znrf3
chr11:119407910-119408410	18	46	973	Nptx1
chr15:39030595-39031095	18	132	968	Rims2
chr2:180068200-180068700	18	0	927	Gata5
chr9:121629200-121629700	18	280	923	Nktr
chr5:139677205-139677705	18	226	832	Unc84a
chr10:77085610-77086110	18	252	795	Ube2g2
chr11:119464840-119465340	18	63	782	4932417H02Rik
chr2:27533250-27533750	18	51	780	Rxra
chrX:166423840-166424340	18	978	780	AK019053
chr3:68809360-68809860	18	0	717	Smc4
chr15:58654020-58654520	18	0	711	Tmem65
chr7:4609600-4610100	18	188	701	Saps1
chr12:101758085-101758585	18	186	666	AK046740
chr13:13683090-13683590	18	69	665	Lyst
chr19:45308810-45309310	18	0	665	Lbx1
chr3:152330220-152330720	18	107	633	Ak5
chr19:38198420-38198920	18	305	627	Rbp4
chr4:154865845-154866345	18	0	623	Gnb1
chr5:123228560-123229060	18	80	597	Camkk2
chr12:31950520-31951020	18	0	590	Lamb1-1
chr10:125403665-125404165	18	0	584	Lrig3
chr11:54600310-54600810	18	0	577	Cdc42se2
chr3:126300250-126300750	18	0	548	Camk2d
chr4:58924005-58924505	18	55	546	AI314180
chr2:77117865-77118365	18	61	533	Sestd1

chr12:35590890-35591390	18	227	508	Hdrpa
chr15:66117030-66117530	18	63	505	Kcnq3
chr1:135320060-135320560	18	0	478	Sox13
chr17:14416730-14417230	18	158	439	Smoc2
chr11:20012235-20012735	18	0	430	Actr2
chr6:43615530-43616030	18	500000	393	Tpk1
chr11:115280600-115281100	18	183	382	Atp5h
chr9:122481750-122482250	18	159	381	AK079255
chr6:117118710-117119210	18	0	380	Cxcl12
chr2:153117270-153117770	18	97	356	Kif3b
chr7:36370040-36370540	18	0	310	Rgs9bp
chr3:84283810-84284310	18	0	290	6330505N24Rik
chr4:33117760-33118260	18	0	287	AK076922
chr16:35363480-35363980	18	0	273	Sec22a
chr7:53965510-53966010	18	0	270	Saal1
chr17:71659790-71660290	18	0	264	Emilin2
chr15:6658365-6658865	18	0	233	4921505C17Rik
chr2:119063180-119063680	18	0	218	Spint1
chr2:65201620-65202120	18	500000	212	9330158F14Rik
chr19:4438885-4439385	18	37499	133	AK048995
chr16:64851785-64852285	18	0	125	Cggbp1
chr13:58907815-58908315	18	0	109	Ntrk2
chr11:4964760-4965260	18	11	86	Gas2l1
chr18:66450755-66451255	18	0	78	AK138348
chr6:120772410-120772910	18	0	42	Atp6v1e1
chr14:75415570-75416070	18	0	-13	5031414D18Rik
chr1:162964190-162964690	18	95	-26	Zbtb37
chr1:89051445-89051945	18	36	-33	Ecel1
chr3:96397860-96398360	18	0	-45	Polr3gl
chr10:98570470-98570970	18	345	-51	Galnt4
chr14:55544580-55545080	18	0	-80	Efs
chr2:57090775-57091275	18	30	-107	AK044432
chr7:123174430-123174930	18	477	-120	Sox6
chrX:130223250-130223750	18	0	-120	Pcdh19
chr6:29222250-29222750	18	6	-171	2310016C08Rik
chr17:35372685-35373185	18	545	-176	Nfkbil1
chr6:146590650-146591150	18	0	-200	Med21
chr6:84538605-84539105	18	125	-224	Cyp26b1
chr5:64483640-64484140	18	198	-298	Pgm1
chr9:8134360-8134860	18	36	-317	AK129341
chr11:4848180-4848680	18	187	-364	Nefh
chr11:6962875-6963375	18	573	-366	Adcy1
chr16:11983555-11984055	18	51	-400	2700045P11Rik
chr18:38369570-38370070	18	46	-405	Pcdh1
chr5:123229580-123230080	18	106	-423	Camkk2
chr11:74403850-74404350	18	181	-441	Garnl4

chr4:32050395-32050895	18	0	-451	Map3k7
chr7:99889435-99889935	18	0	-538	Rab30
chr4:131700690-131701190	18	298	-540	Oprd1
chr11:4846700-4847200	18	49	-573	Ap1b1
chr3:34548095-34548595	18	0	-581	Sox2
chr15:97835440-97835940	18	366	-615	Col2a1
chr2:70311990-70312490	18	1811	-739	Sp5
chr10:86956910-86957410	18	462	-756	Ascl1
chr10:79178355-79178855	18	401	-773	Hcn2
chr15:7759980-7760480	18	0	-780	Gdnf
chr1:90133635-90134135	18	31511	-797	AK133186
chr10:45206775-45207275	18	352	-825	Lin28b
chr17:30025430-30025930	18	295	-854	Mdga1
chr11:115129280-115129780	18	947	-974	Grin2c
chr19:26680525-26681025	18	0	-984	Smarca2
chr15:78550000-78550500	18	943	-1050	Lrrc62
chr15:79920175-79920675	18	1116	-1338	Syngr1
chr2:116945555-116946055	18	0	-1380	Spred1
chrX:166407640-166408140	18	14966	-2926	Fxy
chr1:173486125-173486625	18	500000	-2948	Cd244
chr11:55424030-55424530	18	500000	-2989	Glra1
chr8:124423640-124424140	18	6800	-3426	4F2 lc
chr2:165217040-165217540	18	3457	-3532	Zfp334
chr2:74501350-74501850	18	3639	-4125	Evx2
chr15:101789180-101789680	18	18375	-4713	Krt78
chr15:102792150-102792650	18	4532	-4895	Hoxc10
chr1:173484040-173484540	18	500000	-5033	Cd244
chr18:25219180-25219680	18	500000	-5287	Fhod3
chr18:13105765-13106265	18	5550	-5787	Osbpl1a
chr4:153544320-153544820	18	294	-6583	AK205032
chr8:87305245-87305745	18	2625	-7228	Nfix
chr4:138300540-138301040	18	500000	-7709	Ubxd3
chr1:90124790-90125290	18	22666	-9642	AK133186
chr19:55237550-55238050	18	500000	-17574	Tectb
chr5:24520220-24520720	18	500000	-18900	Prkag2
chr11:98359405-98359905	18	40047	-19847	Ikzf3
chr7:142932035-142932535	18	23887	-24244	Ki-67
chr15:102576510-102577010	18	217	-24261	Calcoco1
chr11:103966750-103967250	18	26838	-27195	Crhr1
chr10:43958380-43958880	18	29352	-29533	Atg5
chr11:50384925-50385425	18	30026	-30411	Adamts2
chr4:145022150-145022650	18	31044	-31712	NR_002888
chr1:23263100-23263600	18	0	-35202	CS444445
chr4:145163870-145164370	18	2873	-42900	OTTMUSG00000010173
chr10:57160820-57161320	18	44925	-45221	Hsf2
chr1:91477605-91478105	18	0	-46472	Centg2

chr1:135410780-135411280	17	500000	500000	
chr2:19190690-19191190	17	500000	500000	
chr3:159411645-159412145	17	500000	500000	
chr4:31343125-31343625	17	500000	500000	
chr4:102128060-102128560	17	500000	500000	
chr4:146858160-146858660	17	40840	500000	
chr5:33772650-33773150	17	188	500000	
chr8:89479615-89480115	17	500000	500000	
chr8:94599870-94600370	17	500000	500000	
chr8:122948935-122949435	17	24613	500000	
chr9:115442395-115442895	17	500000	500000	
chr10:43146490-43146990	17	29341	500000	
chr10:95634690-95635190	17	500000	500000	
chr10:101472390-101472890	17	500000	500000	
chr11:21729930-21730430	17	500000	500000	
chr11:118428770-118429270	17	1373	500000	
chr12:54073305-54073805	17	500000	500000	
chr14:105144480-105144980	17	500000	500000	
chr15:36642595-36643095	17	500000	500000	
chr17:30840260-30840760	17	500000	500000	
chr17:88146530-88147030	17	649	500000	
chrX:5193870-5194370	17	500000	500000	
chr5:77785710-77786210	17	46080	46435	Polr2b
chr14:48138300-48138800	17	45698	46315	Fbxo34
chr17:23570360-23570860	17	500000	45953	EG619788
chr4:145150930-145151430	17	15813	42345	BC086318
chr10:121117435-121117935	17	38545	38929	D930020B18Rik
chr9:48340325-48340825	17	36546	37029	EG434402
chr3:122011045-122011545	17	14198	33964	Dnttip2
chr8:9942180-9942680	17	33690	33892	Lig4
chr5:101070485-101070985	17	32088	32534	Coq2
chr19:12081220-12081720	17	40574	29759	Olfr1423
chr6:56282485-56282985	17	500000	29569	Pde1c
chr5:115047505-115048005	17	13110	29496	BC057022
chr1:34808010-34808510	17	331	29492	mKIAA1112
chr4:108591630-108592130	17	39889	28986	Rab3b
chr16:85427970-85428470	17	500000	28329	AK080982
chr19:53302185-53302685	17	500000	27252	AK005607
chr9:58860410-58860910	17	22855	23587	Neo1
chr4:147845970-147846470	17	23244	23505	Frap1
chr9:122504785-122505285	17	23194	23416	AK079255
chr18:32173530-32174030	17	500000	22832	Myo7b
chr6:112874715-112875215	17	500000	22294	Srgap3
chr3:121844195-121844695	17	500000	21498	Abca4
chr11:75215865-75216365	17	42685	20009	Serpinf1
chr2:177892710-177893210	17	15609	16239	Phactr3

chr8:108246530-108247030	17	14736	14590	4933405L10Rik
chr11:74191335-74191835	17	500000	13413	Olfr412
chr10:126528240-126528740	17	0	12528	Centg1
chr8:108244290-108244790	17	12496	12350	4933405L10Rik
chr1:90120090-90120590	17	17966	11807	Ugt1a1
chr13:113825350-113825850	17	0	10995	2310016C16Rik
chr1:90162420-90162920	17	11118	10089	A730008H23Rik
chr4:151722335-151722835	17	9362	9826	Chd5
chr14:69780210-69780710	17	12237	9439	AK007038
chr13:95654175-95654675	17	0	8844	Otp
chr17:56642990-56643490	17	7481	8665	Znrf4
chr13:55573250-55573750	17	0	7832	Prr7
chr4:146981540-146982040	17	500000	7747	2610305D13Rik
chr1:173495650-173496150	17	500000	6577	Cd244
chr4:62174320-62174820	17	6058	6357	Alad
chr4:146983850-146984350	17	500000	5437	2610305D13Rik
chr1:157583910-157584410	17	0	4996	Lhx4
chr10:92293920-92294420	17	500000	4920	BC065048
chr8:74030680-74031180	17	552	4699	Plvap
chr12:54354060-54354560	17	0	4647	Npas3
chr2:130727280-130727780	17	4473	4584	AK042707
chr15:76347750-76348250	17	11	4558	Scrt1
chr5:140388040-140388540	17	39	4394	A930017N06Rik
chr4:146985050-146985550	17	500000	4237	2610305D13Rik
chr9:40685955-40686455	17	0	3996	Bsx
chr8:74031690-74032190	17	0	3689	Plvap
chr11:89159430-89159930	17	0	3653	Nog
chr5:148003365-148003865	17	1770	3344	Gsx1
chr10:11066315-11066815	17	2955	3323	Epm2a
chr17:13743395-13743895	17	500000	3312	BC068229
chr6:38583730-38584230	17	2368	3258	Klrg2
chr4:62177775-62178275	17	2603	2902	Alad
chr7:16897480-16897980	17	0	2744	Bbc3
chr1:90111025-90111525	17	8901	2742	Ugt1a1
chr10:28866185-28866685	17	42	2634	6330407J23Rik
chr10:78993745-78994245	17	2122	2491	Ppap2c
chr11:74180400-74180900	17	500000	2478	Olfr412
chr1:121316200-121316700	17	421	2374	Inhbb
chrX:99444645-99445145	17	491	2212	Cited1
chr3:95121130-95121630	17	1766	2207	Lass2
chr12:84826595-84827095	17	0	1812	Dpf3
chr15:101782660-101783160	17	24895	1807	Krt78
chr7:26473570-26474070	17	512	1800	Tgfb1
chr5:103959920-103960420	17	500000	1586	Ptpn13
chr4:141092785-141093285	17	0	1476	Spen
chr4:151501350-151501850	17	846	1433	Espn

chr13:49241735-49242235	17	0	1397	Wnk2
chr11:105947220-105947720	17	905	1255	Map3k3
chr6:126594335-126594835	17	0	1233	Kcna1
chr2:126958215-126958715	17	901	1173	Ncaph
chr18:10726530-10727030	17	147	1158	Mib1
chr7:35905210-35905710	17	0	1149	Cebpa
chr16:93684290-93684790	17	465	1077	Cbr3
chr3:116125685-116126185	17	184	1014	Cdc14a
chr7:19864190-19864690	17	73	957	C79127
chr6:29686195-29686695	17	0	943	Smo
chr19:59419095-59419595	17	0	924	Pdzd8
chr5:132013400-132013900	17	0	906	Auts2
chr8:118229620-118230120	17	0	901	Maf
chr3:61169050-61169550	17	0	872	Rap2b
chr3:105255835-105256335	17	97	838	Kcnd3
chr4:104781415-104781915	17	366	808	Prkaa2
chr14:13177325-13177825	17	0	766	Fezf2
chr19:43516210-43516710	17	302	760	Cnnm1
chr5:23958495-23958995	17	4950	751	Centg3
chr19:46391390-46391890	17	440	738	Psd
chr8:91045750-91046250	17	311	732	Nkd1
chr19:12670080-12670580	17	15	725	AK039403
chr5:72894490-72894990	17	155	706	Corin
chr8:4324160-4324660	17	27	689	Elavl1
chr5:54390245-54390745	17	91	687	Stim2
chr10:76880095-76880595	17	170	672	Adarb1
chr18:60907190-60907690	17	828	633	Ndst1
chr2:154483140-154483640	17	139	629	Chmp4b
chr12:113369500-113370000	17	430	613	CS444510
chr4:88367555-88368055	17	0	606	Klhl9
chr5:140020195-140020695	17	0	594	Uncx4
chr7:137690020-137690520	17	52	592	Nsmce4a
chr10:24431080-24431580	17	181	577	Enpp1
chr7:87406430-87406930	17	276	575	fi58gm
chr3:138737350-138737850	17	0	562	Rap1gds1
chr9:72833680-72834180	17	31	546	Ccpg1
chr10:45297980-45298480	17	0	536	Hace1
chr4:122513765-122514265	17	49052	531	Ppt1
chr11:117128045-117128545	17	246	530	Sept9
chr14:68742330-68742830	17	0	480	Nefm
chr3:33699345-33699845	17	85	479	Ttc14
chr6:42273860-42274360	17	186	468	6330503C03Rik
chr3:102274055-102274555	17	3	455	Ngfb
chr19:9969270-9969770	17	4242	440	Incenp
chr9:121552000-121552500	17	22	417	Vipr1
chr4:153331495-153331995	17	52	400	A430005L14Rik

chr15:87455795-87456295	17	0	386	AW049604
chr4:114581990-114582490	17	75	379	AK078937
chr5:121834960-121835460	17	86	369	Trafd1
chr4:131699790-131700290	17	10	360	Oprd1
chr5:116872275-116872775	17	22018	347	Hspb8
chr12:74146855-74147355	17	0	345	Six1
chr12:72075770-72076270	17	0	341	Psma3
chr15:82105785-82106285	17	390	334	Sept3
chr9:114472910-114473410	17	0	326	Trim71
chr1:136456845-136457345	17	0	324	Jarid1b
chr13:46822385-46822885	17	0	320	Nup153
chr8:97219585-97220085	17	0	306	Pllp
chr6:84538110-84538610	17	274	271	Cyp26b1
chr5:107718020-107718520	17	0	268	Tgfbr3
chr5:72950320-72950820	17	0	267	Nfxl1
chr11:116393790-116394290	17	0	267	Sphk1
chr7:25084055-25084555	17	0	262	Zfp61
chr8:97361430-97361930	17	242	261	Ciapin1
chr1:183140990-183141490	17	0	260	Wdr26
chr15:78872350-78872850	17	495	243	Galr3
chr1:38054820-38055320	17	0	216	Eif5b
chr16:57549320-57549820	17	0	215	4631422O05Rik
chr3:58329230-58329730	17	0	195	D3Ucla1
chr4:82150560-82151060	17	0	194	Nfib
chr4:116299665-116300165	17	0	186	Nasp
chr12:106247590-106248090	17	0	178	4831426I19Rik
chr12:88812860-88813360	17	0	138	Snw1
chr11:98843945-98844445	17	0	134	Gjc1
chr17:24625610-24626110	17	0	133	Caskin1
chr9:77961095-77961595	17	0	130	Ick
chr16:72663260-72663760	17	0	117	Robo1
chr9:110782220-110782720	17	0	116	Tmie
chr11:101414030-101414530	17	990	109	Nbr1
chr3:55987285-55987785	17	0	87	nbea
chr17:34258360-34258860	17	0	81	Brd2
chr12:73185990-73186490	17	0	59	2810055F11Rik
chr2:19580270-19580770	17	0	48	AK188320
chr5:143996665-143997165	17	0	29	Zfp12
chr11:53583355-53583855	17	0	26	Irf1
chr11:3352220-3352720	17	0	-4	AK018450
chr11:74537940-74538440	17	0	-19	Pafah1b1
chr4:21612900-21613400	17	147	-41	Prdm13
chr5:149364170-149364670	17	0	-56	Ubl3
chr4:32886875-32887375	17	0	-114	Lyrm2
chr10:62706760-62707260	17	0	-115	AK018967
chr12:93024770-93025270	17	7368	-145	Ston2

chr6:83027915-83028415	17	0	-218	Pcgf1
chr19:57082685-57083185	17	0	-230	AK158196
chr15:82104880-82105380	17	243	-234	Sept3
chr12:82731855-82732355	17	139	-250	Ttc9
chr7:54175305-54175805	17	0	-256	Tsg101
chr3:88139640-88140140	17	62	-291	Smg5
chr2:31939275-31939775	17	0	-301	A130092J06Rik
chr2:26998045-26998545	17	180	-302	C630035N08Rik
chr4:45839770-45840270	17	299	-322	Igfbpl1
chr9:102407865-102408365	17	108	-352	Ky
chr4:130347820-130348320	17	196	-381	Sdc3
chr9:30749515-30750015	17	340	-382	Adamts8
chr15:80117015-80117515	17	289	-402	Cacna1i
chr12:72931410-72931910	17	422	-404	Daam1
chr4:134707745-134708245	17	0	-415	Runx3
chr3:129764015-129764515	17	260	-441	Sec24b
chr3:28980785-28981285	17	276	-463	6130401L20Rik
chr11:11926715-11927215	17	630	-639	Grb10
chr11:4647210-4647710	17	471	-680	Cabp7
chr15:97078165-97078665	17	147	-698	Amigo2
chr10:81037325-81037825	17	342	-701	Tle2
chr11:103634580-103635080	17	26	-707	Wnt3
chr6:99641660-99642160	17	140	-762	Gpr27
chr9:95459100-95459600	17	265	-885	Paqr9
chr6:90411475-90411975	17	545	-894	Klf15
chr4:136832345-136832845	17	102	-954	Wnt4
chr16:90385400-90385900	17	328	-991	Hunk
chr12:62624280-62624780	17	175	-1088	Lrfn5
chr19:44830520-44831020	17	0	-1113	Pax2
chr13:94073385-94073885	17	0	-1118	Homer1
chr6:85136490-85136990	17	751	-1184	Emx1
chr10:43297300-43297800	17	595	-1424	Cd24a
chr5:75550485-75550985	17	1317	-1455	Pdgfra
chr8:87122860-87123360	17	17865	-2177	Cacna1a
chr13:29121770-29122270	17	500000	-2242	AK150110
chr4:128379705-128380205	17	24620	-2286	Phc2
chr1:173505545-173506045	17	500000	-3398	2b4
chr4:41590665-41591165	17	3643	-3581	2310040A07Rik
chr4:62166870-62167370	17	3776	-3887	Hdhd3
chr3:96374430-96374930	17	12804	-3957	AK143784
chr15:74389680-74390180	17	4407	-4072	Bai1
chr3:93624655-93625155	17	500000	-5036	Tdpoz3
chr4:41592655-41593155	17	5633	-5571	2310040A07Rik
chr15:12499060-12499560	17	500000	-6139	AK195788
chr2:167475040-167475540	17	11506	-6590	AK177731
chr8:86636635-86637135	17	17732	-8225	BC057552

chr15:12495610-12496110	17	500000	-9589	AK195788
chr19:3312935-3313435	17	10153	-10162	Cpt1a
chr15:89388290-89388790	17	0	-10216	Acr
chr1:90184740-90185240	17	10712	-10859	A730008H23Rik
chr3:136992805-136993305	17	500000	-11051	Emcn
chr2:101867570-101868070	17	30894	-11965	AK079670
chr11:117460140-117460640	17	500000	-12268	AK019332
chr17:23835405-23835905	17	1094	-14671	Cldn9
chr7:20058950-20059450	17	105	-15358	Mark4
chr2:31509955-31510455	17	0	-16051	Exosc2
chr18:39429840-39430340	17	500000	-16090	mKIAA0621
chr11:6636050-6636550	17	500000	-16261	AK076664
chr8:128240575-128241075	17	17202	-17303	2310079N02Rik
chr11:115720340-115720840	17	0	-20960	Myo15b
chr3:28138595-28139095	17	22616	-23290	AK039113
chr4:146709890-146710390	17	500000	-25276	AK145533
chr17:47175830-47176330	17	28598	-28639	Ubr2
chr9:96560460-96560960	17	28717	-28756	gap1m
chr2:31496660-31497160	17	0	-29346	Exosc2
chr17:85978685-85979185	17	28540	-34012	Six3
chr1:183808705-183809205	17	36071	-36493	Lbr
chr9:77442615-77443115	17	500000	-41673	Klhl31
chr8:48957400-48957900	17	500000	-44473	BC040690
chr11:18964090-18964590	17	36726	-45658	Meis1
chr18:77496345-77496845	17	3051	-49893	Loxhd1
chr1:5906770-5907270	16	0	500000	
chr1:181251030-181251530	16	500000	500000	
chr2:28150760-28151260	16	500000	500000	
chr2:164937310-164937810	16	0	500000	
chr3:6115990-6116490	16	500000	500000	
chr3:68672805-68673305	16	24	500000	
chr3:78705905-78706405	16	500000	500000	
chr3:143286310-143286810	16	500000	500000	
chr4:31107835-31108335	16	500000	500000	
chr4:105928370-105928870	16	500000	500000	
chr6:118272650-118273150	16	500000	500000	
chr8:11967800-11968300	16	500000	500000	
chr8:86899545-86900045	16	38366	500000	
chr9:62280420-62280920	16	500000	500000	
chr10:22794860-22795360	16	500000	500000	
chr10:29725125-29725625	16	500000	500000	
chr10:95129440-95129940	16	500000	500000	
chr10:109892320-109892820	16	94	500000	
chr10:126185350-126185850	16	500000	500000	
chr11:35859205-35859705	16	1270	500000	
chr13:85266435-85266935	16	500000	500000	

chr14:10101630-10102130	16	500000	500000	
chr14:33741870-33742370	16	500000	500000	
chr14:38400050-38400550	16	500000	500000	
chr14:59472880-59473380	16	500000	500000	
chr15:36643570-36644070	16	500000	500000	
chr16:96787705-96788205	16	500000	500000	
chr17:48874290-48874790	16	500000	500000	
chr17:49333335-49333835	16	500000	500000	
chr17:86303660-86304160	16	500000	500000	
chr18:81513160-81513660	16	500000	500000	
chr10:74918440-74918940	16	500000	48912	Upb1
chr5:14872360-14872860	16	500000	42278	ENSMUSG00000033219
chr7:82412155-82412655	16	41324	40792	Sv2b
chr18:80492825-80493325	16	0	40027	Pqlc1
chr11:97396620-97397120	16	278	39569	P140
chr13:50018250-50018750	16	500000	31166	DQ690487
chr11:60197220-60197720	16	30849	30589	Lrrc48
chr12:81330650-81331150	16	29424	30402	Actn1
chr19:12080595-12081095	16	39949	29340	Osbp
chr4:146961000-146961500	16	500000	28287	2610305D13Rik
chrX:106317940-106318440	16	500000	27445	Sh3bgrl
chr8:90114995-90115495	16	500000	27440	4933402J07Rik
chr1:58670590-58671090	16	27569	27398	Ndufb3
chr10:90318050-90318550	16	25654	25713	Anks1b
chr17:35754895-35755395	16	49175	25494	Dpcr1
chr4:128710300-128710800	16	15329	24676	AK019743
chr12:113619535-113620035	16	500000	24237	AK054515
chr15:89353560-89354060	16	565	22097	Shank3
chr4:134508330-134508830	16	21196	21686	Syf2
chr16:13447420-13447920	16	478	21177	AK131759
chr17:35759420-35759920	16	44650	20969	Dpcr1
chr6:91236470-91236970	16	1005	20848	Fbln2
chr4:41566340-41566840	16	20368	20744	2310040A07Rik
chr4:45715050-45715550	16	500000	18840	Gm829
chr8:95921445-95921945	16	500000	18639	DQ704652
chr1:90152870-90153370	16	20668	18438	AK133186
chr14:35383555-35384055	16	20990	17987	Ldb3
chr17:24193850-24194350	16	15948	16303	Kctd5
chr15:100607645-100608145	16	8661	15718	Slc4a8
chr11:74193170-74193670	16	500000	15248	Olfr412
chr13:55917700-55918200	16	8889	14835	Pitx1
chr4:139374875-139375375	16	1939	13757	Pax7
chr1:183684370-183684870	16	500000	13454	A230079K17Rik
chr8:87634200-87634700	16	27039	12543	Zfp791
chr4:146978035-146978535	16	500000	11252	2610305D13Rik
chr17:30702490-30702990	16	9826	10373	Btbd9

chr11:74187150-74187650	16	500000	9228	Olfr412
chr13:45624460-45624960	16	21573	8490	Gmpr
chr2:93816390-93816890	16	27	8305	Alkbh3
chr8:91151560-91152060	16	0	8216	9130017C17Rik
chr5:120889630-120890130	16	0	7971	Lhx5
chr1:75483780-75484280	16	181	7876	A230078I05Rik
chr1:90115825-90116325	16	13701	7542	Ugt1a1
chr4:44715605-44716105	16	141	7456	Pax5
chr5:139793915-139794415	16	6781	7252	Centa1
chr15:98823300-98823800	16	46	7155	AK016307
chr5:150249170-150249670	16	500000	7140	4930588N13Rik
chr17:25382370-25382870	16	5082	5477	0610007P22Rik
chr11:102303260-102303760	16	3319	5466	BC025575
chr11:84724620-84725120	16	4793	4987	Znhit3
chr15:99060010-99060510	16	4966	4854	Kcnh3
chr19:44834960-44835460	16	1400	3327	Pax2
chr1:157585615-157586115	16	754	3291	Lhx4
chr15:60653070-60653570	16	1205	3194	BC020326
chr9:24952760-24953260	16	2579	3064	Herpud2
chr4:62160010-62160510	16	2806	2973	Hdhd3
chr8:97511070-97511570	16	500000	2818	Gpr56
chr5:24603630-24604130	16	1507	2367	Prkag2
chr9:114471015-114471515	16	133	2221	Trim71
chr4:62160820-62161320	16	1996	2163	Hdhd3
chr11:32188850-32189350	16	21368	2137	F830116E18Rik
chr11:120048910-120049410	16	126	2009	AK164256
chr8:94323240-94323740	16	94	1782	Irx3
chr9:102409920-102410420	16	1369	1703	Ky
chr9:69046680-69047180	16	500000	1616	Rora
chr1:39632385-39632885	16	735	1614	Rnf149
chr1:74903010-74903510	16	217	1589	Cdk5r2
chr3:121949560-121950060	16	622	1301	Gclm
chr8:17533895-17534395	16	572	1239	Csmd1
chr10:53098205-53098705	16	603	1201	AK039734
chr2:103811260-103811760	16	441	1131	Lmo2
chr8:95199160-95199660	16	767	1110	Irx6
chr9:51856905-51857405	16	36	1110	Rdx
chr10:127897145-127897645	16	480	1046	Smarcc2
chr9:114472210-114472710	16	0	1026	Trim71
chr7:148109050-148109550	16	488	992	Nlrp6
chr4:124335610-124336110	16	0	972	Pou3f1
chr2:131318390-131318890	16	350	962	Smox
chr16:76372100-76372600	16	177	943	Nrip1
chr9:56843465-56843965	16	69	941	Ptpn9
chr9:107436960-107437460	16	76	935	Tmem115
chr6:52209740-52210240	16	0	883	Hoxa13

chr15:8393370-8393870	16	2	842	Nipbl
chr15:75811320-75811820	16	308	842	2410075B13Rik
chr6:126689120-126689620	16	0	841	Kcna6
chr9:100544590-100545090	16	0	799	Stag1
chr12:3427415-3427915	16	0	782	Asxl2
chr7:133224900-133225400	16	0	774	AK082731
chr2:153269200-153269700	16	0	763	8430427H17Rik
chr8:72840230-72840730	16	501	752	Lass1
chr4:155570610-155571110	16	0	736	Agrn
chr4:132088870-132089370	16	90	729	Atpif1
chr2:130522280-130522780	16	108	724	Slc4a11
chr8:91567110-91567610	16	0	700	Sall1
chr1:54250930-54251430	16	6	697	Hecw2
chr11:120094655-120095155	16	0	645	Bahcc1
chr4:135576680-135577180	16	130	633	Tceb3
chr10:98571110-98571610	16	985	589	Galnt4
chr1:166387655-166388155	16	0	580	Atp1b1
chr11:75007805-75008305	16	0	561	Rtn4rl1
chr8:114377410-114377910	16	2	549	Cfdp1
chr11:103994480-103994980	16	0	535	Crhr1
chr6:72184790-72185290	16	0	530	Atoh8
chr10:4522860-4523360	16	56	513	Mtrf1l
chr4:149656270-149656770	16	693	496	Rere
chr12:81620220-81620720	16	0	494	Galntl1
chr4:115500935-115501435	16	126	489	Mobkl2c
chr2:172765070-172765570	16	0	473	Bmp7
chr14:55544030-55544530	16	410	470	Efs
chr14:41779380-41779880	16	0	465	Tspan14
chr2:128644250-128644750	16	230	462	Tmem87b
chr6:149049490-149049990	16	0	461	D030011O10Rik
chr2:146839000-146839500	16	0	455	Xrn2
chr11:112643740-112644240	16	0	453	Sox9
chr9:121904440-121904940	16	92	403	C85492
chr15:31460140-31460640	16	0	401	March6
chr11:71565015-71565515	16	91	388	BC030477
chr9:42913175-42913675	16	84	375	Arhgef12
chr1:164501070-164501570	16	0	362	Vamp4
chr5:29895885-29896385	16	0	354	Ube3c
chr7:82600850-82601350	16	0	347	Akap13
chr19:4926470-4926970	16	1514	338	Dpp3
chr8:73284250-73284750	16	57	319	BC051227
chr7:127986460-127986960	16	0	314	Eef2k
chr13:31650360-31650860	16	0	305	Foxq1
chr17:27340150-27340650	16	126	292	Lemd2
chr6:71093830-71094330	16	0	289	Thnsl2
chr5:34338660-34339160	16	0	278	Nat8l

chr1:165924390-165924890	16	0	271	BC055324
chr15:102784960-102785460	16	0	254	Hoxc11
chr10:126602910-126603410	16	0	246	B4galnt1
chr4:126413020-126413520	16	0	242	Tcfap2e
chr10:22451260-22451760	16	0	228	Tbpl1
chr1:180267415-180267915	16	0	177	Hnrnpu
chr6:52195345-52195845	16	0	170	Hoxa11
chr8:72655130-72655630	16	0	149	2310073E15Rik
chr5:5379800-5380300	16	0	146	Pftk1
chr5:115584600-115585100	16	0	133	Unc119b
chr9:66440405-66440905	16	0	131	Usp3
chr1:162125250-162125750	16	0	110	Mrps14
chr9:14849590-14850090	16	0	81	Panx1
chr13:120217235-120217735	16	0	79	Paip1
chr1:183668935-183669435	16	500000	67	A230079K17Rik
chr6:97160600-97161100	16	0	65	Arl6ip5
chr6:54942550-54943050	16	0	60	A030007L17Rik
chr6:127100675-127101175	16	108	53	AK187594
chr14:21167020-21167520	16	40508	35	Ecd
chr6:126689935-126690435	16	0	26	Kcna6
chr4:124743700-124744200	16	0	14	Snip1
chr1:84931400-84931900	16	21	7	Slc16a14
chr11:77893610-77894110	16	0	-14	Eral1
chr9:97011270-97011770	16	0	-61	Slc25a36
chr14:60216610-60217110	16	2	-80	Cdadc1
chr3:62142360-62142860	16	30	-88	4631416L12Rik
chr3:7613270-7613770	16	355	-94	Il7
chr4:58925460-58925960	16	0	-114	AI314180
chr2:119613120-119613620	16	0	-123	mKIAA1403
chr9:63869040-63869540	16	0	-163	Smad6
chr9:81756910-81757410	16	0	-190	4930486G11Rik
chr8:34752400-34752900	16	66	-203	Ubxd6
chr17:43938320-43938820	16	145	-229	Rcan2
chr5:111846695-111847195	16	0	-240	Mn1
chr1:50983860-50984360	16	0	-256	Tmeff2
chr8:123606950-123607450	16	0	-269	AK087264
chr9:51976725-51977225	16	152	-299	mKIAA1726
chr9:54798830-54799330	16	1	-320	AY074887
chr4:107704770-107705270	16	113	-326	Podn
chr12:99813415-99813915	16	76	-329	Kcnk10
chr9:62384950-62385450	16	246	-350	Coro2b
chrX:45807450-45807950	16	0	-355	Elf4
chr11:120391460-120391960	16	155	-380	Gcgr
chr11:89163465-89163965	16	0	-382	Nog
chr8:98012860-98013360	16	500000	-421	Csnk2a2
chr2:19368505-19369005	16	484	-455	AK053418

chr5:87175060-87175560	16	500000	-479	Tmprss11e
chr6:72850210-72850710	16	47	-488	Kcmf1
chr4:154417590-154418090	16	243	-578	Plch2
chr9:119977745-119978245	16	41425	-596	Cx3cr1
chr6:134741710-134742210	16	343	-609	Dusp16
chr11:107656160-107656660	16	395	-633	Cacng4
chr10:18563860-18564360	16	582	-766	Perp
chr11:49837725-49838225	16	435	-857	Rnf130
chr4:154229340-154229840	16	169	-866	B230396O12Rik
chr13:31718350-31718850	16	0	-940	AK142058
chr11:69615970-69616470	16	1085	-950	Tmem102
chr10:94408015-94408515	16	65	-1054	Plxnc1
chr8:73317150-73317650	16	771	-1065	mKIAA0561
chr15:96116620-96117120	16	0	-1082	1700124K17Rik
chr10:42895225-42895725	16	571	-1140	Sobp
chr12:53704230-53704730	16	500000	-1172	AK006587
chr13:53077340-53077840	16	381	-1183	Nfil3
chr13:55049200-55049700	16	601	-1342	Unc5a
chr14:55545870-55546370	16	312	-1370	Efs
chr11:120520490-120520990	16	428	-1383	Notum
chr16:60606515-60607015	16	421	-1409	Epha6
chr11:120096175-120096675	16	0	-1587	Bahcc1
chr2:180625770-180626270	16	185	-1603	AK045142
chr12:113382520-113383020	16	0	-1648	Kif26a
chr9:106789760-106790260	16	202	-1737	Rbm15b
chr6:124809290-124809790	16	1631	-1836	Leprel2
chr4:120623615-120624115	16	110	-1843	Zfp69
chr8:122384090-122384590	16	1754	-1864	BC025816
chr2:130119480-130119980	16	744	-1944	Ebf4
chr2:180626250-180626750	16	59	-2083	AK045142
chr3:87801545-87802045	16	426	-2171	Bcan
chr1:135553780-135554280	16	3434	-2447	Zc3h11a
chr1:90176370-90176870	16	2342	-2489	A730008H23Rik
chr15:100523250-100523750	16	35919	-2703	AK028111
chr12:118392780-118393280	16	311	-3289	AK138376
chr1:173484750-173485250	16	500000	-4323	Cd244
chr15:99050710-99051210	16	3192	-4446	Kcnh3
chr6:48409825-48410325	16	14431	-4572	sco-spondin
chr4:154421840-154422340	16	4493	-4828	Plch2
chr2:127662715-127663215	16	5239	-5433	Bub1
chr8:22158925-22159425	16	500000	-6048	Defcr21
chr1:90179935-90180435	16	5907	-6054	A730008H23Rik
chr8:97227110-97227610	16	7145	-7219	Pllp
chr1:137941575-137942075	16	5573	-7652	Cacna1s
chr15:101792140-101792640	16	15415	-7673	Krt78
chr15:98529760-98530260	16	22098	-8647	Ccdc65

chr1:90190825-90191325	16	16797	-8782	Trpm8
chr10:58929450-58929950	16	500000	-8793	Ccdc109a
chr19:38784340-38784840	16	500000	-13418	Plce1
chr15:82798440-82798940	16	4810	-14609	AK140580
chrX:166440830-166441330	16	4814	-16210	AK019053
chr11:81832540-81833040	16	500000	-16288	Ccl2
chr17:74006795-74007295	16	500000	-17486	AK085353
chr3:107517845-107518345	16	18455	-18630	Ahcyl1
chr1:91846775-91847275	16	8241	-19275	Gbx2
chr12:85084915-85085415	16	27527	-19445	Papln
chr4:146793630-146794130	16	12224	-20093	AK019745
chr4:146794435-146794935	16	13029	-20898	AK019745
chr1:91851500-91852000	16	12966	-24000	Gbx2
chr1:167669280-167669780	16	24306	-24380	Creg1
chr18:6087085-6087585	16	48179	-25366	Arhgap12
chr4:153442050-153442550	16	13078	-25515	Ccdc27
chr4:145026775-145027275	16	26419	-27087	NR_002888
chr4:145025760-145026260	16	27434	-28102	NR_002888
chr7:142936560-142937060	16	28412	-28769	Ki-67
chr15:77166840-77167340	16	29411	-29733	Rbm9
chr4:145023625-145024125	16	29569	-30237	NR_002888
chr10:117035010-117035510	16	31012	-31301	Cpm
chr11:114494495-114494995	16	35115	-35111	Rpl38
chr4:146890365-146890865	16	500000	-36800	OTTMUSG00000010673
chr16:5936330-5936830	16	500000	-39099	AK018984
chr10:117025570-117026070	16	40452	-40741	Cpm
chr6:97305475-97305975	16	500000	-43352	Frmd4b
chr5:89271785-89272285	16	43554	-44249	Slc4a4
chr4:146882070-146882570	16	500000	-45095	OTTMUSG00000010673
chr12:105760035-105760535	16	47410	-48840	Gsc
chr1:24740810-24741310	15	500000	500000	
chr1:61322995-61323495	15	500000	500000	
chr1:91146425-91146925	15	500000	500000	
chr2:10981705-10982205	15	500000	500000	
chr2:28152440-28152940	15	500000	500000	
chr2:76046935-76047435	15	500000	500000	
chr2:94364670-94365170	15	500000	500000	
chr3:25942165-25942665	15	500000	500000	
chr3:50706700-50707200	15	500000	500000	
chr3:55461770-55462270	15	500000	500000	
chr3:103250650-103251150	15	500000	500000	
chr3:110190750-110191250	15	500000	500000	
chr3:149377975-149378475	15	500000	500000	
chr4:58352150-58352650	15	500000	500000	
chr4:130417690-130418190	15	104	500000	
chr4:139660995-139661495	15	3970	500000	

chr4:145001420-145001920	15	500000	500000
chr4:150934360-150934860	15	500000	500000
chr4:152330540-152331040	15	500000	500000
chr5:33777005-33777505	15	0	500000
chr7:71802820-71803320	15	500000	500000
chr7:89484415-89484915	15	0	500000
chr7:129940250-129940750	15	500000	500000
chr8:35287145-35287645	15	500000	500000
chr8:77288580-77289080	15	500000	500000
chr8:81278270-81278770	15	500000	500000
chr8:82023710-82024210	15	500000	500000
chr8:86897350-86897850	15	40561	500000
chr8:90303580-90304080	15	833	500000
chr8:90306765-90307265	15	0	500000
chr8:91361920-91362420	15	500000	500000
chr8:94013025-94013525	15	500000	500000
chr9:22840450-22840950	15	500000	500000
chr9:56644775-56645275	15	151	500000
chr9:99770045-99770545	15	164	500000
chr9:99771195-99771695	15	95	500000
chr10:25876170-25876670	15	500000	500000
chr10:44652630-44653130	15	500000	500000
chr10:57154590-57155090	15	500000	500000
chr10:88849385-88849885	15	41755	500000
chr10:98656950-98657450	15	500000	500000
chr10:98659130-98659630	15	500000	500000
chr10:110702520-110703020	15	32720	500000
chr11:118556250-118556750	15	500000	500000
chr13:13952975-13953475	15	500000	500000
chr13:42309015-42309515	15	500000	500000
chr13:95477020-95477520	15	3140	500000
chr14:49188560-49189060	15	500000	500000
chr14:59472550-59473050	15	500000	500000
chr15:74084625-74085125	15	500000	500000
chr15:87233000-87233500	15	500000	500000
chr15:95829880-95830380	15	500000	500000
chr16:3351370-3351870	15	500000	500000
chr17:30858575-30859075	15	500000	500000
chr17:30885960-30886460	15	500000	500000
chr17:30888700-30889200	15	500000	500000
chr17:73868880-73869380	15	500000	500000
chr17:86177630-86178130	15	21532	500000
chr18:4234360-4234860	15	500000	500000
chr18:39716645-39717145	15	500000	500000
chrX:143717235-143717735	15	500000	500000
chrX:147604040-147604540	15	48	500000

chrX:158346350-158346850	15	0	500000	
chr7:75144000-75144500	15	45983	47108	Igf1r
chr4:145154550-145155050	15	12193	45965	BC086318
chr4:145152885-145153385	15	13858	44300	BC086318
chr4:153969850-153970350	15	38791	40374	5930403L14Rik
chrX:63941380-63941880	15	500000	35297	Slitrk2
chr17:33600560-33601060	15	500000	30967	1700029I08Rik
chr4:122750910-122751410	15	30701	30205	Trit1
chr19:7250920-7251420	15	10450	29603	Otub1
chr10:114893375-114893875	15	500000	29265	Ccdc131
chr10:122409690-122410190	15	12340	27801	AK021038
chr4:145135575-145136075	15	31168	26990	BC086318
chr4:107329160-107329660	15	26739	26771	Dmrtb1
chr15:79309665-79310165	15	5839	25755	Kcnj4
chr2:13167745-13168245	15	24881	24909	Rsu1
chr12:70551970-70552470	15	500000	24313	AK039829
chr15:89354970-89355470	15	507	23507	Shank3
chr2:94081650-94082150	15	131	23014	AK140370
chr11:74199355-74199855	15	500000	21433	Olfr412
chr1:57922915-57923415	15	500000	20751	2810022L02Rik
chr8:85946820-85947320	15	19018	19476	Scoc
chr7:142871685-142872185	15	35587	18835	Mki67
chr15:84040960-84041460	15	18292	18548	Samm50
chr7:103963315-103963815	15	500000	18415	ten-m4
chr11:115110195-115110695	15	758	18111	Grin2c
chr15:97816910-97817410	15	15161	17915	Col2a1
chr7:103962680-103963180	15	500000	17780	ten-m4
chr15:75560770-75561270	15	1014	17331	Mafa
chr17:30957060-30957560	15	500000	17303	Dnahc8
chr17:24192135-24192635	15	17663	17176	Prss27
chr18:44318470-44318970	15	500000	16006	AK020535
chr5:149979640-149980140	15	15799	15748	AK082086
chr8:87261260-87261760	15	21244	14605	AK168184
chr5:148783050-148783550	15	13719	14405	C130038G02Rik
chr7:24552350-24552850	15	500000	13671	V1rd22
chr15:75565140-75565640	15	2950	12961	Mafa
chr1:90147220-90147720	15	26318	12788	AK133186
chr4:100188105-100188605	15	500000	12050	AK085758
chr1:90146180-90146680	15	27358	11748	AK133186
chr2:130133080-130133580	15	11367	11656	Ebf4
chr19:43815665-43816165	15	11304	11555	Cox15
chr13:23504380-23504880	15	10743	11104	Abt1
chr15:77462440-77462940	15	500000	10626	EG626615
chr10:127349430-127349930	15	500000	10550	Sdro
chr1:90118635-90119135	15	16511	10352	Ugt1a1
chr6:126474840-126475340	15	500000	10339	Kcna5

chr7:26433895-26434395	15	9974	9610	Bckdha
chr10:41985790-41986290	15	8497	9363	Foxo3a
chr1:90163495-90163995	15	10043	9014	A730008H23Rik
chr2:153996650-153997150	15	500000	8557	2310021H06Rik
chr17:24062130-24062630	15	533	7956	Dcpp3
chr15:83723880-83724380	15	500000	7639	mKIAA1672
chr3:88949185-88949685	15	12878	7546	AK177718
chr1:90207030-90207530	15	33002	7423	Trpm8
chr15:58906310-58906810	15	6272	6983	Mtss1
chr11:114959265-114959765	15	6377	6771	Rab37
chr2:3259840-3260340	15	10063	6651	9230116B18Rik
chr11:114916790-114917290	15	35724	6311	Cd300e
chr1:133090265-133090765	15	40609	6159	Rassf5
chr7:149141495-149141995	15	5159	6090	mKIAA4256
chr5:135819650-135820150	15	5821	5844	Fkbp6
chr5:140389120-140389620	15	319	5474	A930017N06Rik
chr3:116666150-116666650	15	500000	5469	Palmd
chr3:128907780-128908280	15	439	5195	Pitx2
chr12:112718870-112719370	15	4241	4886	Gm266
chr10:83190055-83190555	15	4332	4663	AK005183
chr3:89563690-89564190	15	4380	4566	Chrnb2
chr4:128565555-128566055	15	3967	4422	Trim62
chr15:74350660-74351160	15	681	4285	Bai1
chr4:114736060-114736560	15	83	4187	Tal1
chr4:124667555-124668055	15	300	4132	Rspo1
chr8:24089090-24089590	15	3162	4025	Ank1
chr5:140023555-140024055	15	40	3954	Uncx4
chr8:123644070-123644570	15	2228	3838	Foxc2
chr15:84158695-84159195	15	500000	3796	Parvg
chr1:157585120-157585620	15	259	3786	Lhx4
chr3:100289190-100289690	15	2845	3665	Fam46c
chr11:5962040-5962540	15	2709	3460	Camk2b
chr11:101824775-101825275	15	93	3303	Sost
chr15:98623290-98623790	15	178	3253	Wnt1
chr17:26975070-26975570	15	256	3189	Nkx2-5
chr19:43459445-43459945	15	1022	3105	LOC545291
chr5:9103580-9104080	15	2378	3094	4930420K17Rik
chr8:79421205-79421705	15	1717	3064	AK162930
chr15:102164980-102165480	15	2772	3062	Myg1
chr18:75529730-75530230	15	1317	2962	Smad7
chr10:61598385-61598885	15	1999	2798	Neurog3
chr18:64502550-64503050	15	131	2783	Onecut2
chr9:102253970-102254470	15	1348	2742	Ephb1
chr2:179836050-179836550	15	1725	2626	Hrh3
chr5:140022190-140022690	15	27	2589	Uncx4
chr4:151760925-151761425	15	23614	2582	mKIAA0444

chr19:54122030-54122530	15	107	2531	Adra2a
chr15:78223575-78224075	15	12231	2489	Ean57
chr14:35635540-35636040	15	275	2469	Grid1
chr3:96331320-96331820	15	30030	2463	Hfe2
chr17:56128080-56128580	15	11621	2397	Fsd1
chr15:75917805-75918305	15	2640	2387	Nrbp2
chr8:123642590-123643090	15	748	2358	Foxc2
chr14:122871910-122872410	15	1070	2260	AK011684
chr5:31359165-31359665	15	1840	2232	Cad
chr10:119911575-119912075	15	150	2165	Hmga2
chr3:8664665-8665165	15	0	2122	Hey1
chr11:84336720-84337220	15	570	2065	Lhx1
chr15:84683335-84683835	15	308	1943	Phf21b
chr5:34340180-34340680	15	96	1798	Nat8l
chr4:28741830-28742330	15	0	1779	Epha7
chr11:118768860-118769360	15	154	1775	D11Bwg0517e
chr11:103636840-103637340	15	1354	1553	Wnt3
chr2:164542030-164542530	15	28949	1476	OTTMUSG00000016293
chr8:73018280-73018780	15	280	1476	Crlf1
chr19:54120960-54121460	15	0	1461	Adra2a
chr17:27694705-27695205	15	60	1429	Hmga1
chr10:57693790-57694290	15	24052	1382	Dux
chr8:94882725-94883225	15	0	1281	Irx5
chr11:32166095-32166595	15	993	1268	Mpg
chr12:28026065-28026565	15	0	1261	Sox11
chr2:102499810-102500310	15	557	1221	Slc1a2
chr17:46691655-46692155	15	34	1205	Srf
chr6:99643600-99644100	15	400	1178	Gpr27
chr1:173510120-173510620	15	500000	1177	2b4
chr11:68198960-68199460	15	0	1117	Ntn1
chr12:73335390-73335890	15	1814	1085	Rtn-1A
chr17:88196020-88196520	15	31	1063	Kcnk12
chr10:127534235-127534735	15	0	1054	Baz2a
chr4:84191025-84191525	15	0	1033	Bnc2
chr9:58005695-58006195	15	39845	1003	Islr
chr6:4698050-4698550	15	0	995	Peg10
chr11:18914625-18915125	15	4012	984	AK144295
chr12:53605685-53606185	15	0	977	Arhgap5
chr19:42165615-42166115	15	337	941	Pi4k2a
chr4:151713445-151713945	15	472	936	Chd5
chr16:11984815-11985315	15	0	860	BC030884
chr6:94649040-94649540	15	0	848	Lrig1
chr4:131603815-131604315	15	248	834	Epb4
chr8:35871240-35871740	15	0	827	Dusp4
chr4:48598630-48599130	15	84	816	Tmeff1
chr5:111238040-111238540	15	307	809	Ulk1

chr8:80959505-80960005	15	0	795	Pou4f2
chr1:64166920-64167420	15	1017	792	Klf7
chr4:118966080-118966580	15	0	787	Ybx1
chr12:70993115-70993615	15	105	784	Map4k5
chr13:101602350-101602850	15	20	781	Slc30a5
chr6:28210575-28211075	15	115	775	Zfp800
chr3:55986620-55987120	15	0	752	nbea
chr3:66024305-66024805	15	8	747	Ptx3
chr2:180191520-180192020	15	60	732	Slco4a1
chr10:19654770-19655270	15	105	732	Map3k5
chr10:60735210-60735710	15	415	725	Adamts14
chr10:51953880-51954380	15	0	706	Dcbld1
chr5:124133350-124133850	15	0	699	Clip1
chr11:96975650-96976150	15	0	688	Tbx21
chr14:48269420-48269920	15	0	685	Ktn1
chr15:26824385-26824885	15	24	683	Fbxl7
chr14:26279205-26279705	15	0	678	Zmiz1
chr11:117516705-117517205	15	0	677	Tnrc6c
chr2:32236480-32236980	15	292	673	1110008P14Rik
chr5:148082180-148082680	15	227	666	AK008755
chr7:86874030-86874530	15	7779	660	Plin
chr11:103635935-103636435	15	449	648	Wnt3
chr9:72123100-72123600	15	141	645	Suhw4
chr6:90413005-90413505	15	9	636	Klf15
chr17:51971090-51971590	15	0	617	Satb1
chr2:28048970-28049470	15	291	608	Olfm1
chr10:119913145-119913645	15	0	595	Hmga2
chr10:33926515-33927015	15	0	591	Dse
chr15:97737895-97738395	15	263	581	Vdr
chr5:37730070-37730570	15	436	574	Evc2
chr13:40823495-40823995	15	0	567	Tcfap2a
chr11:35583310-35583810	15	0	564	Pank3
chr7:140967690-140968190	15	225	545	Dhx32
chr19:42275825-42276325	15	0	545	Sfrp5
chr8:35217930-35218430	15	266	539	Tmem66
chr1:84835105-84835605	15	0	523	Trip12
chr19:42222160-42222660	15	0	521	Marveld1
chr1:14299510-14300010	15	68	519	Eya1
chr8:122081860-122082360	15	187	516	Mbtps1
chr9:69607880-69608380	15	0	503	Foxb1
chr14:18493720-18494220	15	303	497	Thrb
chr10:80640645-80641145	15	1096	495	Eef2
chr1:74938970-74939470	15	79	488	Cryba2
chr8:126544170-126544670	15	260	487	Taf5l
chr8:87189260-87189760	15	1227	484	AK037841
chr13:104967820-104968320	15	709	475	2410002O22Rik

chr5:111309065-111309565	15	0	472	AK009368
chrX:166411030-166411530	15	11576	464	Fxy
chr16:32099100-32099600	15	127	460	Pigx
chr2:113056520-113057020	15	352	448	Ryr3
chr14:120877880-120878380	15	0	448	Rap2a
chr8:90642895-90643395	15	177	447	Tmem188
chr2:5059015-5059515	15	126	444	Ccdc3
chr5:134789890-134790390	15	0	422	Gtf2i
chr8:112088830-112089330	15	235	420	Dhx38
chr1:90172105-90172605	15	1433	404	A730008H23Rik
chr11:115970675-115971175	15	0	404	Trim47
chr10:122701280-122701780	15	0	399	AI851790
chr14:21656060-21656560	15	42800	382	Plau
chr4:151665145-151665645	15	557	375	Hes3
chr5:144131890-144132390	15	204	370	Grid2ip
chr14:56380380-56380880	15	185	366	Ltb4r2
chr11:77030030-77030530	15	86	354	Ssh2
chr1:24593675-24594175	15	0	350	Col19a1
chr4:155577510-155578010	15	0	348	AK078660
chr6:81991755-81992255	15	77	339	Tmem166
chr1:5008950-5009450	15	0	259	Rgs20
chr13:55423420-55423920	15	0	255	Prelid1
chr2:19367280-19367780	15	0	241	Ptf1a
chr11:16157760-16158260	15	66	236	Vstm2a
chr4:40917960-40918460	15	0	235	Nfx1
chr11:76212140-76212640	15	0	235	Nxn
chr11:53583980-53584480	15	72	234	Irf1
chr1:193915965-193916465	15	0	229	Traf5
chr2:154233340-154233840	15	0	226	Snta1
chr8:83866430-83866930	15	103	226	Inpp4b
chr2:131855070-131855570	15	111	225	Rassf2
chr7:90015125-90015625	15	0	208	AK076849
chr19:34953490-34953990	15	4	201	Pank1
chr9:30729590-30730090	15	0	196	Adamts15
chr14:55195815-55196315	15	0	187	Jub
chr2:130276200-130276700	15	0	175	Ptpra
chr2:165980740-165981240	15	0	165	Sulf2
chr16:62854035-62854535	15	6	126	Pros1
chr12:52929980-52930480	15	0	109	Hectd1
chr17:29235010-29235510	15	113	109	Cdkn1a
chr16:58523070-58523570	15	0	104	St3gal6
chr12:85534635-85535135	15	0	94	C130039O16Rik
chr5:104121280-104121780	15	0	92	Aff1
chr12:85410110-85410610	15	103	86	Acot5
chr15:7347975-7348475	15	0	78	Egflam
chr3:120874285-120874785	15	500000	76	Rwdd3

chr19:21346585-21347085	15	0	68	Zfand5
chr13:93196870-93197370	15	124	58	AK047727
chr11:85645920-85646420	15	0	54	Tbx2
chr1:182571260-182571760	15	0	46	Lin9
chr13:37918570-37919070	15	0	20	Rreb1
chr15:27955340-27955840	15	0	12	Trio
chr1:40522150-40522650	15	500000	4	Il18r1
chr13:113836348-113836848	15	10965	0	2310016C16Rik
chr18:77094880-77095380	15	0	-13	Corl2
chr4:136604380-136604880	15	0	-21	Zbtb40
chr5:20207800-20208300	15	0	-44	AK028130
chr12:103367320-103367820	15	0	-58	Slc24a4
chr10:45297320-45297820	15	2	-124	Hace1
chr16:85173830-85174330	15	0	-133	App
chr2:29201920-29202420	15	126	-185	Med27
chr11:51102960-51103460	15	59	-211	Col23a1
chr3:61167965-61168465	15	180	-213	Rap2b
chr9:123587980-123588480	15	154	-281	Xtrp3s1
chr16:17069860-17070360	15	132	-293	Ypel1
chr15:30101800-30102300	15	63	-297	Ctnnd2
chr6:91361435-91361935	15	152	-323	Wnt7a
chr13:31717035-31717535	15	82	-362	Foxf2
chr16:22163485-22163985	15	299	-364	Igf2bp2
chr18:24867330-24867830	15	152	-365	FHOS2
chr11:114712515-114713015	15	0	-390	Gprc5c
chr1:97564330-97564830	15	236	-410	ST8SiaIV
chr6:38500690-38501190	15	102	-578	Luc7l2
chr11:98244240-98244740	15	1040	-634	Tcap
chr11:103509880-103510380	15	697	-692	Rprml
chr15:76353010-76353510	15	0	-702	Scrt1
chr11:116513820-116514320	15	896	-777	Prcd
chr10:24316145-24316645	15	504	-778	AK014660
chr4:47005140-47005640	15	333	-805	Gabbr2
chr5:65193700-65194200	15	0	-811	Klf3
chr8:11727105-11727605	15	80	-813	Arhgef7
chr12:54348600-54349100	15	488	-813	Npas3
chr4:128287850-128288350	15	19372	-823	Zscan20
chr8:91568655-91569155	15	201	-845	Sall1
chr7:152023430-152023930	15	144	-932	Fgf3
chr4:153515005-153515505	15	327	-939	Trp73
chr4:151503800-151504300	15	740	-1017	Espn
chr10:114239210-114239710	15	203	-1041	Trhde
chr9:62997620-62998120	15	652	-1103	Lbxcor1
chr5:48374625-48375125	15	0	-1156	Slit2
chr16:32429670-32430170	15	783	-1161	Pcyt1a
chr2:180625365-180625865	15	590	-1198	AK045142

chr15:79336910-79337410	15	586	-1490	Kcnj4
chr8:122753370-122753870	15	0	-1491	AK039624
chr16:90384865-90385365	15	863	-1526	Hunk
chr2:151967480-151967980	15	1219	-1614	Tcf15
chr4:21614510-21615010	15	813	-1651	Prdm13
chr14:51708940-51709440	15	500000	-1680	Ang
chr4:125165770-125166270	15	1174	-2054	Grik3
chr10:94409160-94409660	15	1210	-2199	Plxnc1
chr17:26247650-26248150	15	2098	-2361	Tmem8
chr10:126619980-126620480	15	2699	-2593	C78409
chr8:19919265-19919765	15	26335	-2645	AK166824
chr14:63800855-63801355	15	4093	-2691	Fdft1
chr17:29748455-29748955	15	2957	-3070	Rnf8
chr3:102270420-102270920	15	3176	-3180	Ngfb
chr14:33845880-33846380	15	500000	-3968	Wdfy4
chr4:22419050-22419550	15	1606	-4023	Pou3f2
chr18:36009580-36010080	15	10324	-4514	Cxxc5
chr8:89108915-89109415	15	38818	-4581	Abcc12
chr5:112825720-112826220	15	1919	-4738	Asphd2
chr15:99496145-99496645	15	4294	-4753	Accn2
chr11:76480750-76481250	15	890	-4971	A830053O21Rik
chr3:96142805-96143305	15	10719	-6154	AK135774
chr12:104130950-104131450	15	14336	-6315	Cox8c
chr9:108172565-108173065	15	6556	-6561	Dag1
chr3:89160750-89161250	15	6591	-7069	Adam15
chr3:88003230-88003730	15	953	-7254	AK141565
chr1:157596340-157596840	15	6215	-7434	Lhx4
chr1:173473175-173473675	15	39094	-8002	Itln1
chr9:114588550-114589050	15	8785	-9148	Dync1li1
chr2:77127650-77128150	15	8930	-9252	Sestd1
chr17:34796590-34797090	15	11267	-10639	Tnxb
chr18:31782435-31782935	15	10951	-11351	Sap130
chr17:15057635-15058135	15	22053	-11362	Wdr27
chr3:93618280-93618780	15	500000	-11411	Tdpoz3
chr2:180221930-180222430	15	12405	-12500	Ntsr1
chr1:90187010-90187510	15	12982	-12597	Trpm8
chr4:62862900-62863400	15	11846	-13295	Col27a1
chr5:37393505-37394005	15	13093	-13535	Wfs1
chr4:135394900-135395400	15	16356	-16856	nssr 1
chr19:55236890-55237390	15	500000	-18234	Tectb
chr3:96130510-96131010	15	1302	-18449	AK135774
chr11:49696180-49696680	15	18654	-18906	Rasgef1c
chr3:40407310-40407810	15	22871	-22647	Pdzd6
chr10:116753145-116753645	15	500000	-23472	Lyz
chr4:145028725-145029225	15	24469	-25137	NR_002888
chr8:22139685-22140185	15	500000	-25288	Defcr21

chr1:155561530-155562030	15	500000	-25723	Rgs16
chr15:83581295-83581795	15	25877	-26095	Scube1
chr18:32137535-32138035	15	38756	-28496	Gpr17
chr8:85357850-85358350	15	29239	-29154	mKIAA1214
chr18:36226870-36227370	15	653	-29154	AY227026
chr2:145582000-145582500	15	500000	-29632	Rin2
chr6:94418935-94419435	15	30931	-31156	Slc25a26
chr11:95480170-95480670	15	26441	-31409	Ngfr
chr3:32296340-32296840	15	0	-32004	Zmat3
chr10:87998430-87998930	15	500000	-32071	Chpt1
chr11:5084060-5084560	15	31749	-32086	Emid1
chr4:145020285-145020785	15	32909	-33577	NR_002888
chr12:88103065-88103565	15	35807	-35915	Angel1
chr10:84342810-84343310	15	37199	-37300	Ric8b
chr7:146210425-146210925	15	1951	-37830	mKIAA4091
chr17:30065420-30065920	15	38786	-40844	Mdga1
chr16:92740100-92740600	15	41215	-42778	Runx1
chr4:3155580-3156080	15	2307	-43379	LOC171266
chr10:81748050-81748550	15	500000	-44276	B230315N10Rik
chr4:149942780-149943280	15	328	-47837	Rere
chr10:57157500-57158000	15	48245	-48541	Hsf2
