# Supplementary material for: ZBED6, a Novel Transcription Factor Derived from a Domesticated DNA Transposon Regulates IGF2 Expression and Muscle Growth
Source: PLoS Biol. 2009 Dec 15;7(12):e1000256. doi: 10.1371/journal.pbio.1000256 (PMC2780926; doi:10.1371/journal.pbio.1000256)
Supplement: Table S2 — Primers and probes for real-time PCR analysis of Zbed6 , Igf2 , and Zc3h11a . (0.03 MB DOC) [file pbio.1000256.s005.doc]

**Table S2. Primers and probes for real-time PCR analysis of *Zbed6*, *Igf2* and *Zc3h11a***

| Primer/Probe | Target Gene | Sequence |
| --- | --- | --- |
| Primer_For | *Igf2* | 5'-CGTGGCATCGTGGAAGAGT-3' |
| Primer_Rev |  | 5'-ACACGTCCCTCTCGGACTTG-3' |
| Probe |  | 5'-FAM-CTGGCCCTCCTGGAG-NFQ-MGB-3' |
| Primer_For | *Zbed6* | 5'-CAAGACATCTGCAGTTTGGAATTT-3' |
| Primer_Rev |  | 5'-TGTCGTTGAAGTGTTGAAGTTCCTA-3' |
| Probe |  | 5'-FAM-ACATCTCAAGAGCTGTGTGT-NFQ-MGB-3' |
| Primer_For | *zc3h11a* | 5'-TTGTCATCGGTTCGGTAAAGTTT-3' |
| Primer_Rev |  | 5'-CATCTGTGTCTTCACTCAGTTCCAA-3' |
| Probe |  | 5'-FAM-TGTTCTCTGGCGTAATAG-NFQ-MGB-3' |
| Primer_For | *18S* | 5'-AGTCCCTGCCCTTTGTACACA-3' |
| Primer_Rev |  | 5'-GATCCGAGGGCCTCACTAAAC-3' |
| Probe |  | 5'-FAM-CGCCCGTCGCTACTACCGATTGG-TAMRA-3' |
